# Supplementary material for: Cross-tissue transcriptome-wide association studies identify susceptibility genes shared between schizophrenia and inflammatory bowel disease
Source: Commun Biol. 2022 Jan 20;5:80. doi: 10.1038/s42003-022-03031-6 (PMC8776955; doi:10.1038/s42003-022-03031-6)
Supplement: Supplementary file 2 — Supplementary Information [file 42003_2022_3031_MOESM2_ESM.pdf]

# Supplementary Information

Cross-tissue transcriptome-wide association studies identify susceptibility genes shared between schizophrenia and inflammatory bowel disease

**Supplementary Notes ........................................................................ 3**

Supplementary Note 1 ........................................................................ 3

Supplementary Note 2 ........................................................................ 5

Supplementary Note 3 ........................................................................ 5

Supplementary Note 4 ........................................................................ 6

Supplementary Note 5 ........................................................................ 7

Supplementary Figures ........................................................................ 10

**Members of the International Inflammatory Bowel Disease Genetics Consortium (IIBDGC) ........................................................................ 56**

**Members of the International PSC Study Group (IPSCSG) ........................ 63**

**References ........................................................................ 66**

# Supplementary Notes

## Supplementary Note 1

**Demonstrating the utility of the GWAS/TWAS fine-mapping analysis across the 23 tissues of the GBA using GTCA-COJO by replicating known eQTL associations from recent fine-mapping GWAS studies for SCZ and CD/UC**

First, we demonstrated the utility of the GTCA-COJO[1] approach by examining established eQTL associations of GWAS signals as reported in the recent fine-mapping GWAS studies for CD/UC[2,3] (**Supplementary Fig. 6**), and also provided an example of a negative control by further applying our approach further to a well-established GWAS locus containing functional coding variants (**Supplementary Fig. 7**).

*INPP5E* was well replicated and is the strongest gene-disease signal in the same tissue as shown by Huang *et al.*[3] (**Supplementary Fig 6**). In CD, *CARD9* is also transcriptome-wide significant, which has been reported previously, but in a different tissue, which is not surprising as eQTLs often correlate across different tissues. GWAS association can be fully explained by eQTLs of *INPP5E*, but *CARD9* may also play a role.

We aimed to replicate this finding using our joint GWAS/TWAS fine-mapping COJO conditioning approach to demonstrate its usefulness. It was designed to detect whether eQTLs of a single gene are a possible explanation for local GWAS association. We consider a gene as the most likely candidate if its marginal and conditional TWAS score is trancriptome-wide significant, no other gene is more significant, and conditioning GWAS scores on eQTLs strongly reduces the association. If any of the conditions are not met, the gene may still be causal, but this indicates that other explanations need to be considered.

In the top panel (**Supplementary Fig. 6**), GWAS summary statistics are shown in grey and the COJO-conditioned GWAS results are shown in black. Red (pre-conditioning) and green (post-conditioning) circles are used to mark the 95% credible set from Huang *et al.*[3]. Asterisks denote eQTLs variants of the gene in focus (here *INPP5E*). From these, a set of independent variants was selected with COJO –select (**Methods**). The selected variants are annotated with a $r^2$ LD-value relative to the most significant eQTL variant in GWAS. In the middle panel, the eQTLs of the tissue and the other genes in the locus are marked. In the bottom panel are the *P*-values of the single-gene UTMOST test (Single-tissue$_{unconditional}$) and the multiple gene regression approach (Single-tissue$_{conditional}$) of the genes in the locus.

As a negative control and to demonstrate the utility of conditioning on eQTLs, we analyzed *NOD2*, a locus with well-studied coding variants in CD known to be causal for this association (**Supplementary Fig. 7**). Based on the linkage disequilibrium (LD) of the eQTLs with the coding variants, there are significant TWAS associations of *NOD2, SNX20, ADCY7* and *HEATR3*. By conditioning the GWAS summary statistics on seven independent proxy eQTLs of "Whole Blood" tissue, the GWAS signal cannot be fully explained, instead several variants remain significant. This suggests a non-eQTL component even without prior knowledge of the locus.

## Supplementary Note 2

### Detailed TWAS locus plots

The detailed plots (**Supplementary Figures 8-37**) show the loci of candidate genes in all tissues for which a gene expression model was available. The figures were designed to give an overview of the level of evidence of association for a particular gene, tissue and disease. **Figure 3** and **Supplementary Figures 38-41** are derived from these figures by masking out all other genes in the locus for a better overview. Nevertheless, comparing the eQTL distributions and the significance of other genes in the same locus may influence the interpretation of a candidate gene. Note that loci differ in terms of the number and significance of other genes in the locus, LD structure, density of SNPs and number and distribution of eQTLs, etc., which may influence the ratio of $TWAS_{marginal}$ to $TWAS_{conditional}$ *P*-values. To allow the reader to explore the loci themselves, we included the GWAS statistics at the locus and conditioned them on significant eQTLs using GCTA COJO[1] to visualize the amount of signal that could explain the eQTLs. In addition, we showed the location and weighting of the eQTLs as provided by UTMOST, as well as the marginal and conditional p-values of the UTMOST single-tissue test.

The top panel of each plot shows the GWAS summary statistics in grey and the conditional GWAS results in black. Red (pre-conditioning) and green (post-conditioning) circles are used to mark the 95% credible fine-mapped SNP set from Huang *et al.*[3] (UC, CD) and from Pardiñas *et al.*[4] (SCZ). Asterisks denote eQTLs of the gene of interest indicated in the header. From these, a set of independent variants was selected with COJO -select. The selected variants are annotated with an $r^2$ LD value relative to the most significant eQTL variant in GWAS. In the middle, the tissue eQTLs are shown along with the other genes on the locus. In the bottom panel, the *P*-values of the single-gene UTMOST test ($Single\text{-}tissue_{unconditional}$) and the multiple gene regression approach ($Single\text{-}tissue_{conditional}$) of the genes in the locus.

See **Supplementary Figures 8-37.**

## Supplementary Note 3

*Nuclear receptor subfamily 5 group A member 2* (*NR5A2*), encodes a transcription factor (also known as LRH-1) implicated in various cancers[5-9] and also regulates T cell functions[10] and intestinal glucocorticoid synthesis[11-14]. Human LRH-1 (hLRH-1) rescues epithelial integrity, attenuates inflammatory damage in murine and human intestinal organoids, including those derived from IBD patients, and reduces disease severity in T-cell-mediated

murine colitis[15]. *NR5A2* is also a critical regulator of neural development, also in the murine hypothalamus[16,17], and has been reported to be differentially expressed in post-mortem samples from the prefrontal cortex of SCZ patients[18]. The expression of *NR5A2* in different tissues and developmental stages is shown in **Figure 4** and discussed in the **Discussion**.

*SATB Homeobox 2* (*SATB2*) (2q33.1) is expressed primarily in colon and brain tissues[19]. It has functions in development[20-22] and is a marker for metastasis and prognosis in colorectal cancer[23,24] whose incidence is enriched in UC patients. Recent findings suggest that colitis-associated colorectal cancer is different from sporadic cases, as indicated molecularly by lower *SATB2* expression[25-27]. *SATB2* common variants and genes downstream of *SATB2* confer risk for SCZ[28-31]. *SATB2* is specifically upregulated in the cortex during midfetal development[31] which is accompanied by an increase in expression in the fetal forebrain, as we could visualized in **Figure 4a**. Further details and considerations can be found in **Figure 4b, Supplementary Figure 42** and in the **Discussion**.

*PPP3CA* (4q24) encodes a subunit of calcineurin[32] that plays a role in T cell activation and IL2-signaling via the transcription factor NFAT[33-35]. Calcineurin is a drug target in many diseases including rheumatoid arthritis[36], systemic lupus erythematosus[37], and ulcerative colitis[38]. Calcineurin has been found to play a role in schizophrenia[39,40] calcineurin signaling may contribute to the pathogenesis of schizophrenia or the development of schizophrenia-like symptoms[41] and general alterations in postmortem SCZ patient brains are controversial[42-44]; they are probably tissue specific. More calcineurin immunoreactive neurons were found in the caudate nucleus[45,46]. The caudate nucleus, together with the putamen, forms the striatum, where we found the shared association of *PPP3CA* with CD and SCZ. Baseline expression of *PPP3CA* is shown in **Figure 4**, where we show data[47,48] that expression of *PPP3CA* is particularly high in Paneth cells and enteroendocrine cells (**Figure 4b**). These cells are known to function as nervous system of bacterial metabolites and as part of gut immunoregulation, with evidence of implication in IBD[49], which fits the role of *PPP3CA* in the above-mentioned tissues.

## Supplementary Note 4

*SF3B1* (2q33.1) encodes a core spliceosome protein, its mutations are involved in various cancers[50-53] and gene expression alterations have been found in animal models of psychosis[54]. Interestingly *SF3B1* gene expression levels are also associated with genes of the innate immune response which in turn can affect susceptibility to inflammatory disease[55].

*INO80E* (16p11.2) protein is part of the INO80 chromatin remodeling complex and a SUMO-binding protein[56]. The INO80 complex is implicated in regulation of transcription, DNA replication, recombination and repairing DNA damage[57]. *INO80E* has not been covered by colitis research. It has been reported as a pleiotropic gene of SCZ and cardiometabolic disease[58]. It should be noted that *INO80E* might be a false positive association. It is 108 kb away from *MAPK3* which has been prioritized in SCZ by means of TWAS[59] and in IBD by means of a rat model and protein-protein interaction network analysis[60,61].

*SGSM3* (22q13.1) encodes a modulator of the small G protein signaling pathway. Knowledge about its implications in disease is scarce, but comprise diverse cancers[62-64], and myocardial infarction[65,66]. We found no literature on *SGSM3* in the context of CD, UC or SCZ.

*ZC3H7B* (22q13.2) encodes a protein that has been found by its interaction with a rotavirus protein and eIF4G[67], but current research focuses on the *ZC3H7B-BCOR* gene fusion relevant in endometrial sarcomas[68,69].

## Supplementary Note 5

### Baseline expression data of $GBJ_{conditional}$ genes and discussion

Here, we discuss the baseline expression reference data for the $GBJ_{conditional}$ genes, *SF3B1*, *INO80E*, *SGSM3* and *ZC3H7B* (**Supplementary Fig. 42**).

*SF3B1* (32;126;245 TPM min; median; max in GTEx data) is a component of the spliceosome and ubiquitously expressed. This is also true for blood cells (68;148;400 TPM in BLUEPRINT data). In development, *SF3B1* expression decreases with age, with no strongly favored organ. In single-cell data we observe a frequent and strong expression of *SF3B1* transcripts in every cell type in ileum, colon and rectum and a slightly less in and brain.

*INO80E* (11;34;93 TPM min; median; max in GTEx data) is predominantly expressed in testis, weaker in gastrointestinal tissues (40 TPM transverse colon) and rare in brain tissues, also in single cell data. It is rare in blood (2;9;38 TPM in BLUEPRINT data). This is in line with the available developmental data. The most prominent observation is an increase of expression in testis. In gut single cell data, *INO80E* is weakly to medium expressed in almost all cell types.

According to GTEx data of the brain tissues, *SGSM3* (23;63;154 TPM min; median; max in GTEx data) is expressed strongest in the cerebellum (126 TPM). The expression in gastrointestinal tissues is slightly weaker (colon transversus 77 TPM). It is also expressed in blood cells (4;21;79 TPM in BLUEPRINT data). There is no obvious expression tendency in the developmental expression data of *SGSM3*. The gut single-cell data shows that *SGSM3*

expression is slightly biased to goblet cells, although it is at least weakly expressed in colon and rectum cell types, it is much rarer in the ileum except in goblet cells and Paneth cells. In brain single-cell data, *SGSM3* is weakly to moderately in all cell types, but cerebellar tissue is not included in the experiment.

For *ZC3H7B* (6;47;108 TPM min; median; max in GTEx data), the strongest expression of the brain tissues can be found in the cerebellum (101 TPM). Its expression is weaker in gastrointestinal tissues (37 TPM in colon transversus). In blood cells, all cells except neutrophils express it stronger than 1 TPM (0.5;11;54 TPM in BLUEPRINT data). The expression of *ZC3H7B* shows no obvious tendency, but its general expression across all tissues seems to decrease with age while keeping a very low level of forebrain expression in adults. The transcript is rarely expressed in the single cell data of the ileum, colon and rectum and brain.

These four genes (*SF3B1*, *INO80E*, *SGSM3* and *ZC3H7B*) do not offer obvious (literature- and expression data based) IBD-associated or SCZ-associated hypotheses like the three main findings (Single-tissue$_{\text{conditional}}$ genes *NR5A2*, *SATB2*, *PPP3CA*). None of them are mentioned in the literature with direct reference to inflammatory bowel disease or schizophrenia (see **Supplementary Note 4**). We also find no genes specifically expressed in these tissues, which would support specific relevance to the gut-brain axis. In summary, despite its non-specific expression pattern, *SF3B1* is a candidate for a Crohn's disease and schizophrenia comorbidity gene, as it appears to colocalize with a GWAS signal (see **Supplementary Fig. 38**). Since it is a cancer-associated gene, it is at least conceivable that its up- or down-regulation may also have effects on cell metabolism in a non-cancer setting. *INO80E* may be a false positive because, first, to our knowledge, there is no literature suggesting a role in the gut-brain axis, nor is there a supportive expression pattern. Second, it is a neighbour of *MAPK3*, which has been linked to schizophrenia by a previous TWAS[59] and, in addition to *INO80E*, by a comorbidity study between schizophrenia and heart disease (see **Supplementary Note 4**), suggesting a strong link between the eQTLs of both genes. *SGSM3* and *ZC3H7B* are within 1Mb of physical distance to each other and thus, in terms of a TWAS study, at the same *cis* expression same locus. In addition, there are six other GBJ-significant genes in Crohn's disease and one in schizophrenia at this locus (see **Supplementary Data 14**), so we would suggest interpreting this finding as a potential comorbidity locus, without a clear gene hypothesis so far.

We consider *INO80E* to be a false positive because, first, its expression is restricted to the testis and it has not been implicated in any research related to inflammation or psychiatric

features. Secondly, it is on the same locus as *MAPK3*, which has been associated with SCZ in previous TWAS studies. The linkage between the TWAS associations of these genes is also already known (see **Supplementary Note 4**). Further, *SGSM3* and *ZC3H7B* are in the same locus and as described in the Results section, there are too many genes in the locus that are also suggestive to definitively disentangle this locus with the TWAS method applied (see **Supplementary Notes 1-2**). Nevertheless, we would like to suggest *EP300*, which is GBJ-significant in CD, as the most likely candidate from a biological point of view, as it is part of the "calcineurin-dependent NFAT signalling pathway in lymphocytes".

## Supplementary Figures

**Supplementary Figure 1. Schematic overview of the study workflow**. The flowchart shows the study design and analytic strategy of both the TWAS discovery phase and the downstream analyses. The PubMedIDs in parentheses refer to the original GWAS studies.

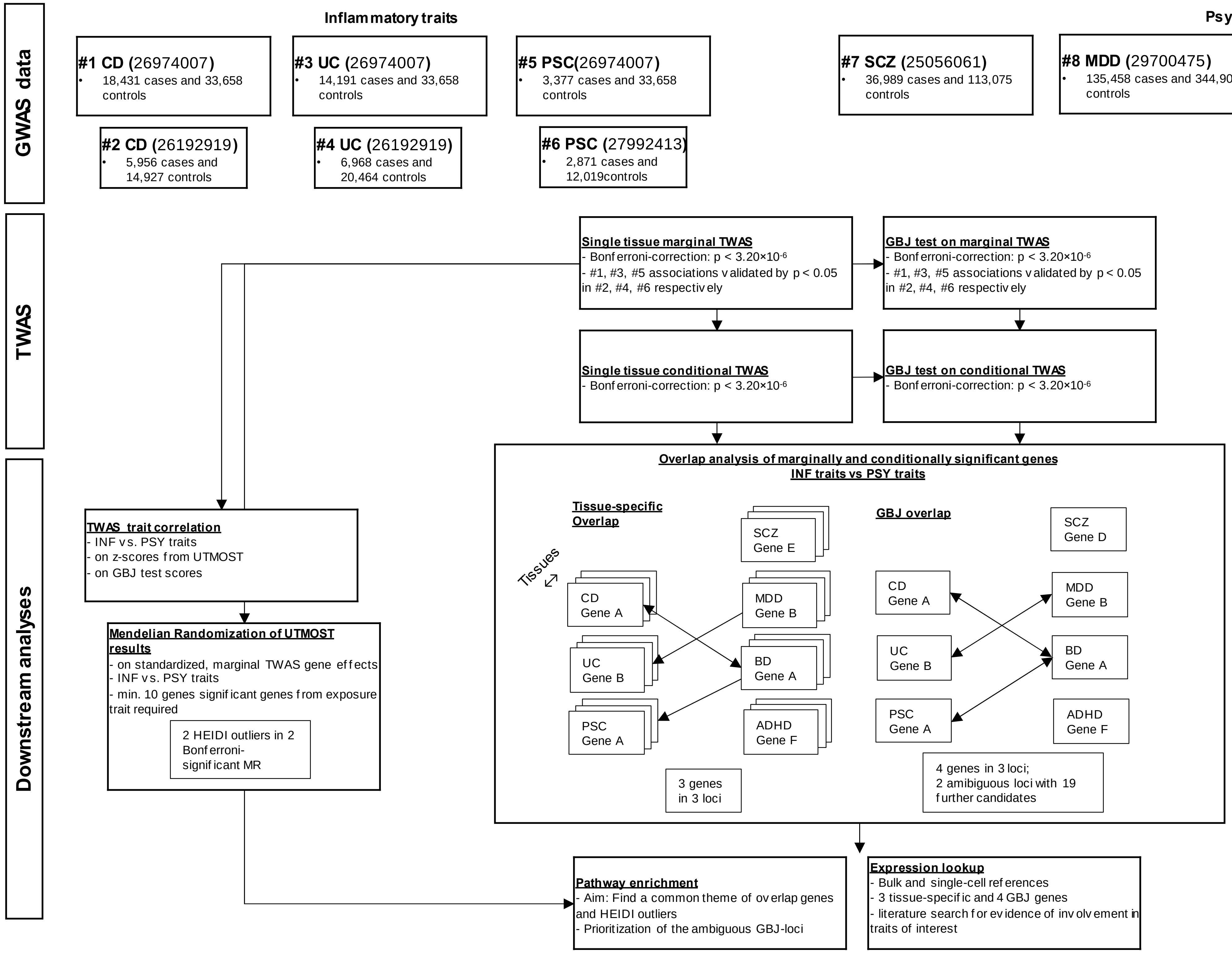


Workflow of the present comparative TWAS study. PMIDs of the GWAS are given in brackets. Abb. ADHD: Attention deficit hyperactivity syndrome, BD: Bipolar disease, CD: Crohn's disease, GBJ: Generalized Berk-Jones (test), GWAS: Genome-wide association study, INF: inflammatory, MDD: major depressive disorder, PSC: primary sclerosing cholangitis, PSY: psychiatric, SCZ: Schizophrenia, TWAS: Transcriptome-wide association study, UC: Ulcerative colitis

**Supplementary Figure 2. Manhattan plots of (marginal i.e. primary and unconditioned;** $GBJ_{marginal}$**) transcriptome-wide association analysis (TWAS) results across 23 tissues of the gut-brain-axis (GBA).** For selection of tissues, see **Methods.** CD, Crohn's disease; UC, ulcerative colitis; PSC, primary sclerosing cholangitis; SCZ, schizophrenia; MDD, major depressive disorder; BIP, bipolar disease (BIP); ADHD, attention-deficit/hyperactivity disorder (ADHD); grey dotted line, transcriptome-wide significance threshold of $3.20\times10^{-6}$ (**Methods**); red dots, gene-disease associations meeting the transcriptome-wide significance threshold in the unconditioned analysis (**Methods**).

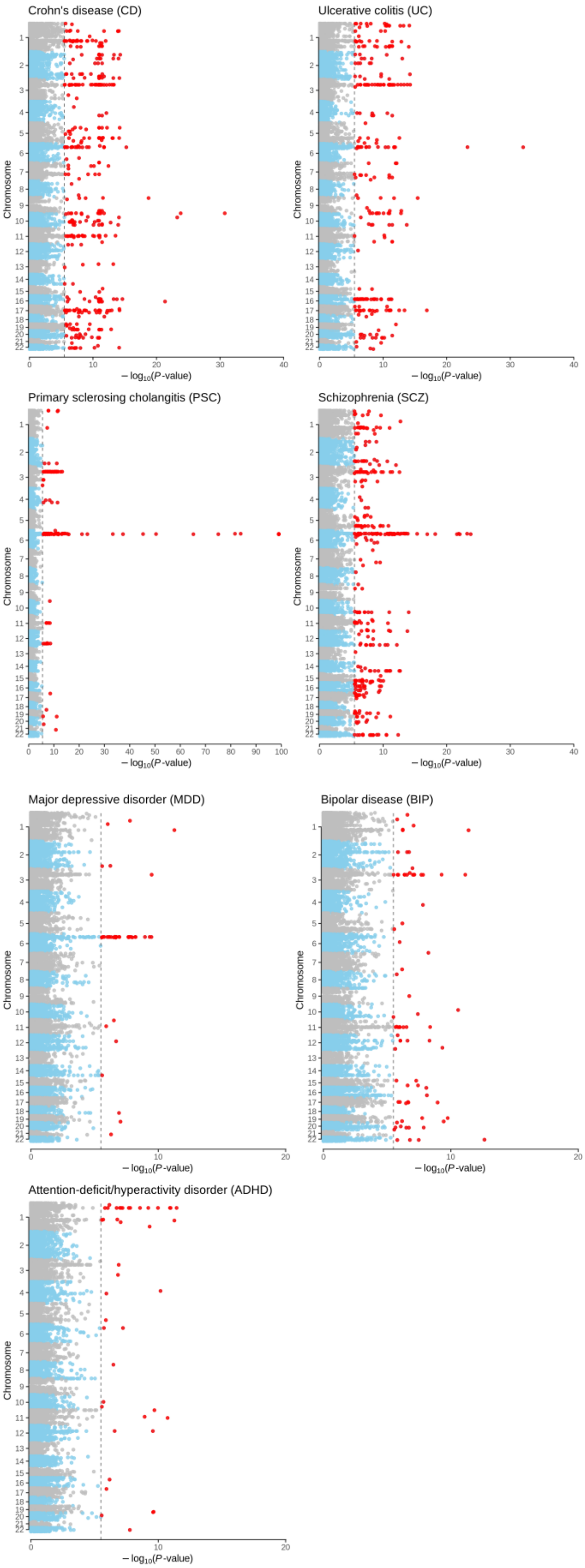
Crohn's disease (CD)
Ulcerative colitis (UC)
Primary sclerosing cholangitis (PSC)
Schizophrenia (SCZ)
Major depressive disorder (MDD)
Bipolar disease (BIP)
Attention-deficit/hyperactivity disorder (ADHD)
Chromosome
$-\log_{10}(P\text{-value})$

**Supplementary Figure 3. Only a moderate correlation (as expected) was observed between sample size and number of significant genes (Bonferroni correction for number of genes tested) per tissue from (A) UTMOST analysis as compared to single-tissue imputation-based TWAS association results from (B) S-PrediXcan/MetaXcan[70] and (C) FUSION[71] (see also Supplementary Data 4).** The slopes ($\beta$) from best-fitting least-squares regression lines (**Supplementary Data 4**) were generally smaller in UTMOST compared to S-PrediXcan (p=0.0020, paired Mann-Whitney U-test) and FUSION (p=0.0039, paired Mann-Whitney U-test, see **Methods**). The GWAS case-control data sets correspond to the list of data sets presented in **Supplementary Data 1**.

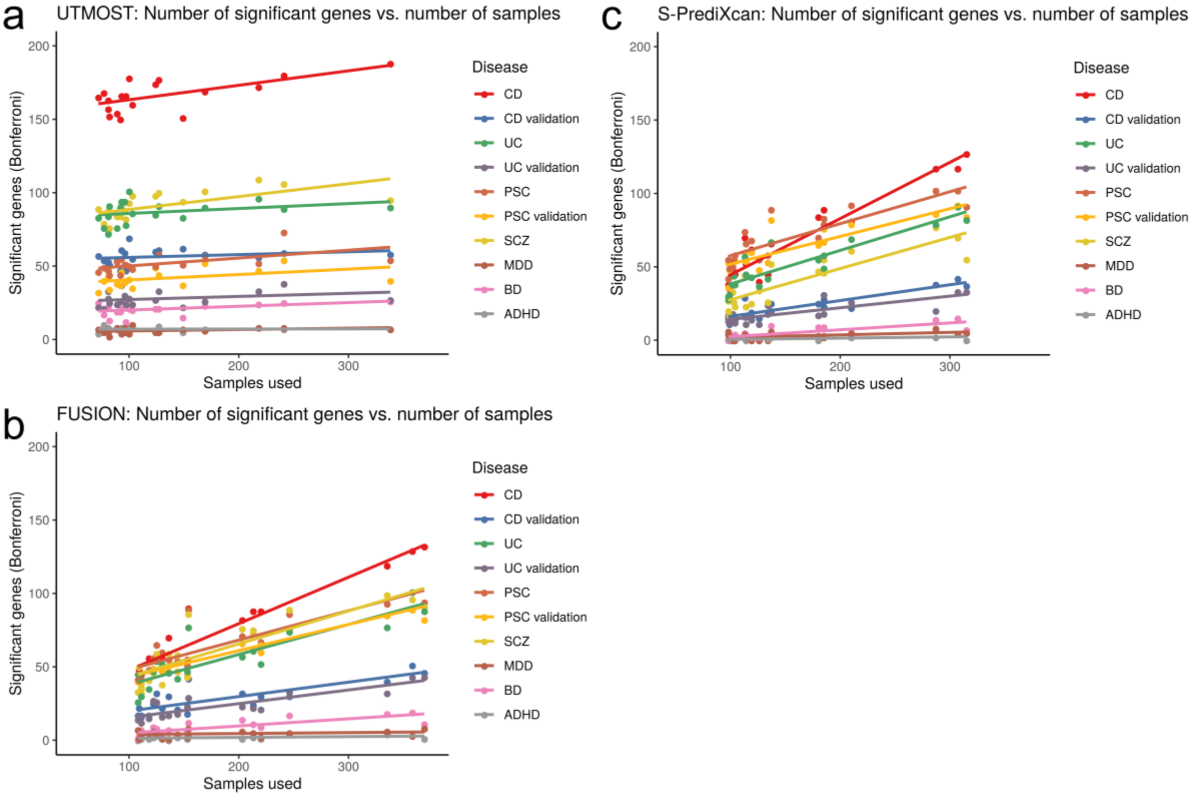

**Supplementary Figure 4. SCZ and CD/UC are weakly but significantly correlated on the genome-wide genetic level (LDSC $r_g$).** BD and CD/UC however miss significance. Asterisks denote statistical significance as follows: */**/*** p<0.05/p<0.01/p<0.001. rg: LDSC $r_g$, a measure of genetic correlation.

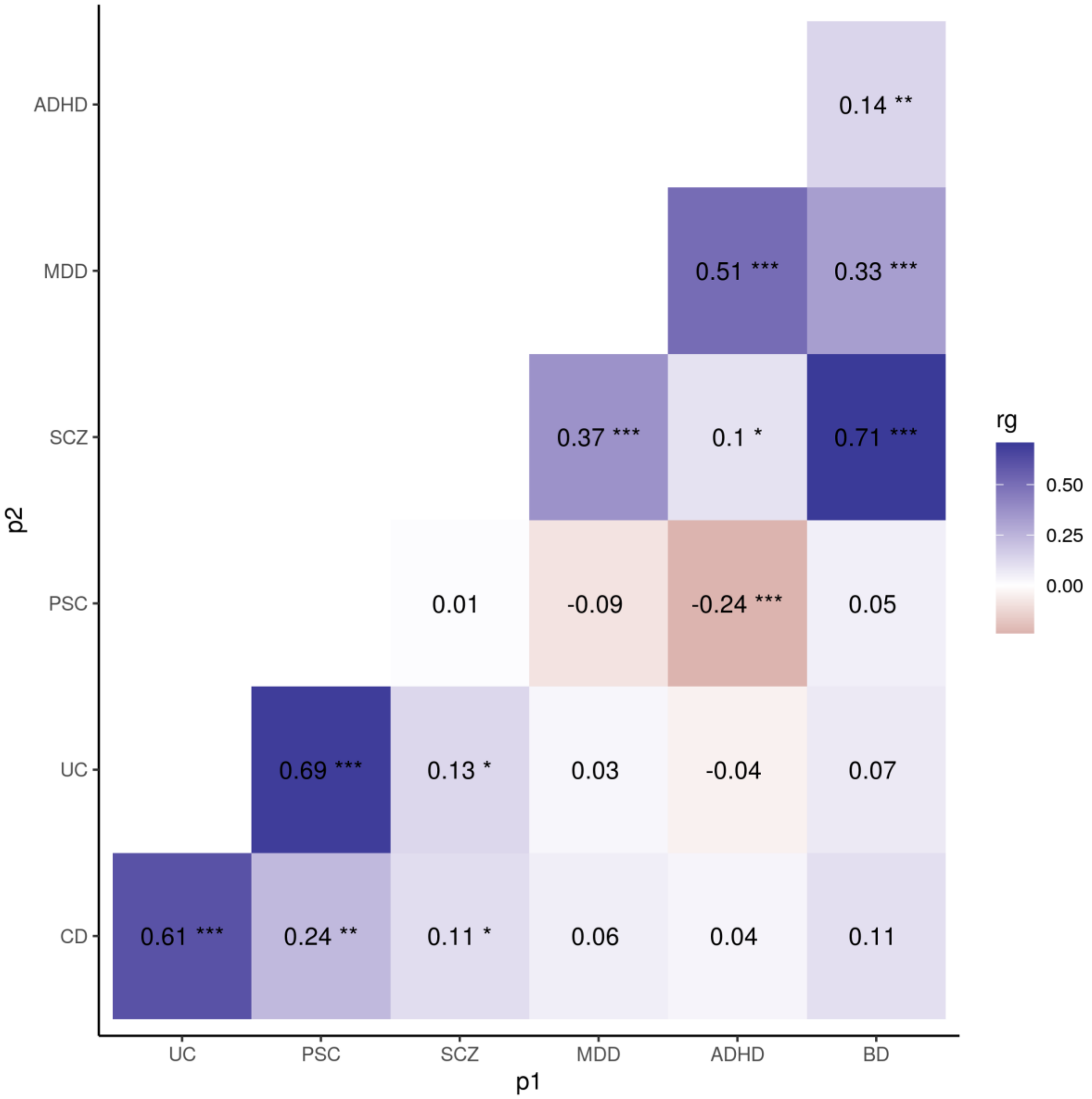

**Supplementary Figure 5. Strongest positive genetic trait correlation (Single-tissue$_{marginal}$) across psychiatric and immune phenotypes was observed for CD4$^{+}$ T-cells.** Genetic trait correlation results for all 23 tissues are given in **Supplementary Data 9,10**. rg: Spearman correlation of z scores for TWAS results.

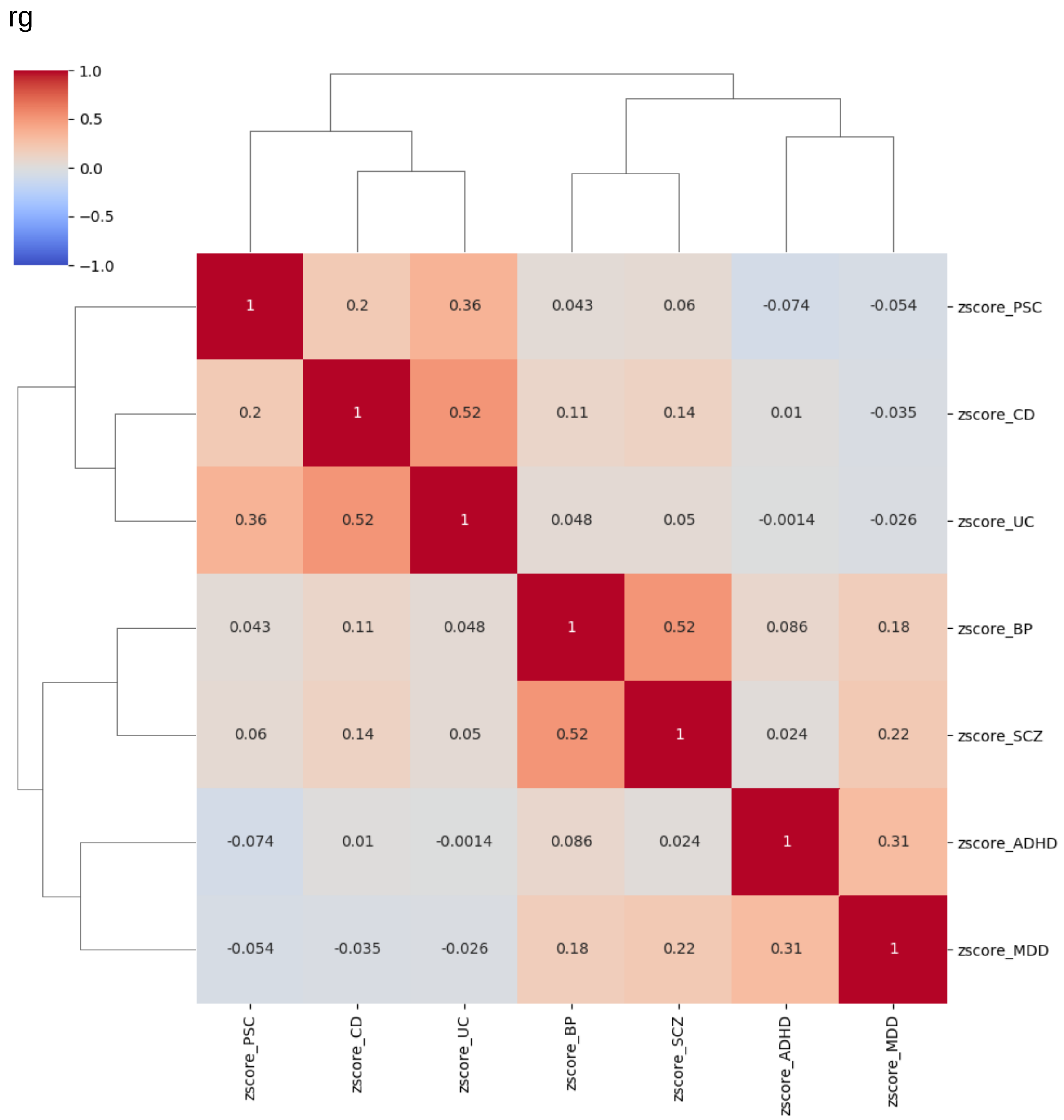

**Supplementary Figure 6. Demonstration of the utility of our joint GWAS/TWAS fine-mapping COJO conditioning approach across the 23 tissues of the GBA (Methods) by replicating known eQTL associations from the latest fine-mapping GWAS study for CD and UC[3].** Co-localization of fine-mapped risk variants with eQTLs in CD4 and CD8 T cells for gene *INPP5E* have been identified in the latest fine-mapping GWAS study for CD (**a**) and UC (**b**).

**a**

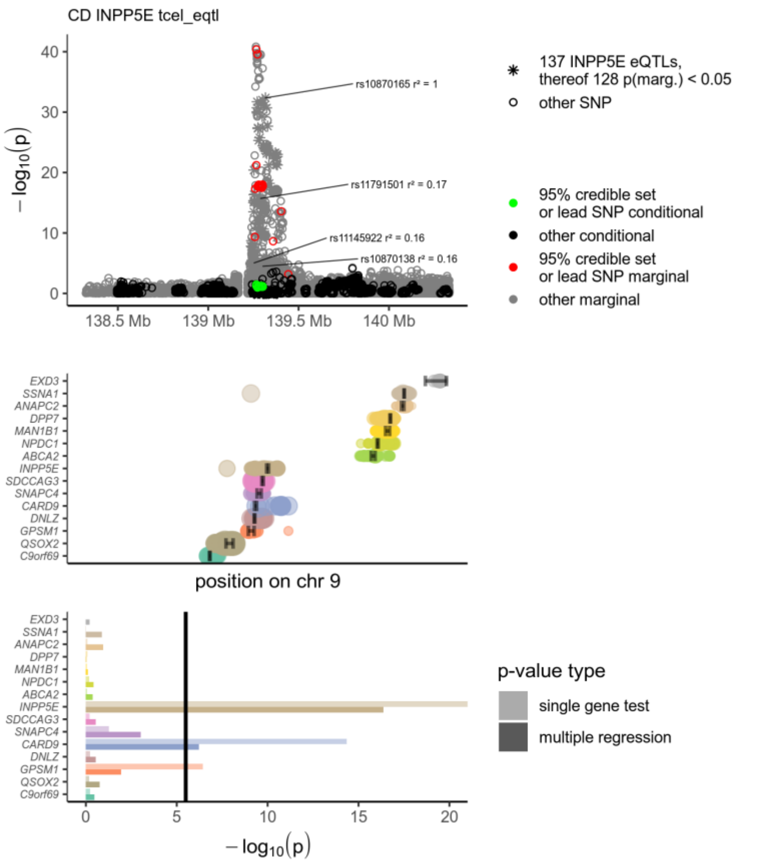
CD INPP5E tcel_eqtl
137 INPP5E eQTLs, thereof 128 p(marg.) < 0.05
other SNP
95% credible set or lead SNP conditional
other conditional
95% credible set or lead SNP marginal
other marginal
rs10870165 r² = 1
rs11791501 r² = 0.17
rs11145922 r² = 0.16
rs10870138 r² = 0.16
138.5 Mb
139 Mb
139.5 Mb
140 Mb
position on chr 9
EXD3
SSNA1
ANAPC2
DPP7
MAN1B1
NPDC1
ABCA2
INPP5E
SDCCAG3
SNAPC4
CARD9
DNLZ
GPSM1
QSOX2
C9orf69
p-value type
single gene test
multiple regression

**b**

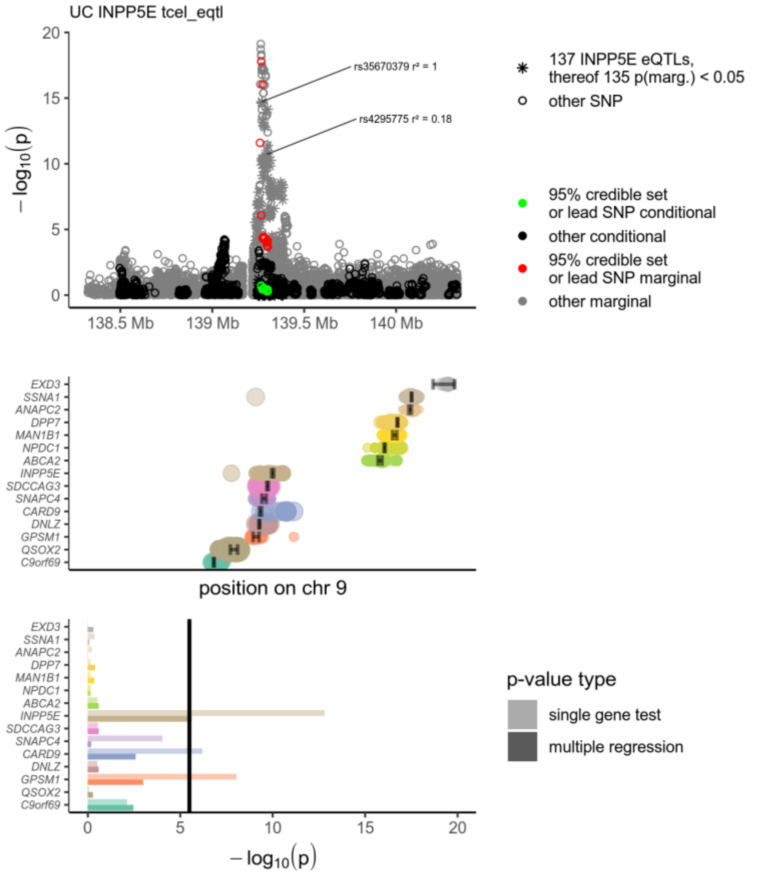
UC INPP5E tcel_eqtl
137 INPP5E eQTLs, thereof 135 p(marg.) < 0.05
other SNP
95% credible set or lead SNP conditional
other conditional
95% credible set or lead SNP marginal
other marginal
rs35670379 r² = 1
rs4295775 r² = 0.18
138.5 Mb
139 Mb
139.5 Mb
140 Mb
position on chr 9
EXD3
SSNA1
ANAPC2
DPP7
MAN1B1
NPDC1
ABCA2
INPP5E
SDCCAG3
SNAPC4
CARD9
DNLZ
GPSM1
QSOX2
C9orf69
p-value type
single gene test
multiple regression

**Supplementary Figure 7. As a negative control and to demonstrate the utility of conditioning on eQTLs, we analyzed *NOD2*, a locus with well-studied coding variants in CD known to be causal for this association.** The strong GWAS signal in the *NOD2* locus in CD was attributed to a small set of coding variants of *NOD2*[3]. By showing that in such a situation COJO cannot fully explain the signal with the eQTLs of *NOD2,* which assumes that the lead SNP retains most of its signal despite multiple eQTL signals in close proximity, we demonstrate that COJO conditioning can indicate a likely non-eQTL component of the genetic signal.

In the top panel, the GWAS summary statistics are shown in grey with and the conditioned results in black. The color is used to highlight the 95% credible set from Huang *et al.*[3]. Asterisks denote eQTLs of the gene in focus (*NOD2*). From these, a set of independent variants was selected using COJO -select. The selected variants are annotated with an $r^2$ LD value relative to the most significant eQTL variant in GWAS. In the middle, the eQTLs of the tissue and the other genes in the locus are marked. In the lower panel, the *P*-values of the single-gene UTMOST test ($\text{Single-tissue}_{\text{unconditional}}$) and the multiple gene regression approach ($\text{Single-tissue}_{\text{conditional}}$) of the genes in the locus.

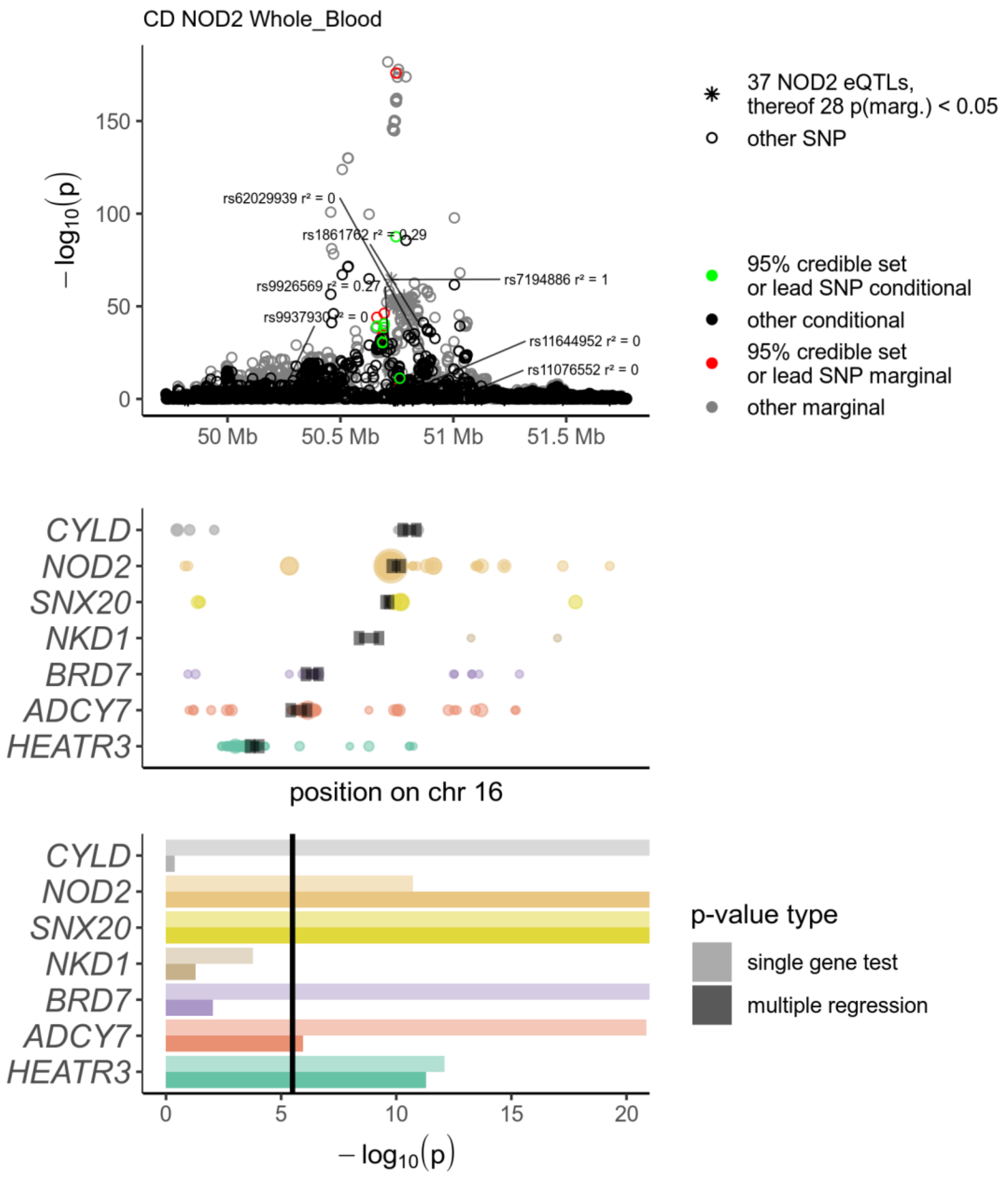
CD NOD2 Whole_Blood
− log10(p)
150
100
50
0
rs62029939 r² = 0
rs1861762 r² = 0.29
rs9926569 r² = 0.27
rs9937930 r² = 0
rs7194886 r² = 1
rs11644952 r² = 0
rs11076552 r² = 0
50 Mb
50.5 Mb
51 Mb
51.5 Mb
37 NOD2 eQTLs, thereof 28 p(marg.) < 0.05
other SNP
95% credible set or lead SNP conditional
other conditional
95% credible set or lead SNP marginal
other marginal
CYLD
NOD2
SNX20
NKD1
BRD7
ADCY7
HEATR3
position on chr 16
CYLD
NOD2
SNX20
NKD1
BRD7
ADCY7
HEATR3
0
5
10
15
20
− log10(p)
p-value type
single gene test
multiple regression

**Supplementary Figure 8. Unabridged summary plot in the style of Figure 3 for CD, *NR5A2*, Brain Hypothalamus**. This plot shows raw GWAS statistics, GWAS conditioned by eQTL SNPs of the given gene, the location and weight of the eQTLs used by the TWAS analysis, the location of the genes in the locus and their respective unconditioned and conditioned p-values. For a detailed explanation of the plot see **Supplementary Note 2**.

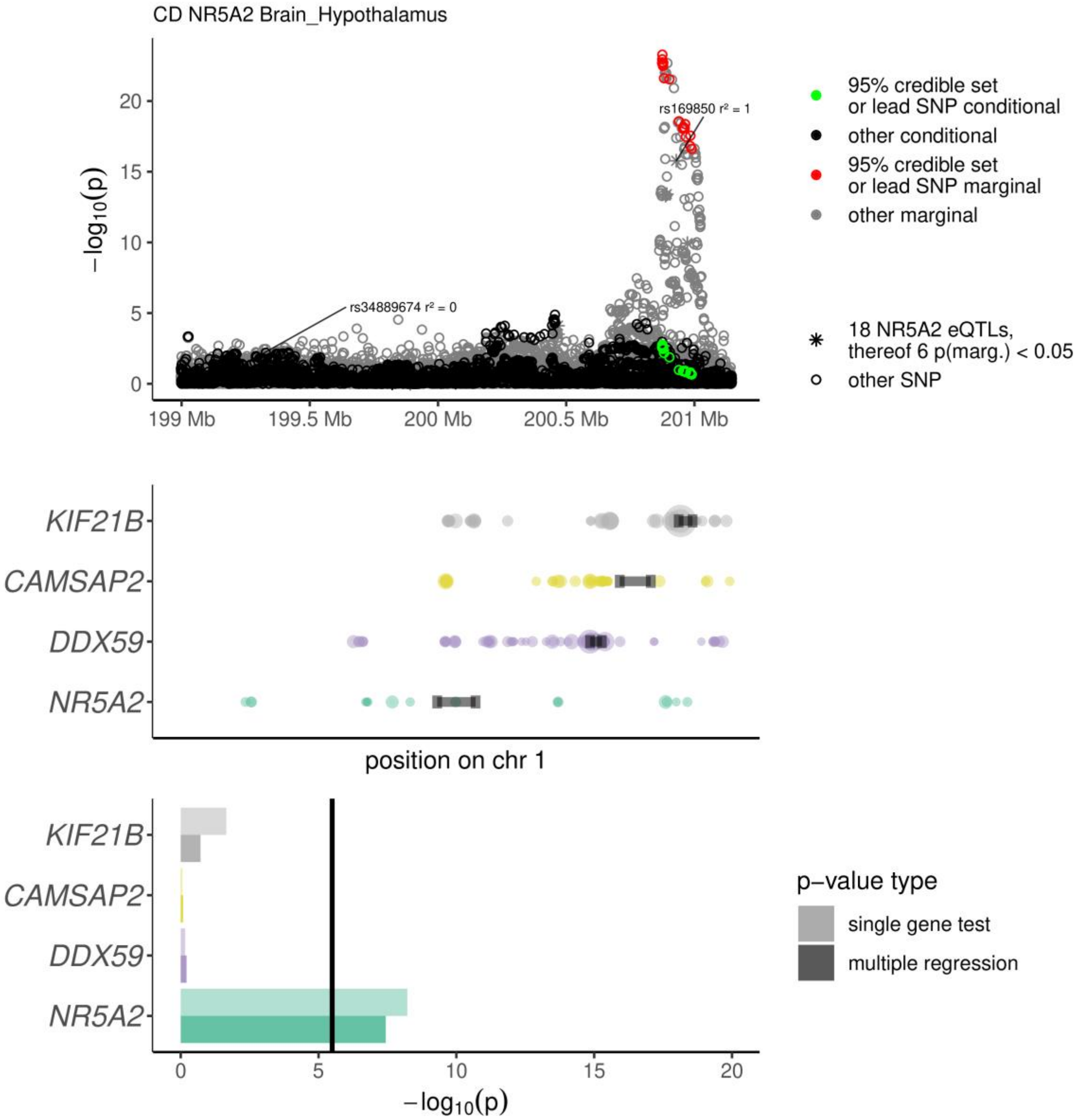

**Supplementary Figure 9. Unabridged summary plot in the style of Figure 3 for CD, *NR5A2*, Colon Transverse**. This plot shows raw GWAS statistics, GWAS conditioned by eQTL SNPs of the given gene, the location and weight of the eQTLs used by the TWAS analysis, the location of the genes in the locus and their respective unconditioned and conditioned p-values. For a detailed explanation of the plot see **Supplementary Note 2**.

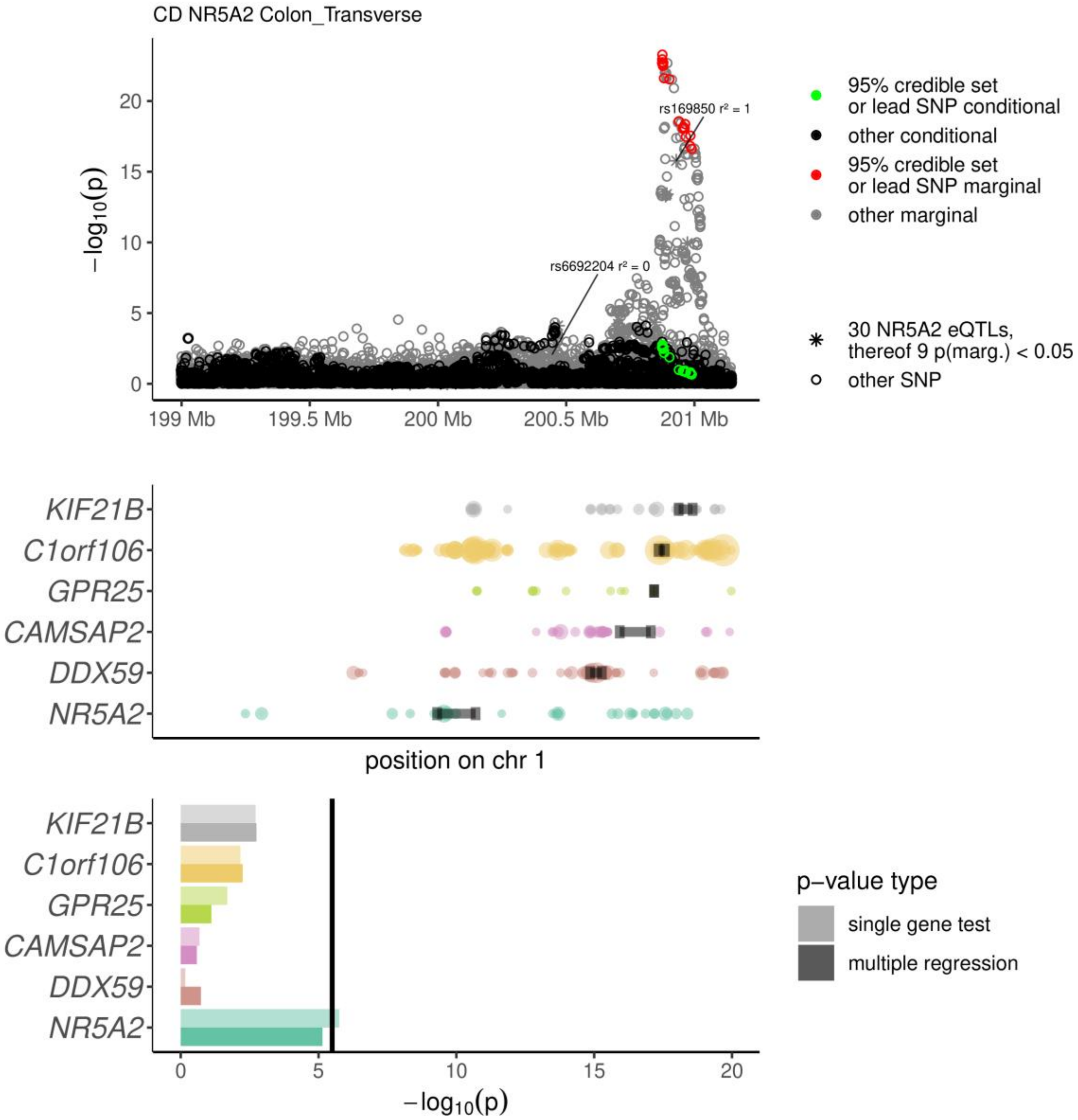

**Supplementary Figure 10. Unabridged summary plot in the style of Figure 3 for CD, *PPP3CA*, Brain Putamen basal ganglia.** This plot shows raw GWAS statistics, GWAS conditioned by eQTL SNPs of the given gene, the location and weight of the eQTLs used by the TWAS analysis, the location of the genes in the locus and their respective unconditioned and conditioned p-values. For a detailed explanation of the plot see **Supplementary Note 2**.

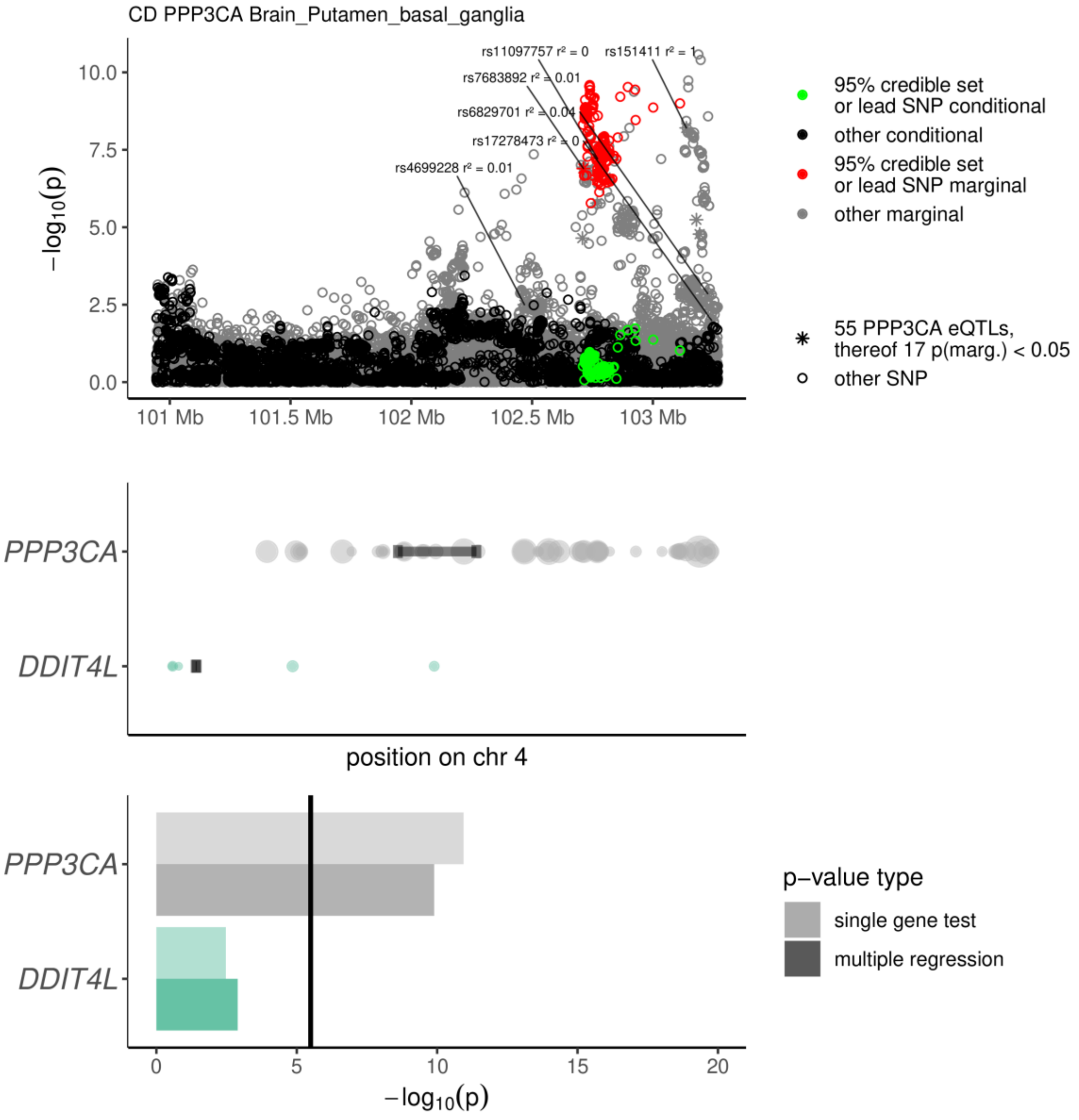

**Supplementary Figure 11. Unabridged summary plot in the style of Figure 3 for CD, *PPP3CA*, Colon Transverse**. This plot shows raw GWAS statistics, conditioned by eQTL SNPs of the given gene, the location and weight of the eQTLs used by the TWAS analysis, the location of the genes in the locus and their respective unconditioned and conditioned p-values. For a detailed explanation of the plot see **Supplementary Note 2**.

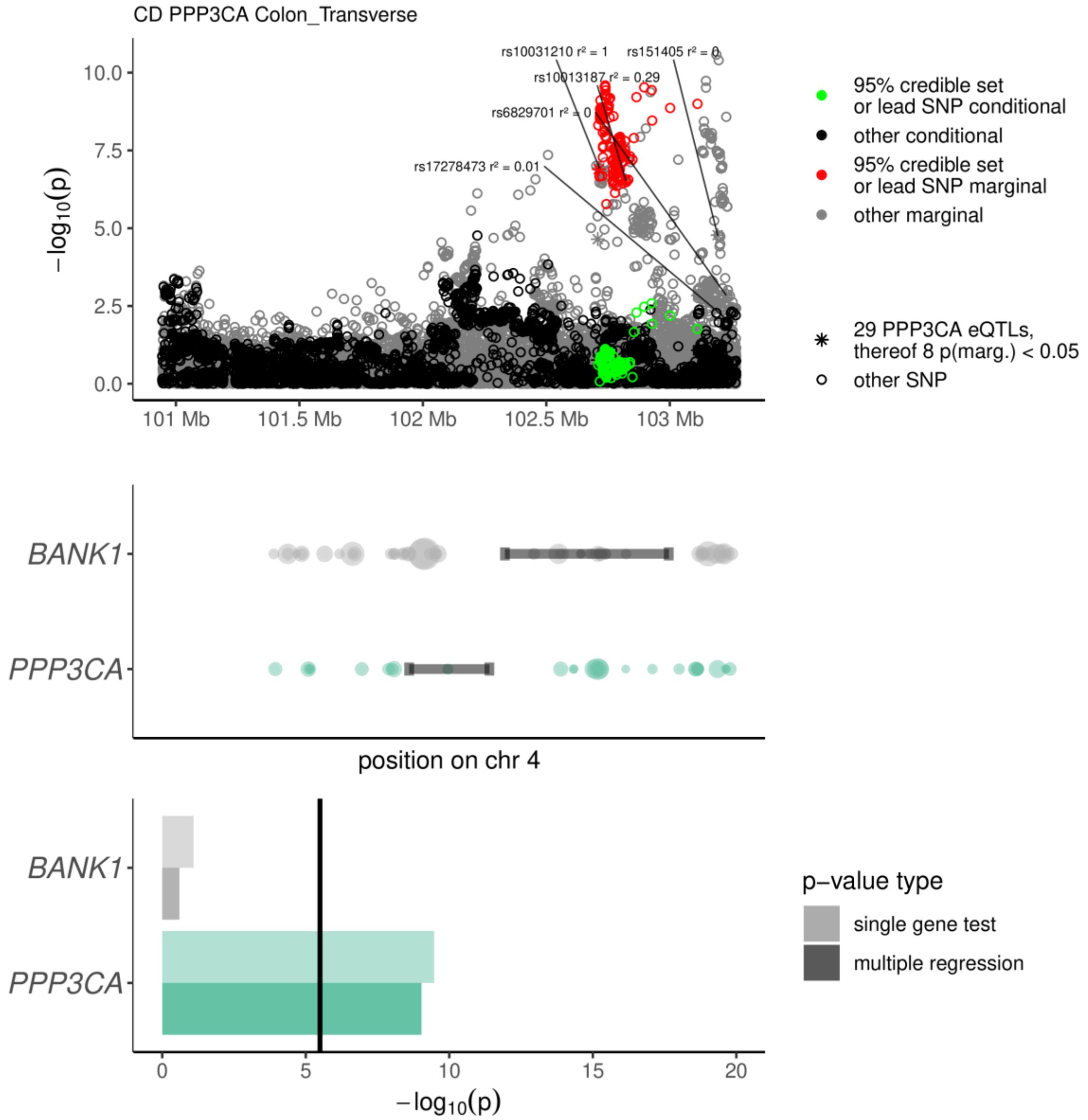

**Supplementary Figure 12. Unabridged summary plot in the style of Figure 3 for CD, *SF3B1*, Brain Nucleus accumbens basal ganglia**. This plot shows raw GWAS statistics, GWAS conditioned by eQTL SNPs of the given gene, the location and weight of the eQTLs used by the TWAS analysis, the location of the genes in the locus and their respective unconditioned and conditioned p-values. For a detailed explanation of the plot see **Supplementary Note 2**.

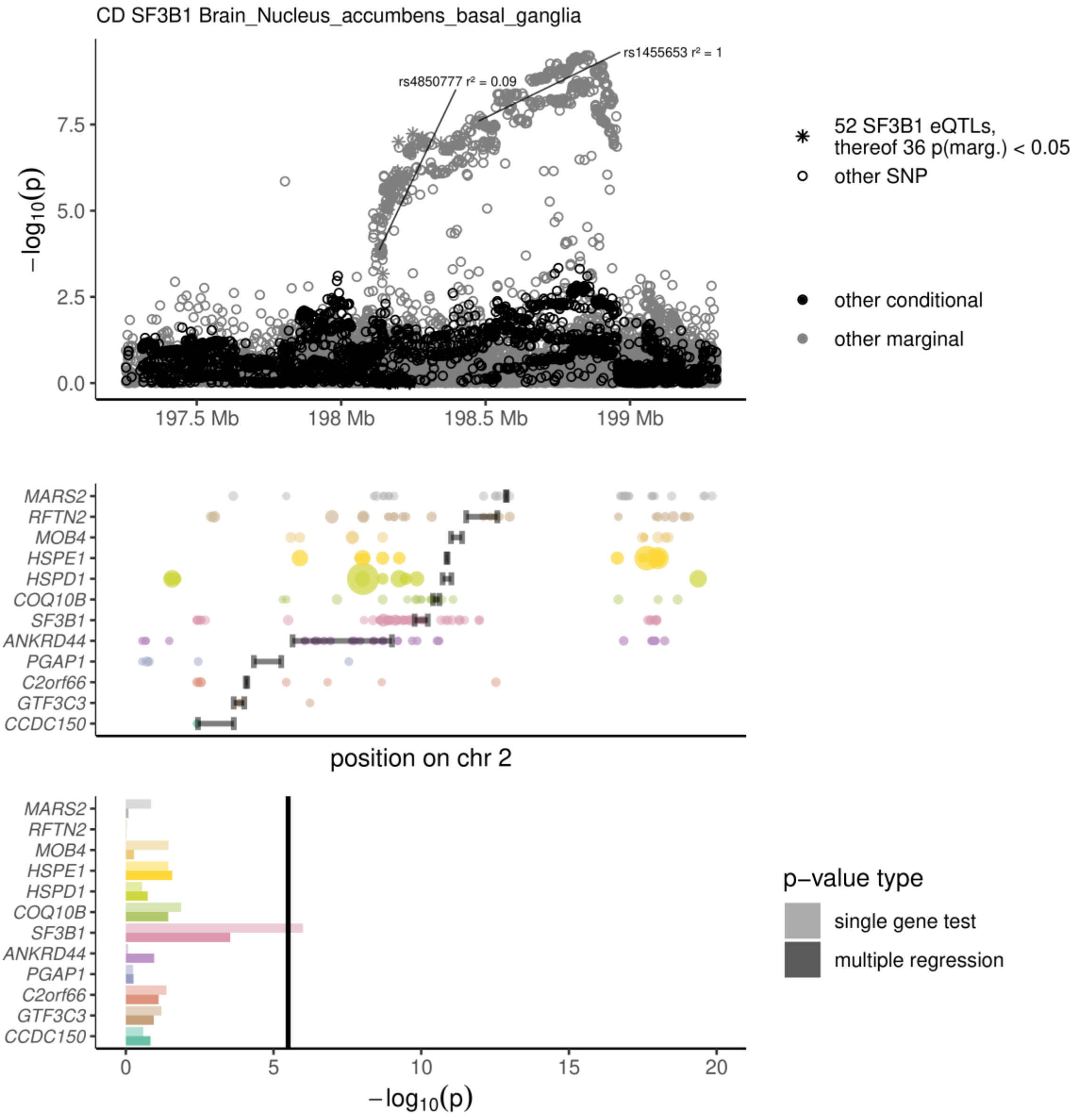

**Supplementary Figure 13. Unabridged summary plot in the style of Figure 3 for CD, *SF3B1*, Colon Sigmoid**. This plot shows raw GWAS statistics, GWAS conditioned by eQTL SNPs of the given gene, the location and weight of the eQTLs used by the TWAS analysis, the location of the genes in the locus and their respective unconditioned and conditioned p-values. For a detailed explanation of the plot see **Supplementary Note 2**.

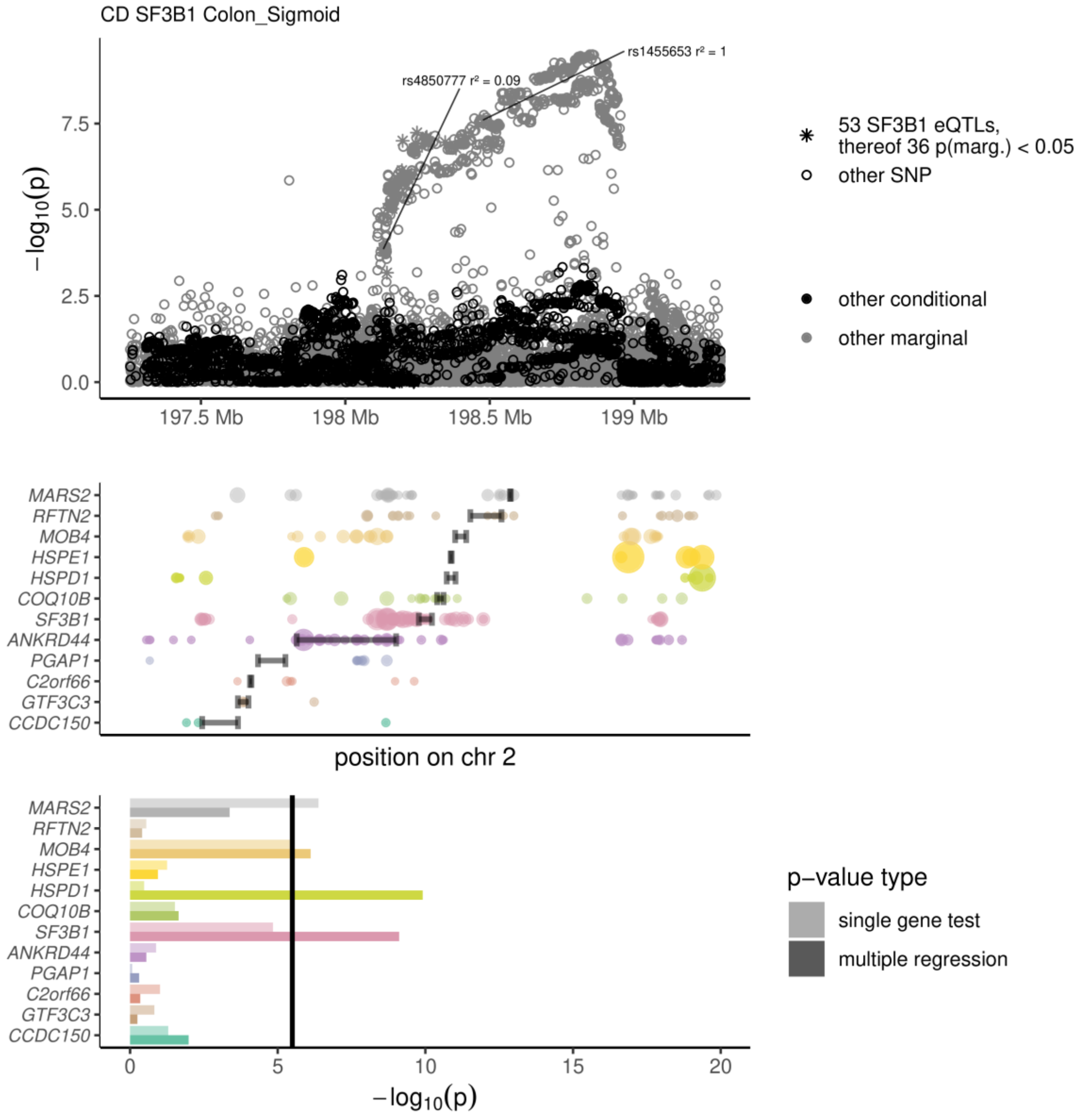

**Supplementary Figure 14. Unabridged summary plot in the style of Figure 3 for CD, *SGSM3*, Colon Sigmoid**. This plot shows raw GWAS statistics, GWAS conditioned by eQTL SNPs of the given gene, the location and weight of the eQTLs used by the TWAS analysis, the location of the genes in the locus and their respective unconditioned and conditioned p-values. For a detailed explanation of the plot see **Supplementary Note 2**.

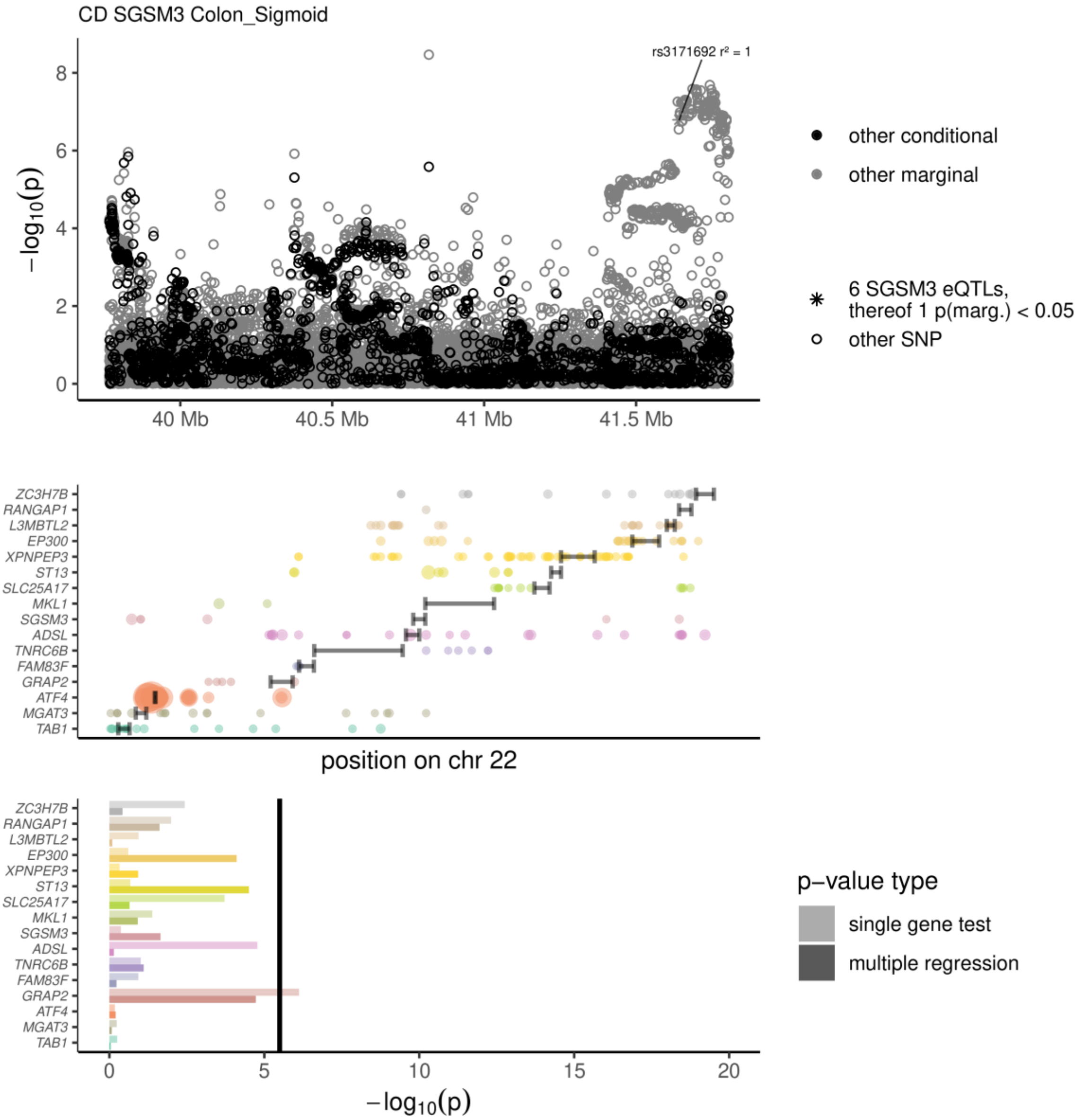

**Supplementary Figure 15. Unabridged summary plot in the style of Figure 3 for CD, *SGSM3*, Colon Transverse**. This plot shows raw GWAS statistics, GWAS conditioned by eQTL SNPs of the given gene, the location and weight of the eQTLs used by the TWAS analysis, the location of the genes in the locus and their respective unconditioned and conditioned p-values. For a detailed explanation of the plot see **Supplementary Note 2**.

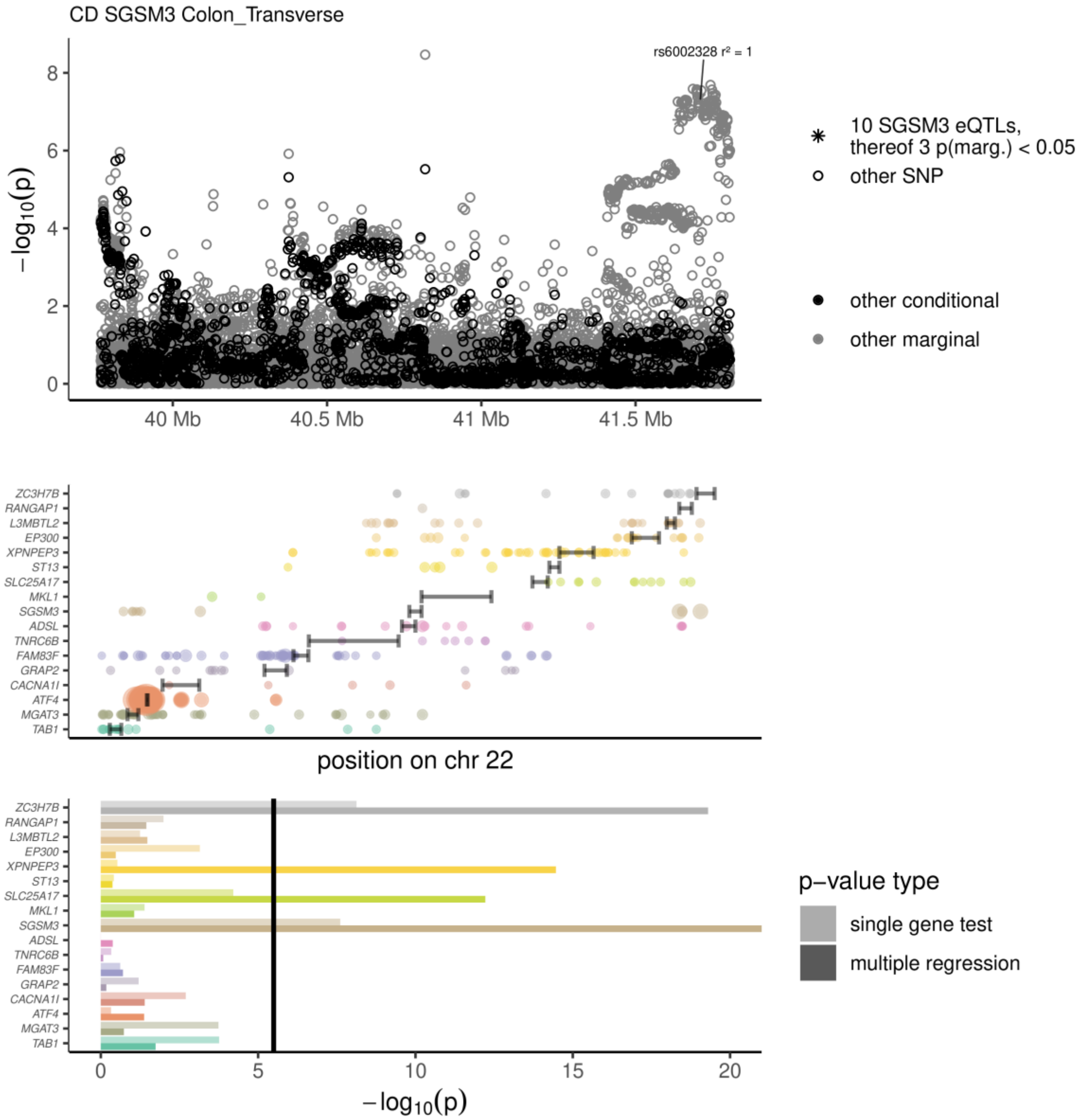

**Supplementary Figure 16. Unabridged summary plot in the style of Figure 3 for CD, *ZC3H7B*, Colon Transverse**. This plot shows raw GWAS statistics, GWAS conditioned by eQTL SNPs of the given gene, the location and weight of the eQTLs used by the TWAS analysis, the location of the genes in the locus and their respective unconditioned and conditioned p-values. For a detailed explanation of the plot see **Supplementary Note 2**.

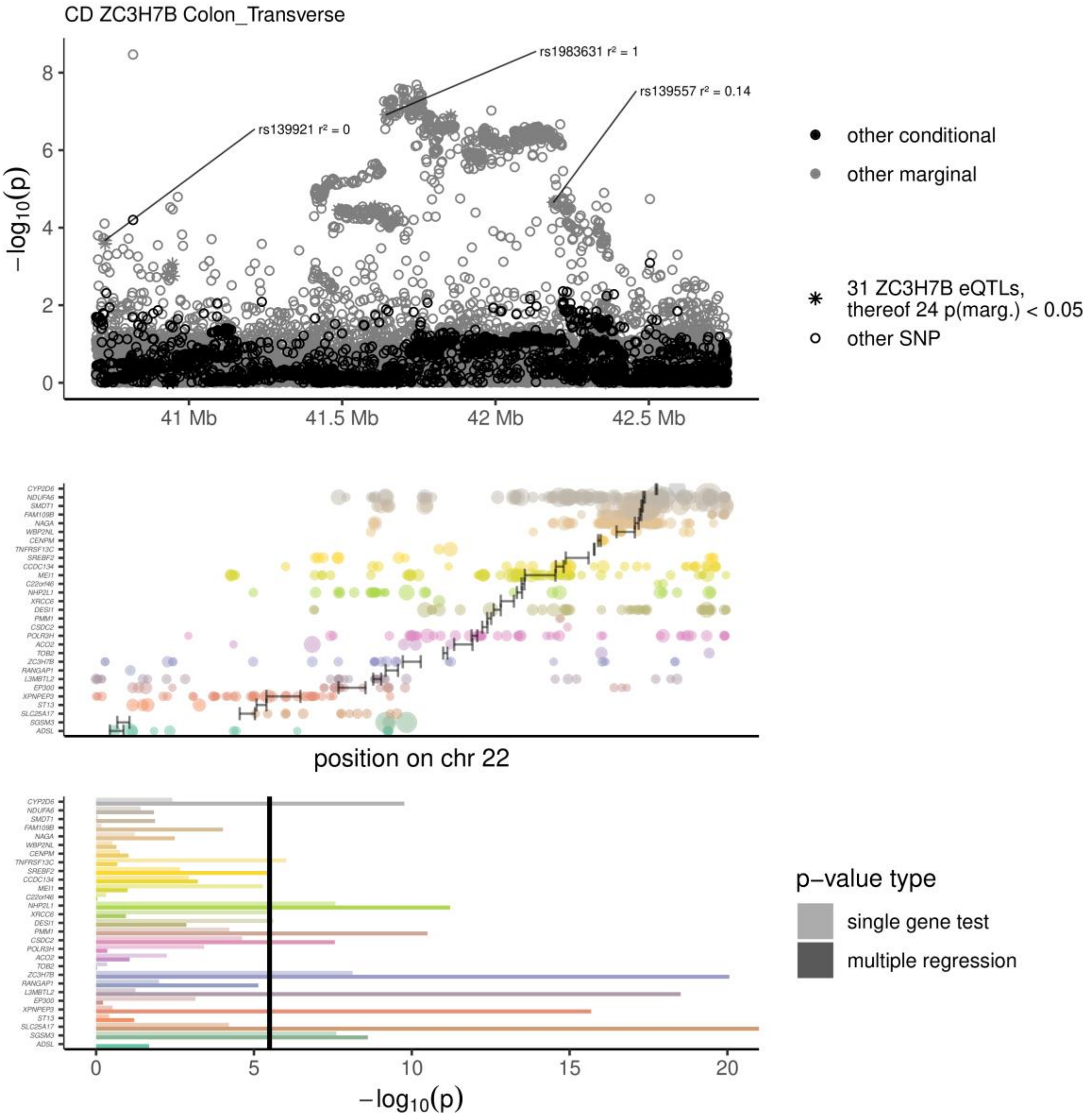

**Supplementary Figure 17. Unabridged summary plot in the style of Figure 3 for CD, *ZC3H7B*, Esophagus Muscularis**. This plot shows raw GWAS statistics, GWAS conditioned by eQTL SNPs of the given gene, the location and weight of the eQTLs used by the TWAS analysis, the location of the genes in the locus and their respective unconditioned and conditioned p-values. For a detailed explanation of the plot see **Supplementary Note 2**.

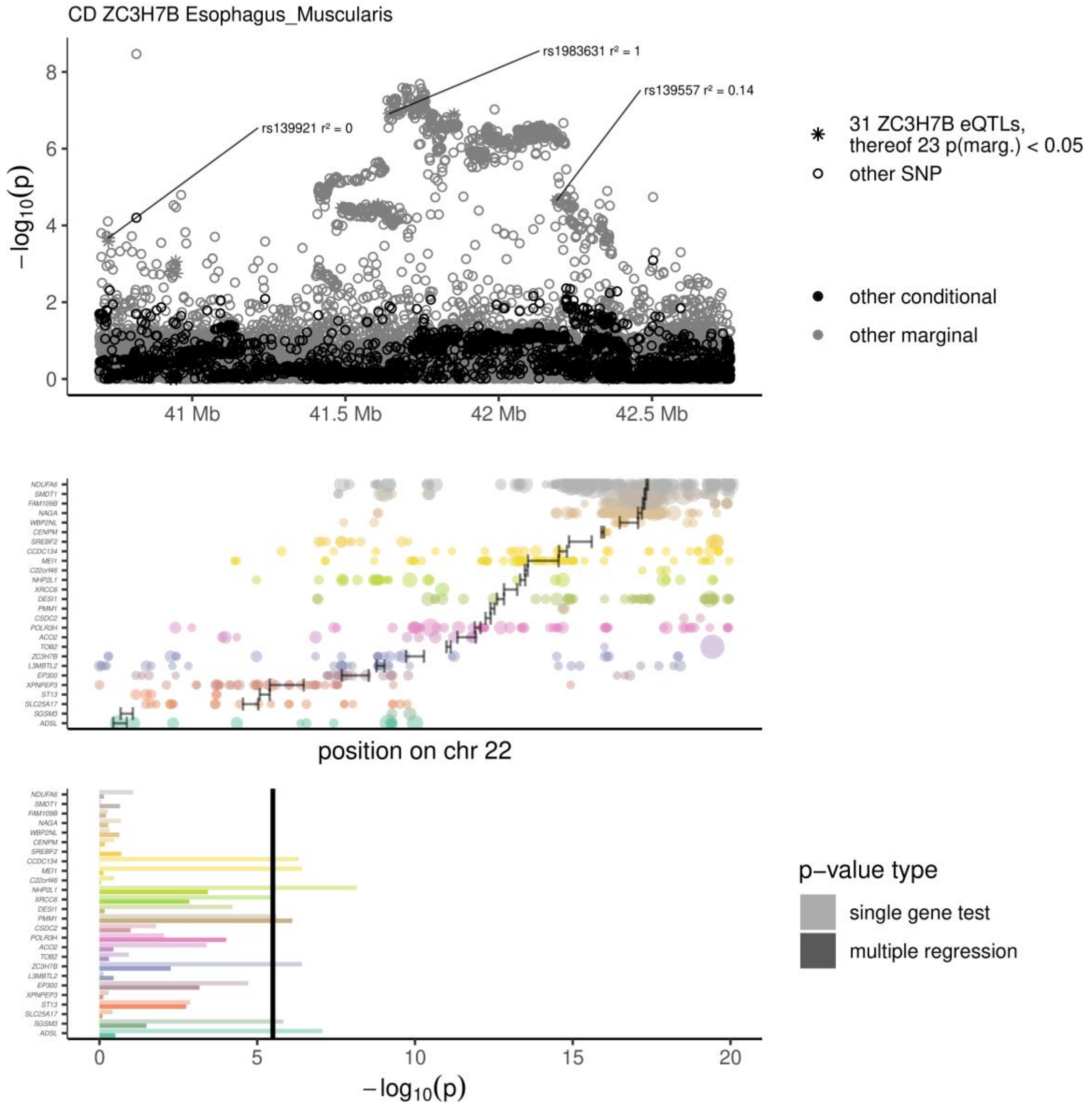

**Supplementary Figure 18. Unabridged summary plot in the style of Figure 3 for SCZ, *INO80E*, Brain Hippocampus**. This plot shows raw GWAS statistics, GWAS conditioned by eQTL SNPs of the given gene, the location and weight of the eQTLs used by the TWAS analysis, the location of the genes in the locus and their respective unconditioned and conditioned p-values. For a detailed explanation of the plot see **Supplementary Note 2**.

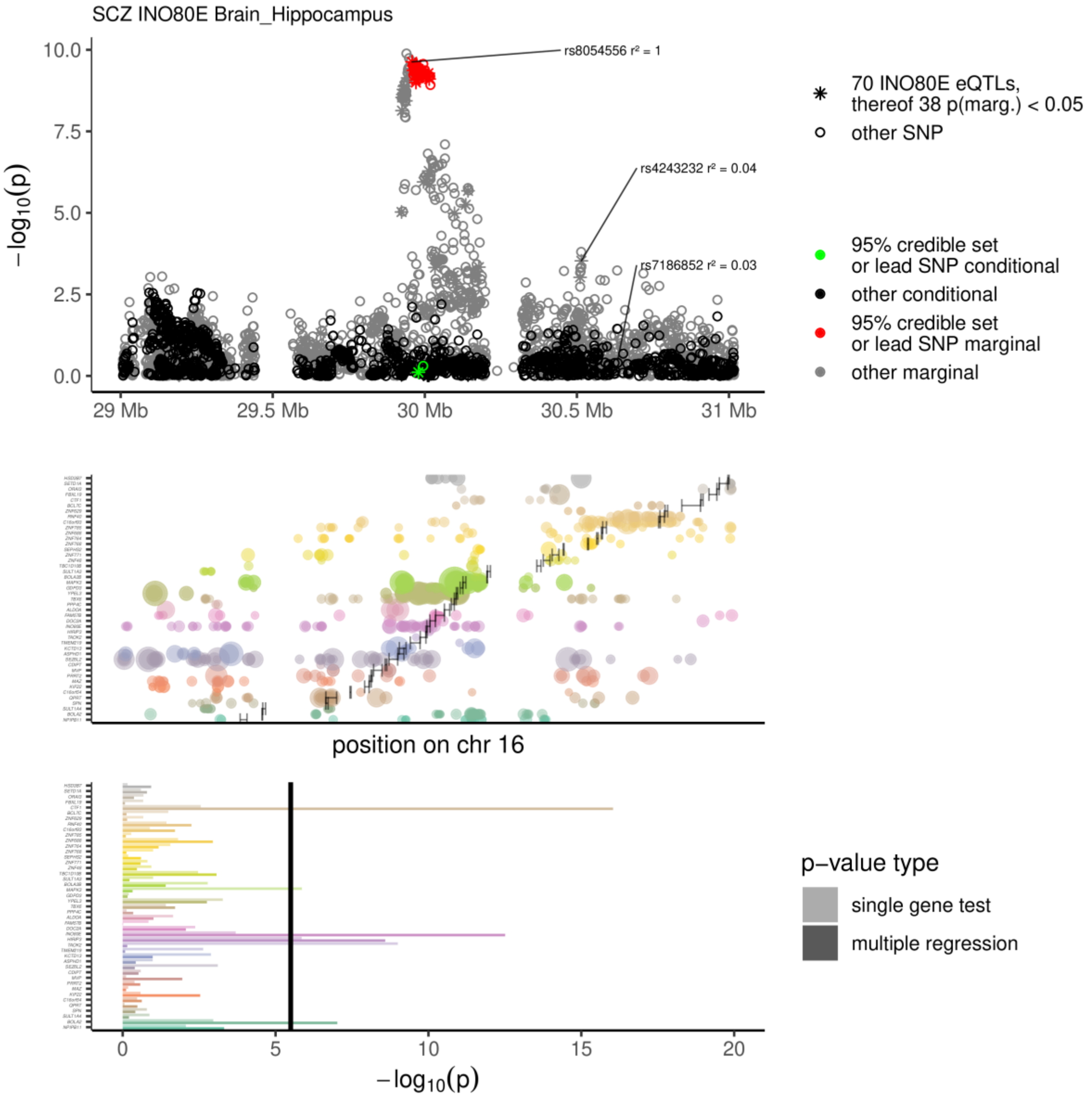

**Supplementary Figure 19. Unabridged summary plot in the style of Figure 3 for SCZ, *INO80E*, Colon Transverse**. This plot shows raw GWAS statistics, conditioned by eQTL SNPs of the given gene, the location and weight of the eQTLs used by the TWAS analysis, the location of the genes in the locus and their respective unconditioned and conditioned p-values. For a detailed explanation of the plot see **Supplementary Note 2**.

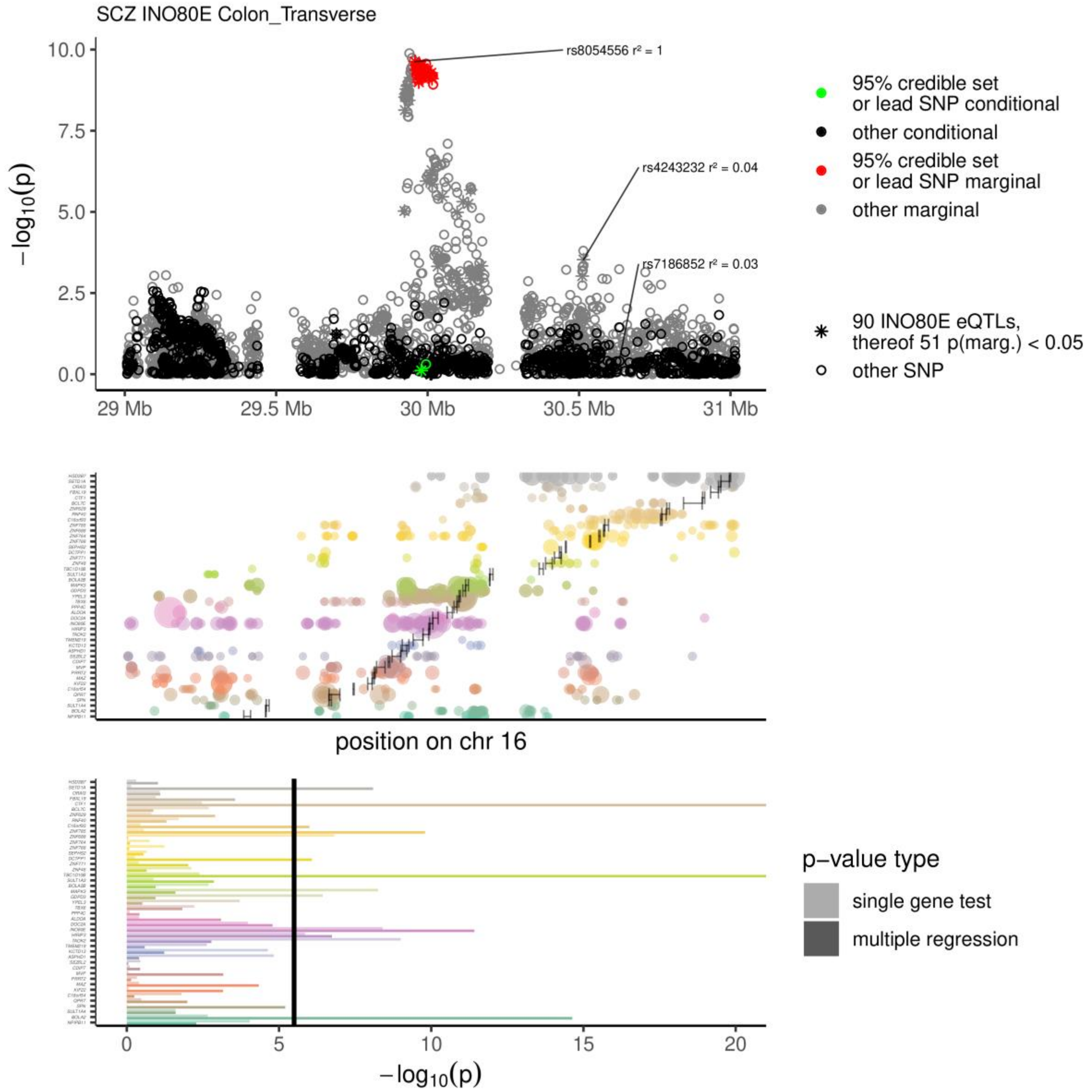

**Supplementary Figure 20. Unabridged summary plot in the style of Figure 3 for SCZ, *NR5A2*, Brain Hypothalamus**. This plot shows raw GWAS statistics, GWAS conditioned by eQTL SNPs of the given gene, the location and weight of the eQTLs used by the TWAS analysis, the location of the genes in the locus and their respective unconditioned and conditioned p-values. For a detailed explanation of the plot see **Supplementary Note 2**.

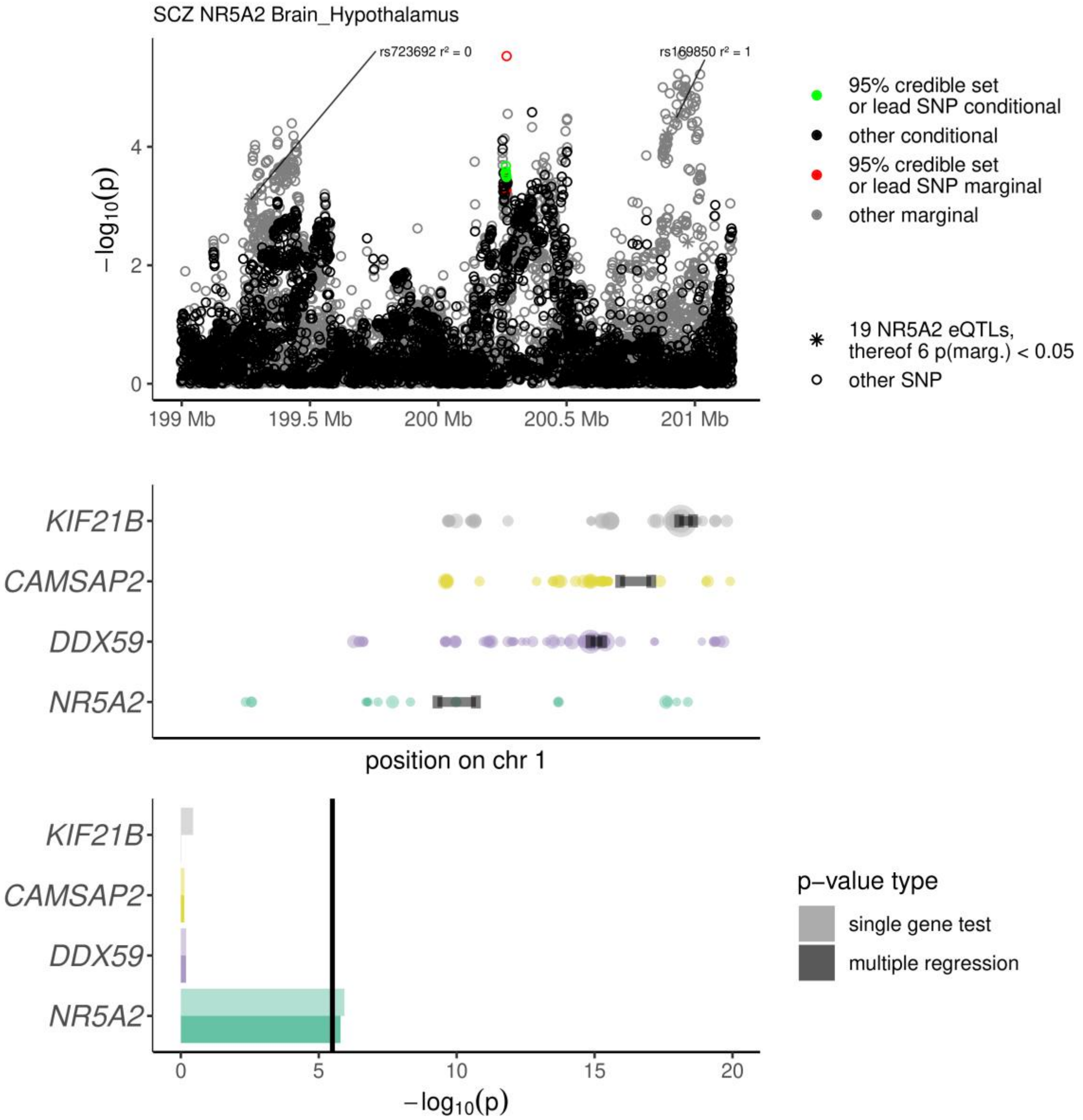

**Supplementary Figure 21. Unabridged summary plot in the style of Figure 3 for SCZ, *NR5A2*, Colon Transverse**. This plot shows raw GWAS statistics, GWAS conditioned by eQTL SNPs of the given gene, the location and weight of the eQTLs used by the TWAS analysis, the location of the genes in the locus and their respective unconditioned and conditioned p-values. For a detailed explanation of the plot see **Supplementary Note 2**.

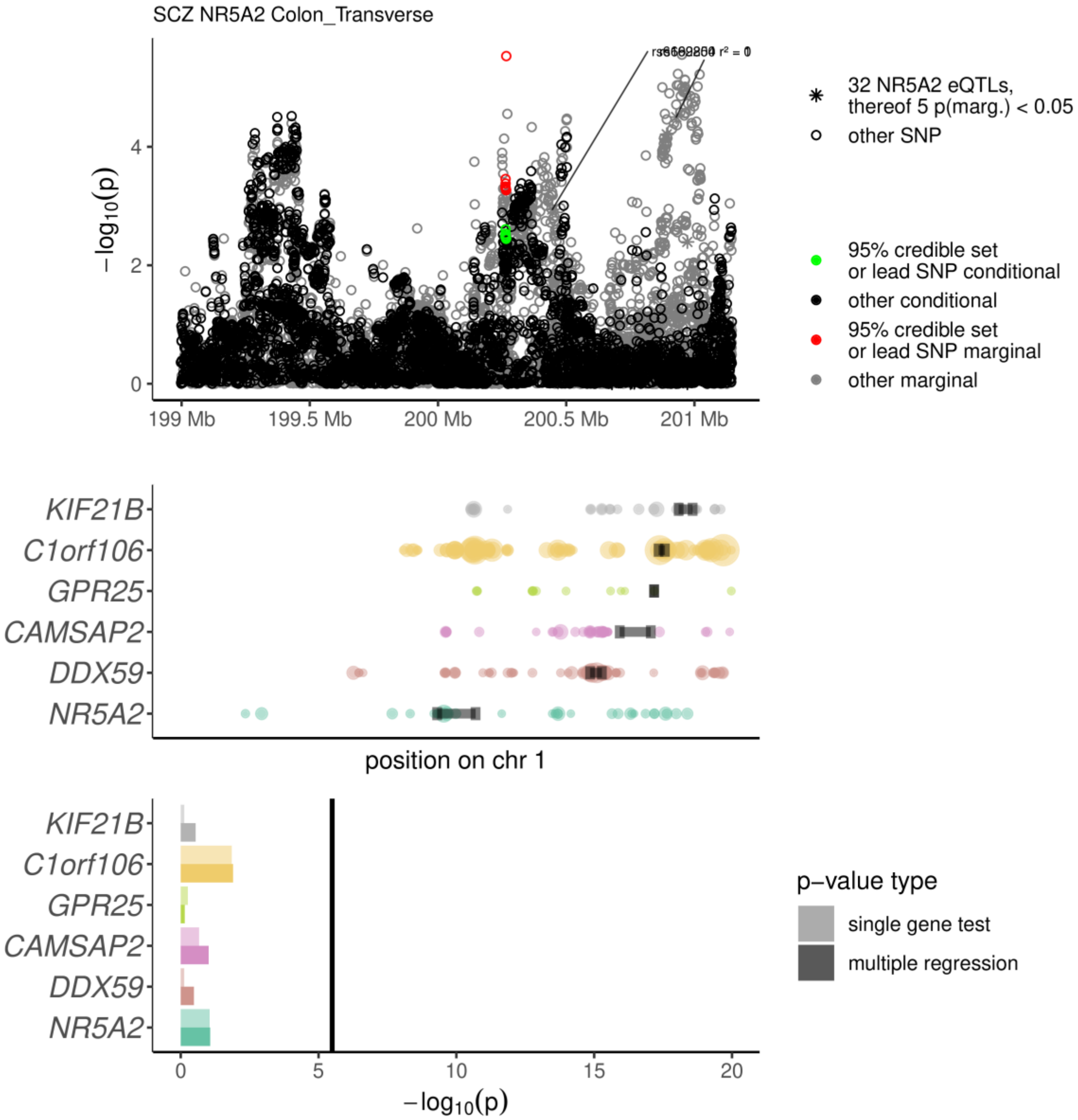

**Supplementary Figure 22. Unabridged summary plot in the style of Figure 3 for SCZ, *PPP3CA*, Brain Putamen basal ganglia**. This plot shows raw GWAS statistics, GWAS conditioned by eQTL SNPs of the given gene, the location and weight of the eQTLs used by the TWAS analysis, the location of the genes in the locus and their respective unconditioned and conditioned p-values. For a detailed explanation of the plot see **Supplementary Note 2**.

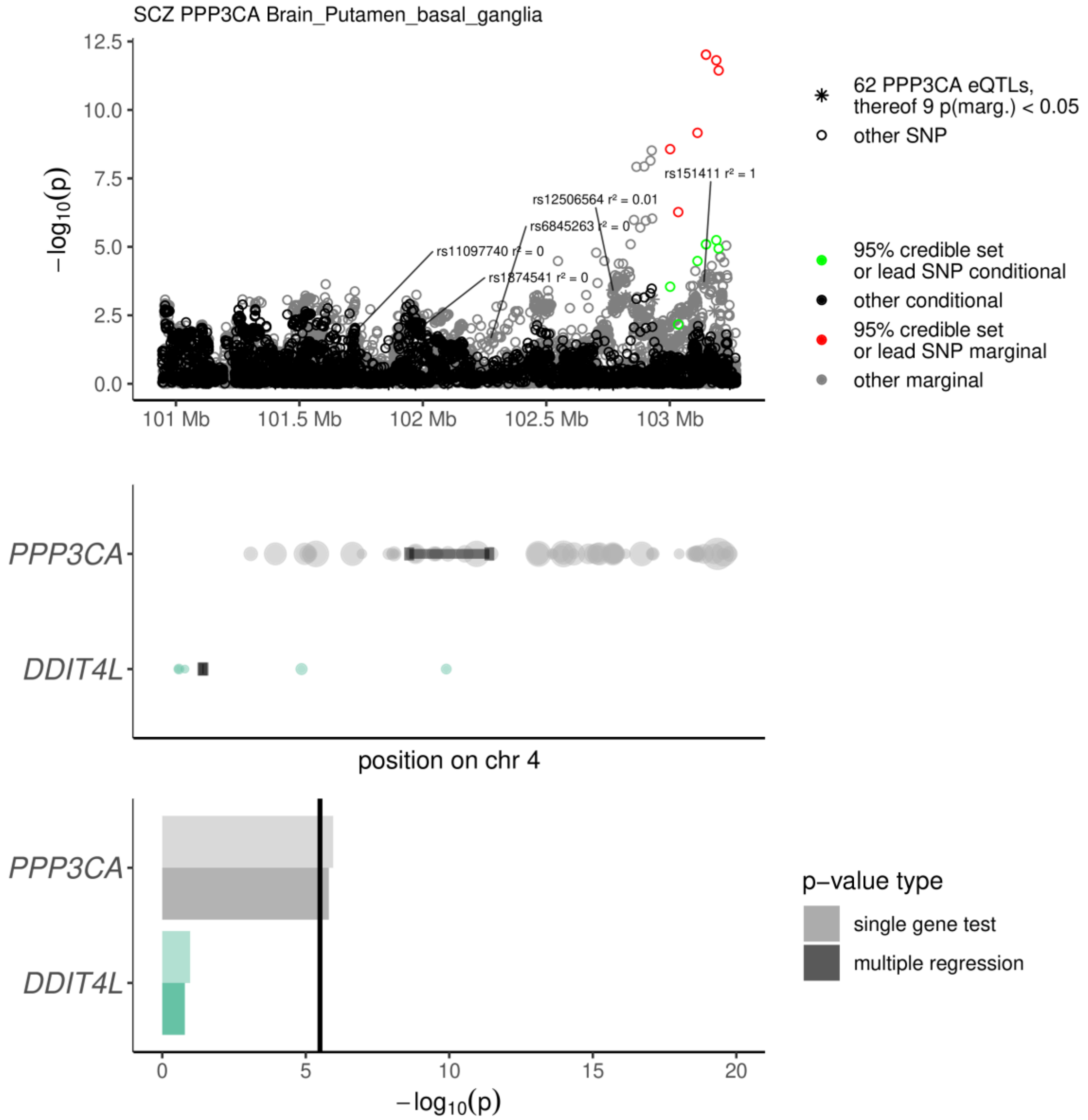

**Supplementary Figure 23. Unabridged summary plot in the style of Figure 3 for SCZ, *PPP3CA*, Colon Transverse**. This plot shows raw GWAS statistics, GWAS conditioned by eQTL SNPs of the given gene, the location and weight of the eQTLs used by the TWAS analysis, the location of the genes in the locus and their respective unconditioned and conditioned p-values. For a detailed explanation of the plot see **Supplementary Note 2**.

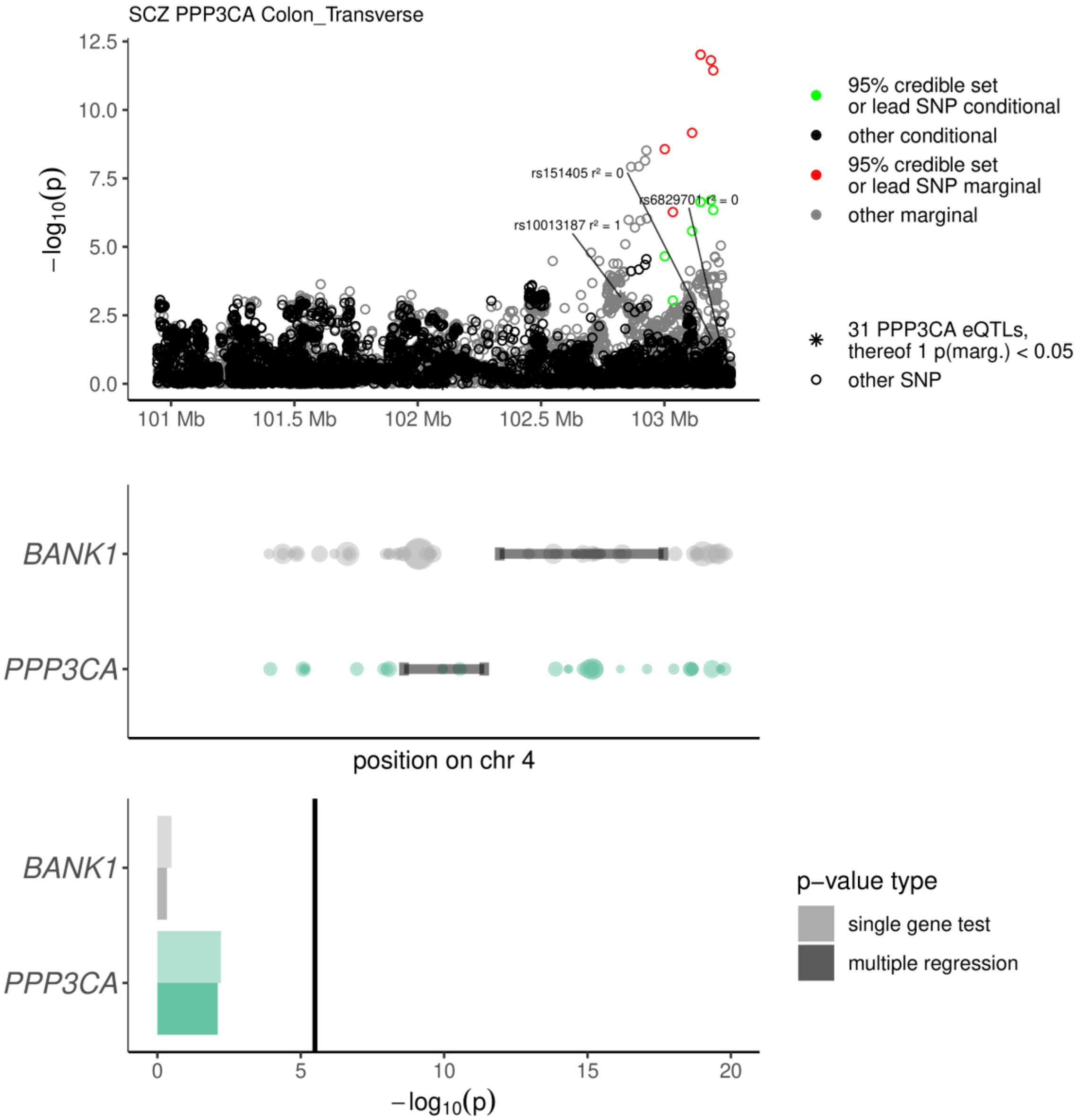

**Supplementary Figure 24. Unabridged summary plot in the style of Figure 3 for SCZ, *SATB2*, Brain Frontal Cortex BA9**. This plot shows raw GWAS statistics, GWAS conditioned by eQTL SNPs of the given gene, the location and weight of the eQTLs used by the TWAS analysis, the location of the genes in the locus and their respective unconditioned and conditioned p-values. For a detailed explanation of the plot see **Supplementary Note 2**.

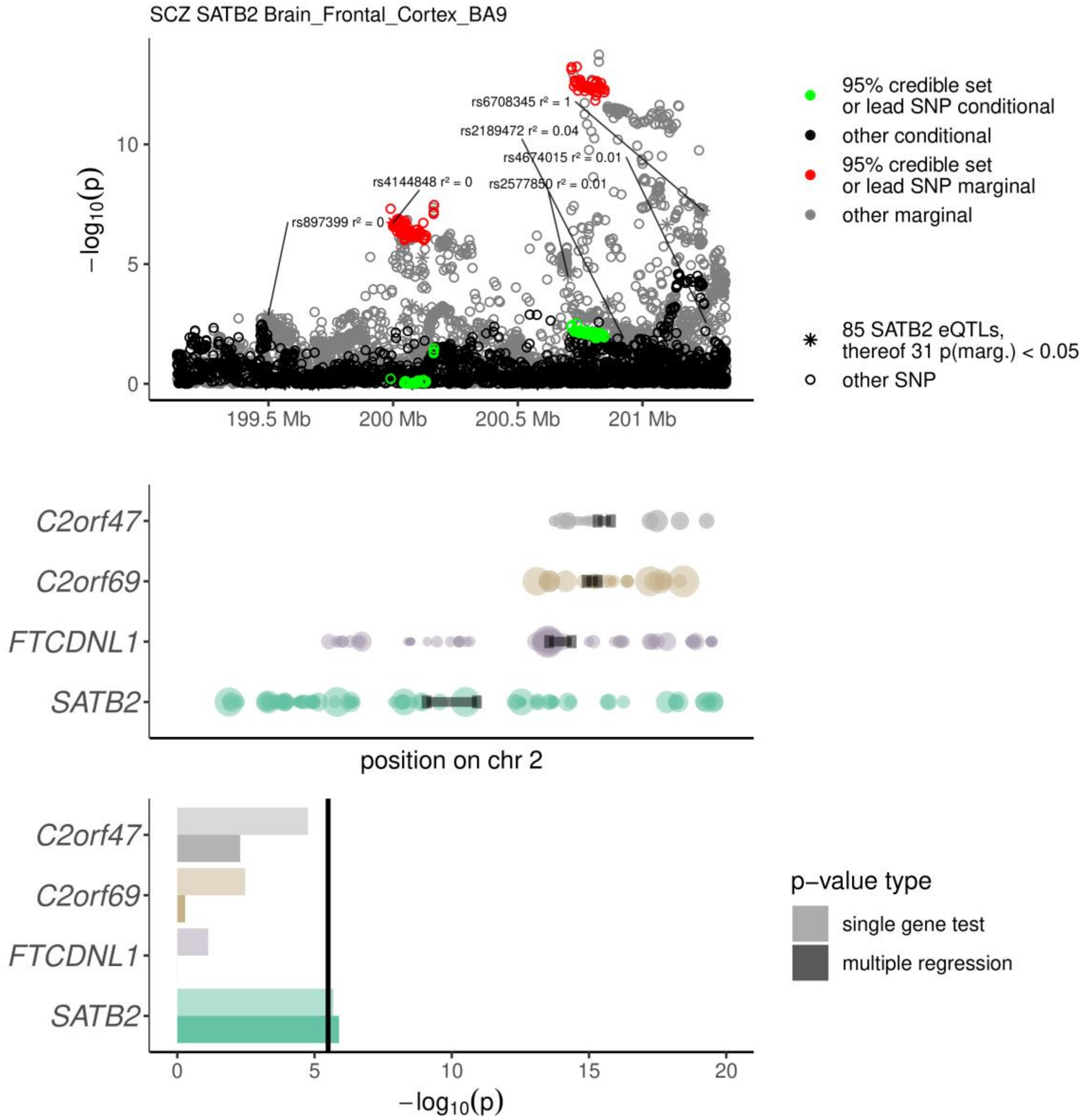

**Supplementary Figure 25. Unabridged summary plot in the style of Figure 3 for SCZ, *SATB2*, Colon Sigmoid**. This plot shows raw GWAS statistics, GWAS conditioned by eQTL SNPs of the given gene, the location and weight of the eQTLs used by the TWAS analysis, the location of the genes in the locus and their respective unconditioned and conditioned p-values. For a detailed explanation of the plot see **Supplementary Note 2**.

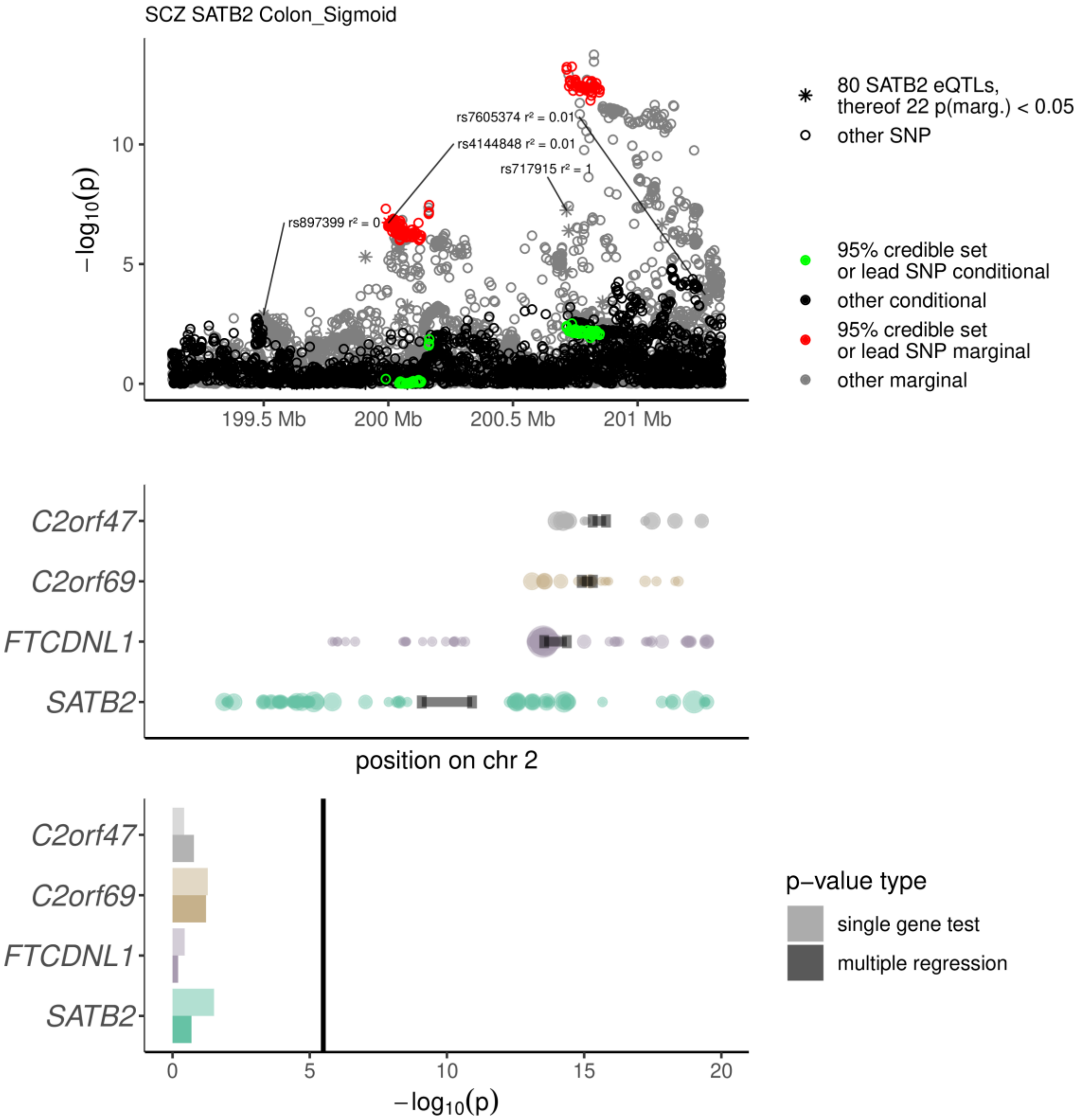

**Supplementary Figure 26. Unabridged summary plot in the style of Figure 3 for SCZ, *SF3B1*, Brain Nucleus accumbens basal ganglia**. This plot shows raw GWAS statistics, GWAS conditioned by eQTL SNPs of the given gene, the location and weight of the eQTLs used by the TWAS analysis, the location of the genes in the locus and their respective unconditioned and conditioned p-values. For a detailed explanation of the plot see **Supplementary Note 2**.

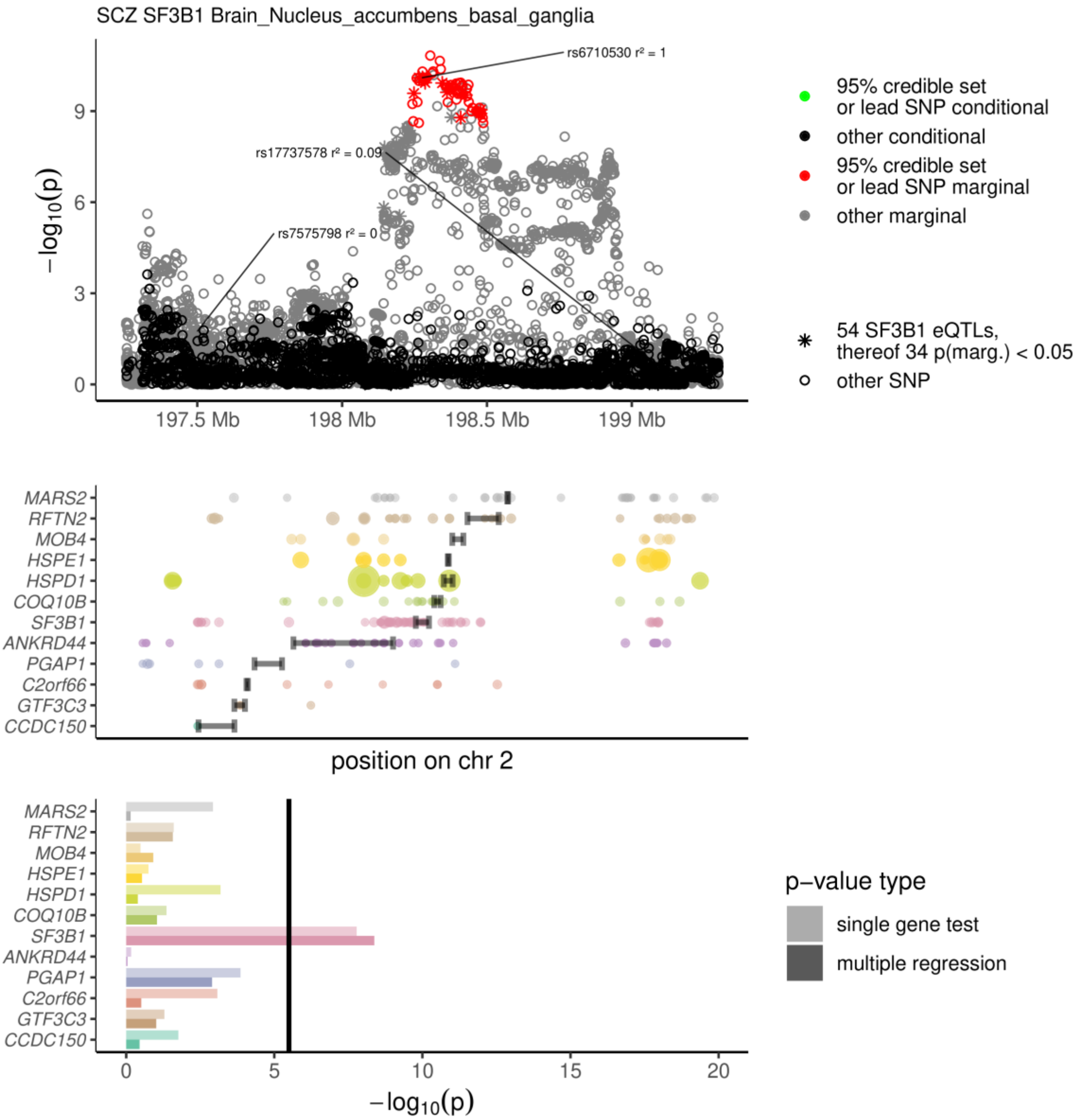

**Supplementary Figure 27. Unabridged summary plot in the style of Figure 3 for SCZ, *SF3B1*, Colon Sigmoid**. This plot shows raw GWAS statistics, conditioned by eQTL SNPs of the given gene, the location and weight of the eQTLs used by the TWAS analysis, the location of the genes in the locus and their respective unconditioned and conditioned p-values. For a detailed explanation of the plot see **Supplementary Note 2**.

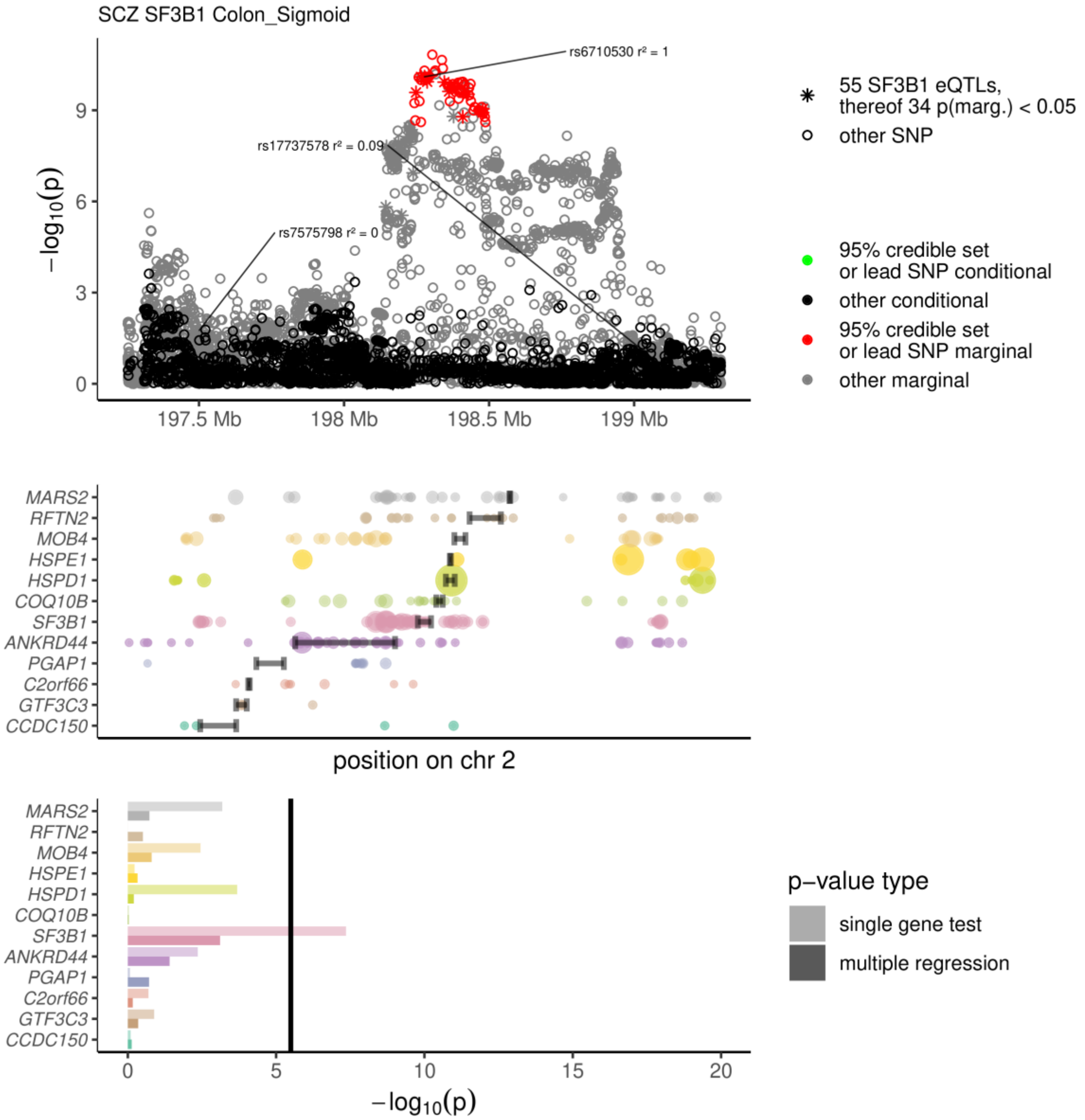

**Supplementary Figure 28. Unabridged summary plot in the style of Figure 3 for SCZ, *SGSM3*, Colon Sigmoid**. This plot shows raw GWAS statistics, GWAS conditioned by eQTL SNPs of the given gene, the location and weight of the eQTLs used by the TWAS analysis, the location of the genes in the locus and their respective unconditioned and conditioned p-values. For a detailed explanation of the plot see **Supplementary Note 2**.

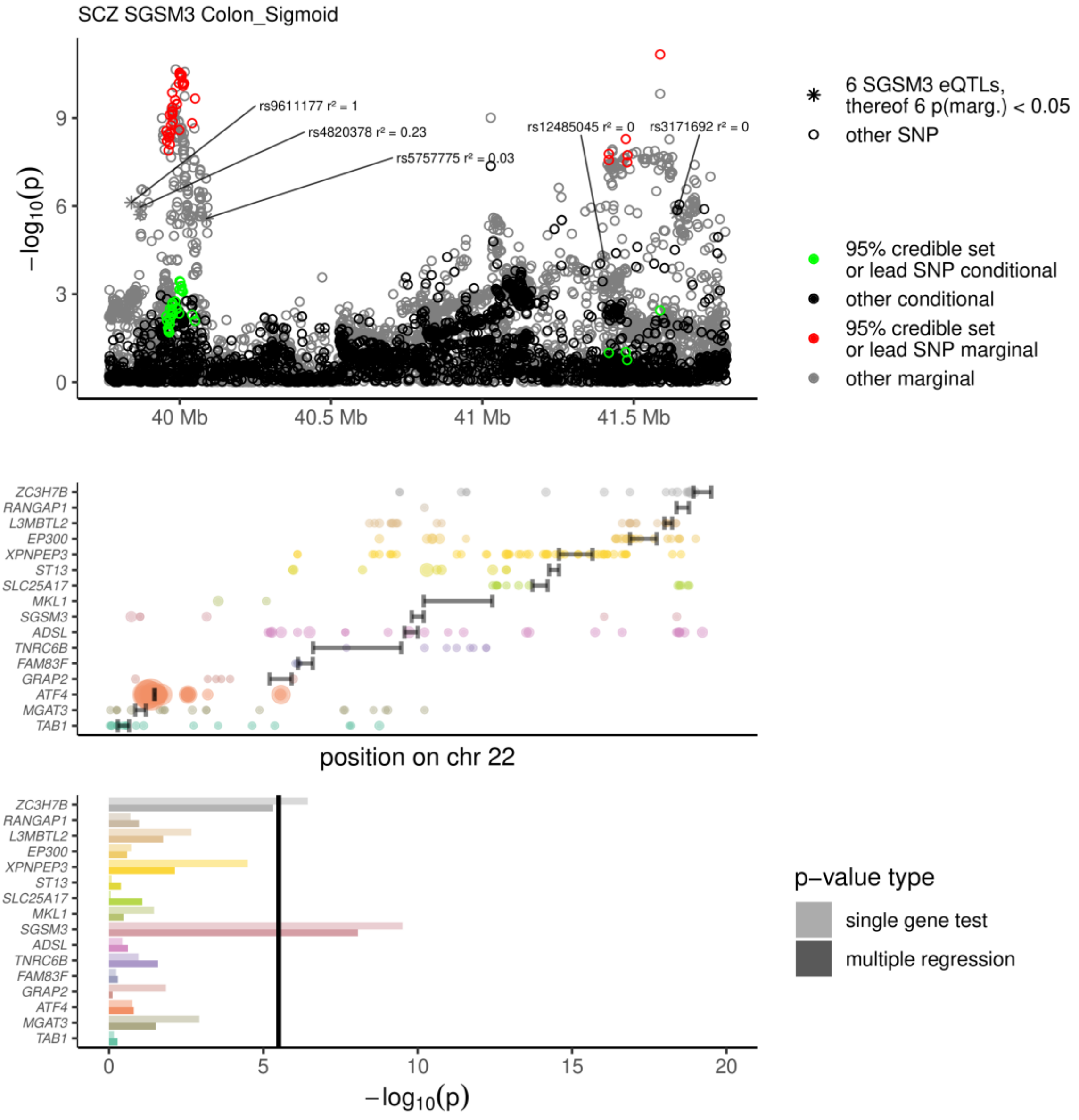

**Supplementary Figure 29. Unabridged summary plot in the style of Figure 3 for SCZ, *SGSM3*, Colon Transverse**. This plot shows raw GWAS statistics, GWAS conditioned by eQTL SNPs of the given gene, the location and weight of the eQTLs used by the TWAS analysis, the location of the genes in the locus and their respective unconditioned and conditioned p-values. For a detailed explanation of the plot see **Supplementary Note 2**.

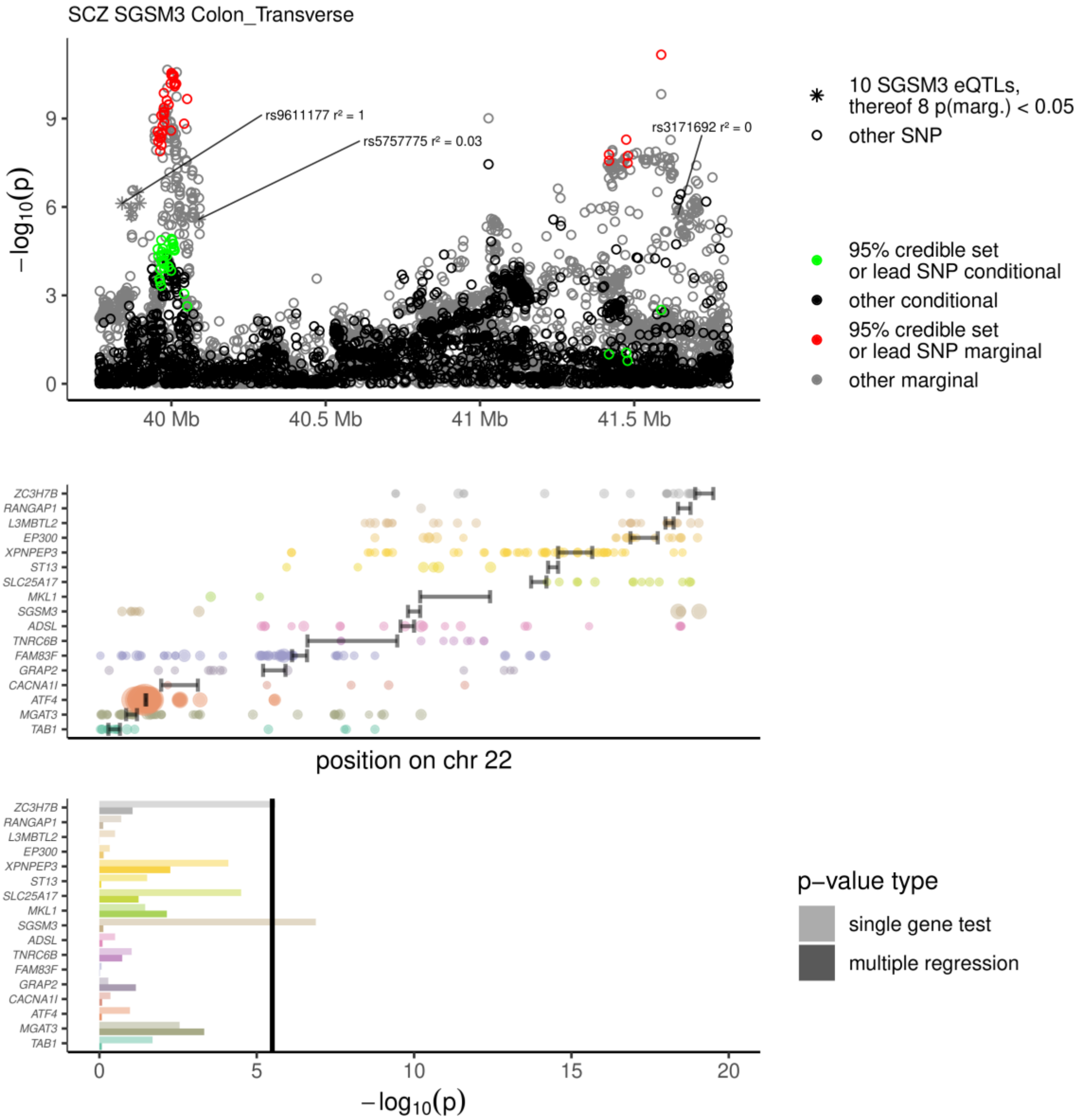

**Supplementary Figure 30. Unabridged summary plot in the style of Figure 3 for SCZ, *ZC3H7B*, Colon Transverse**. This plot shows raw GWAS statistics, GWAS conditioned by eQTL SNPs of the given gene, the location and weight of the eQTLs used by the TWAS analysis, the location of the genes in the locus and their respective unconditioned and conditioned p-values. For a detailed explanation of the plot see **Supplementary Note 2**.

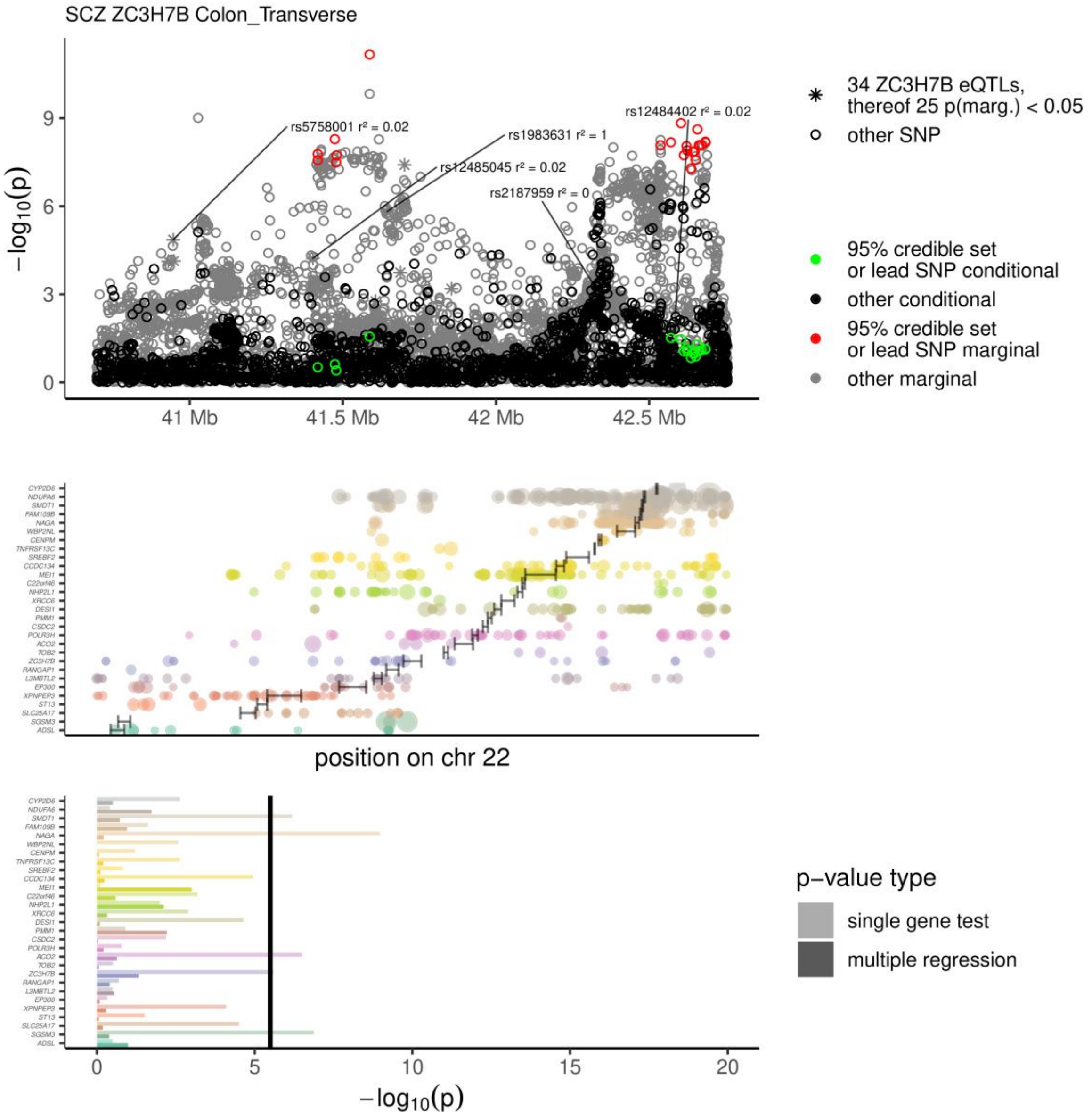

**Supplementary Figure 31. Unabridged summary plot in the style of Figure 3 for SCZ, *ZC3H7B*, Esophagus muscularis**. This plot shows raw GWAS statistics, GWAS conditioned by eQTL SNPs of the given gene, the location and weight of the eQTLs used by the TWAS analysis, the location of the genes in the locus and their respective unconditioned and conditioned p-values. For a detailed explanation of the plot see **Supplementary Note 2**.

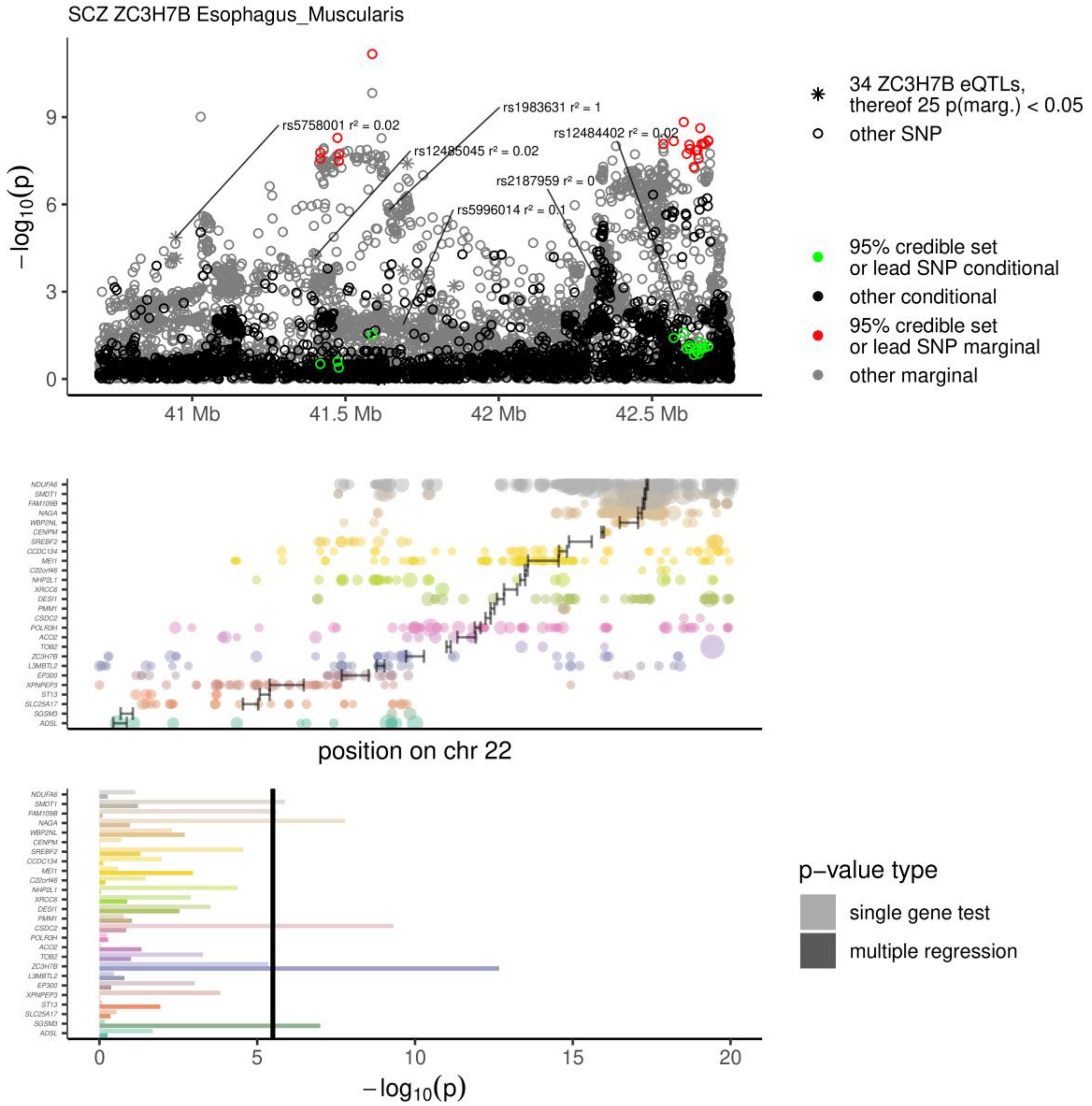

**Supplementary Figure 32. Unabridged summary plot in the style of Figure 3 for UC, *INO80E*, Brain Hippocampus**. This plot shows raw GWAS statistics, GWAS conditioned by eQTL SNPs of the given gene, the location and weight of the eQTLs used by the TWAS analysis, the location of the genes in the locus and their respective unconditioned and conditioned p-values. For a detailed explanation of the plot see **Supplementary Note 2**.

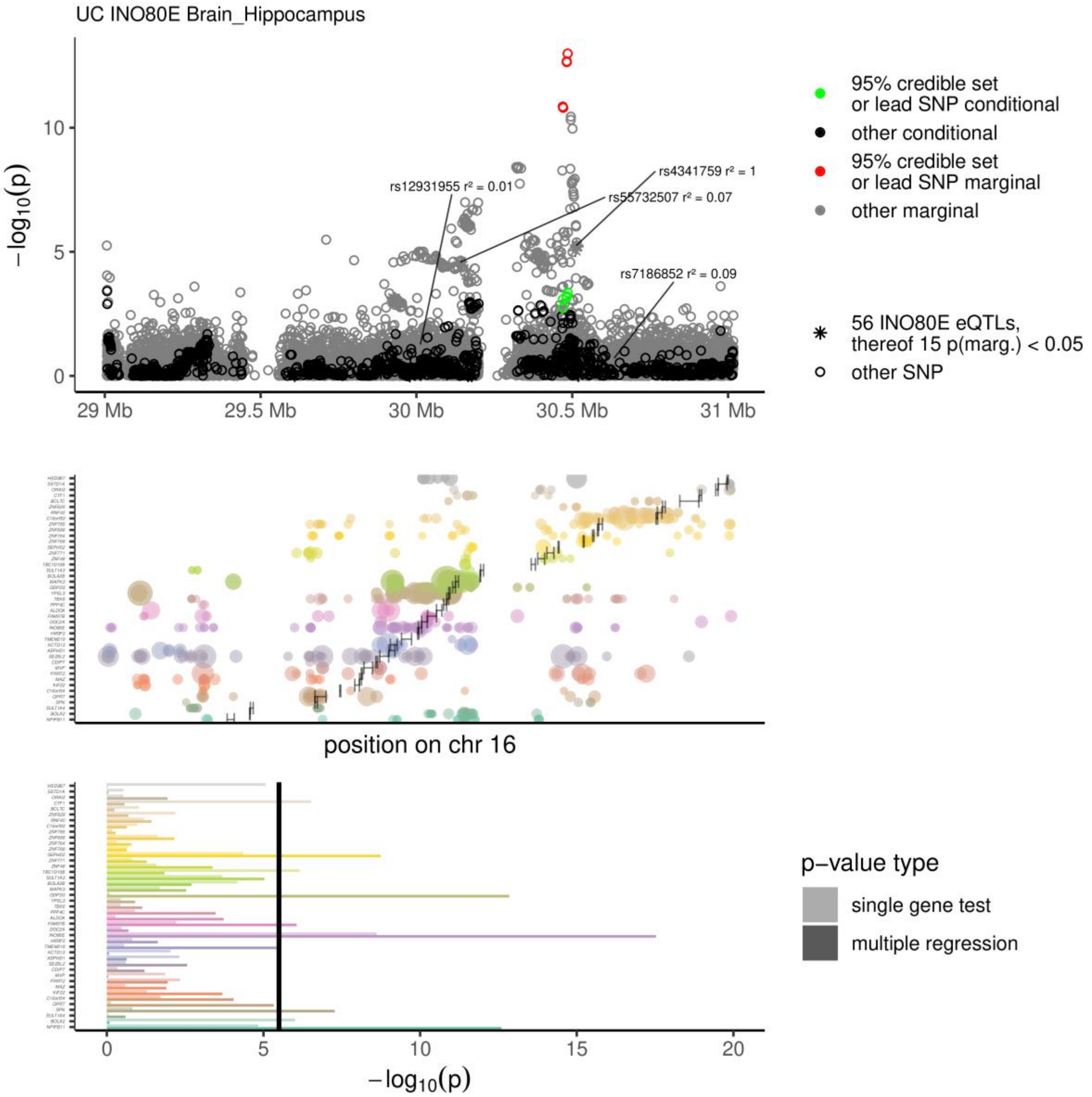

**Supplementary Figure 33. Unabridged summary plot in the style of Figure 3 for UC, *INO80E*, Colon Transverse**. This plot shows raw GWAS statistics, GWAS conditioned by eQTL SNPs of the given gene, the location and weight of the eQTLs used by the TWAS analysis, the location of the genes in the locus and their respective unconditioned and conditioned p-values. For a detailed explanation of the plot see **Supplementary Note 2**.

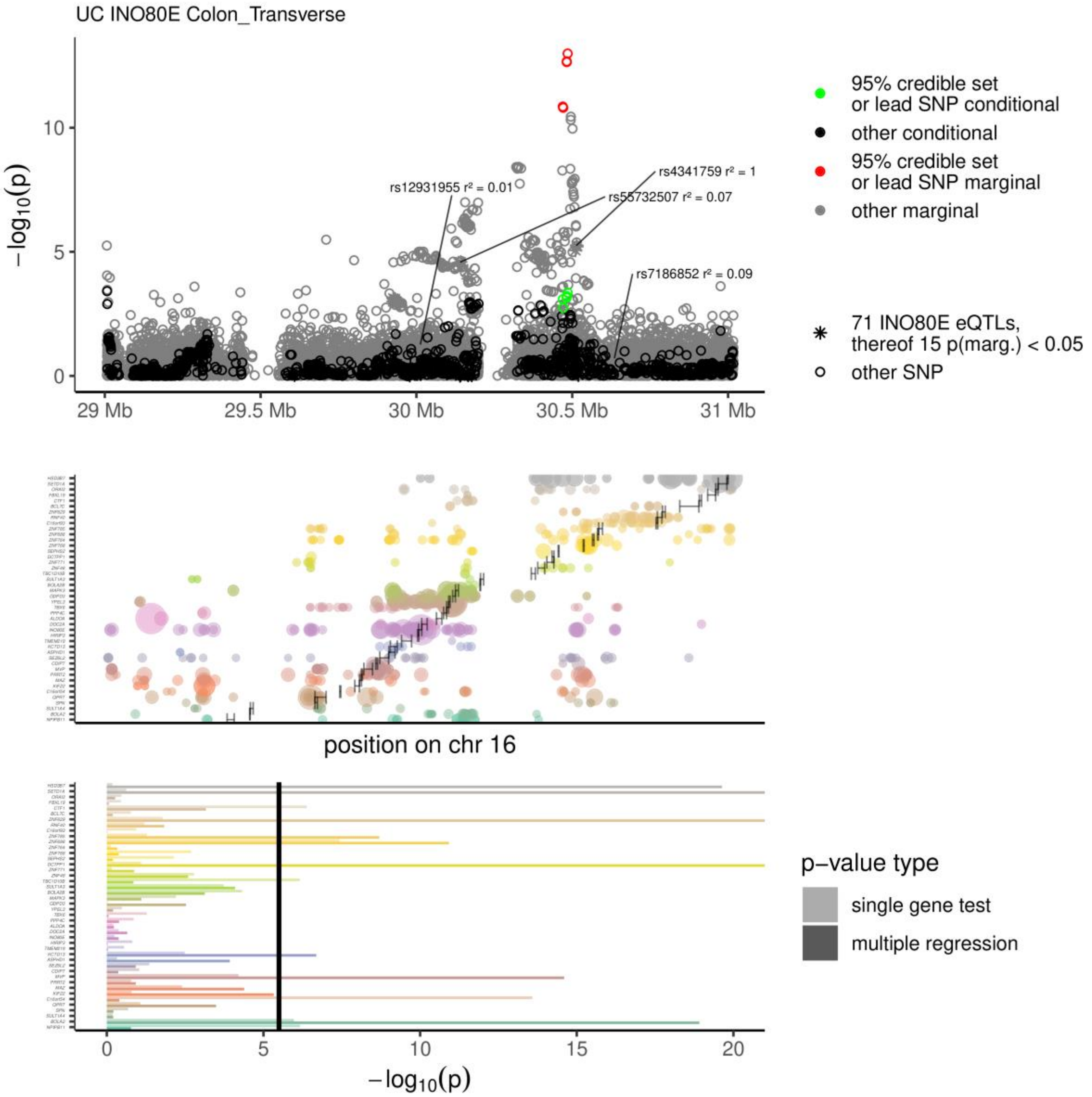

**Supplementary Figure 34. Unabridged summary plot in the style of Figure 3 for UC, *NR5A2*, Brain Hypothalamus**. This plot shows raw GWAS statistics, GWAS conditioned by eQTL SNPs of the given gene, the location and weight of the eQTLs used by the TWAS analysis, the location of the genes in the locus and their respective unconditioned and conditioned p-values. For a detailed explanation of the plot see **Supplementary Note 2**.

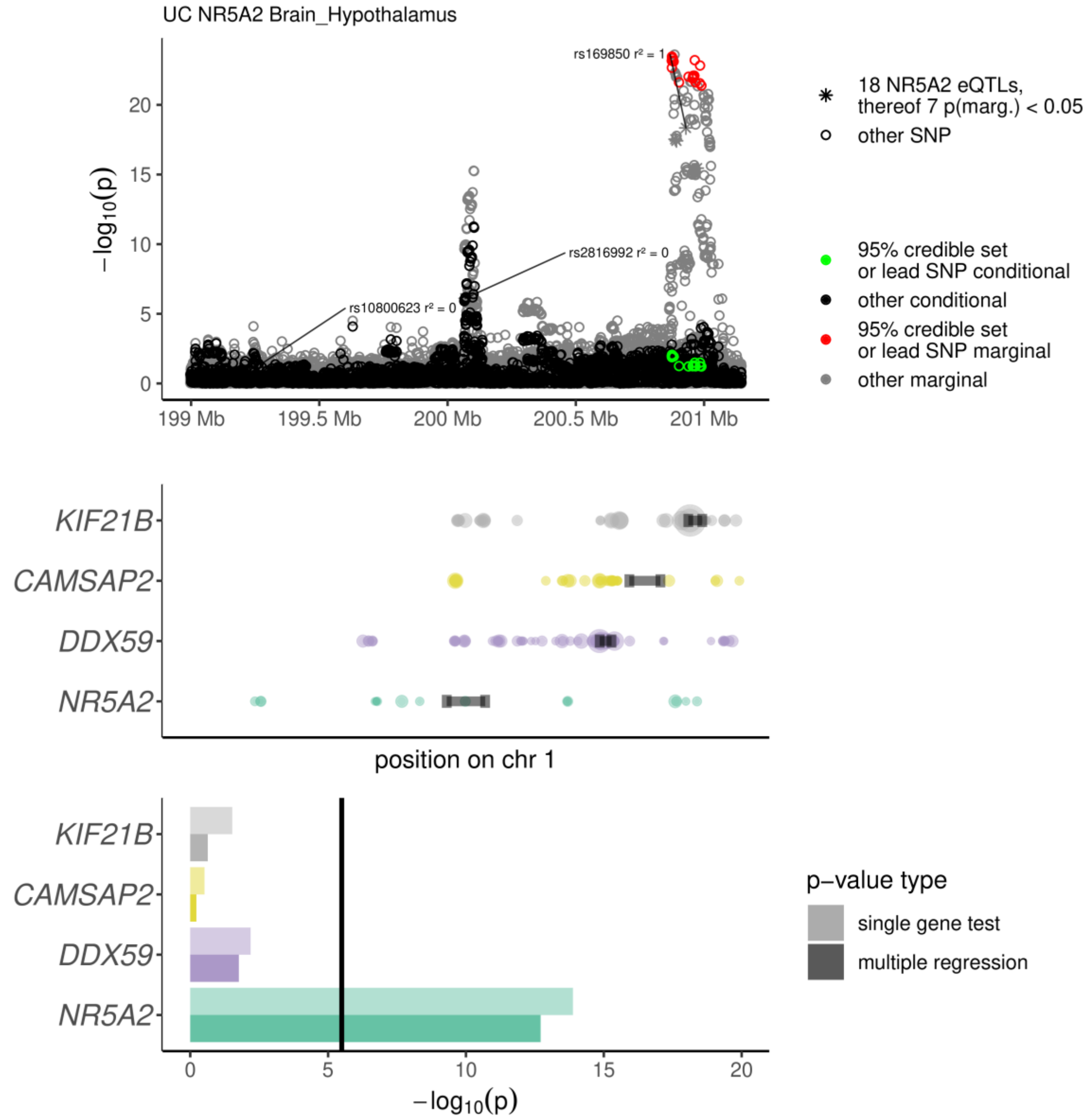

**Supplementary Figure 35. Unabridged summary plot in the style of Figure 3 for UC, *NR5A2*, Colon Transverse**. This plot shows raw GWAS statistics, GWAS conditioned by eQTL SNPs of the given gene, the location and weight of the eQTLs used by the TWAS analysis, the location of the genes in the locus and their respective unconditioned and conditioned p-values. For a detailed explanation of the plot see **Supplementary Note 2**.

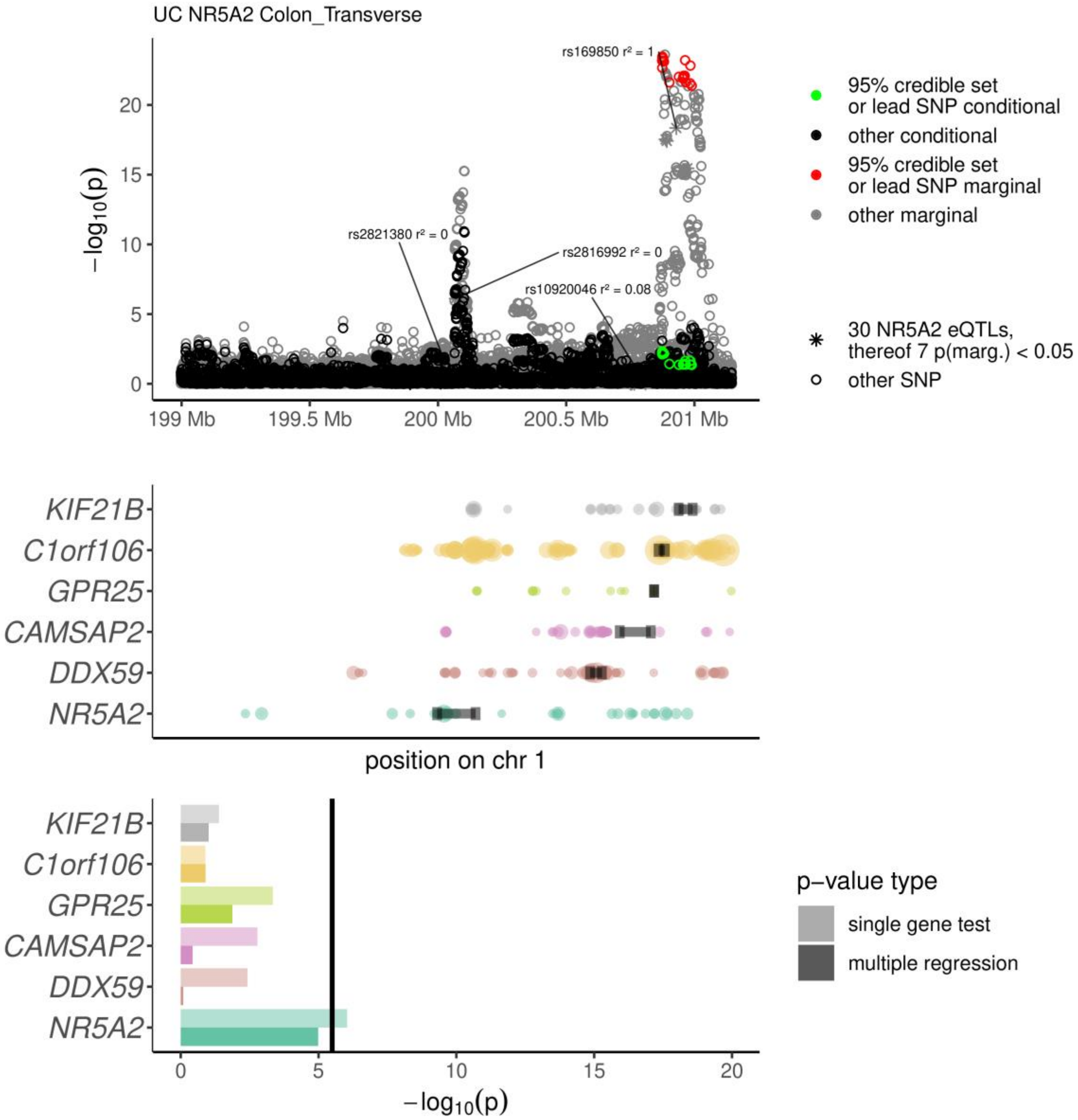

**Supplementary Figure 36. Unabridged summary plot in the style of Figure 3 for UC, *SATB2*, Brain Frontal Cortex BA9**. This plot shows raw GWAS statistics, GWAS conditioned by eQTL SNPs of the given gene, the location and weight of the eQTLs used by the TWAS analysis, the location of the genes in the locus and their respective unconditioned and conditioned p-values. For a detailed explanation of the plot see **Supplementary Note 2**.

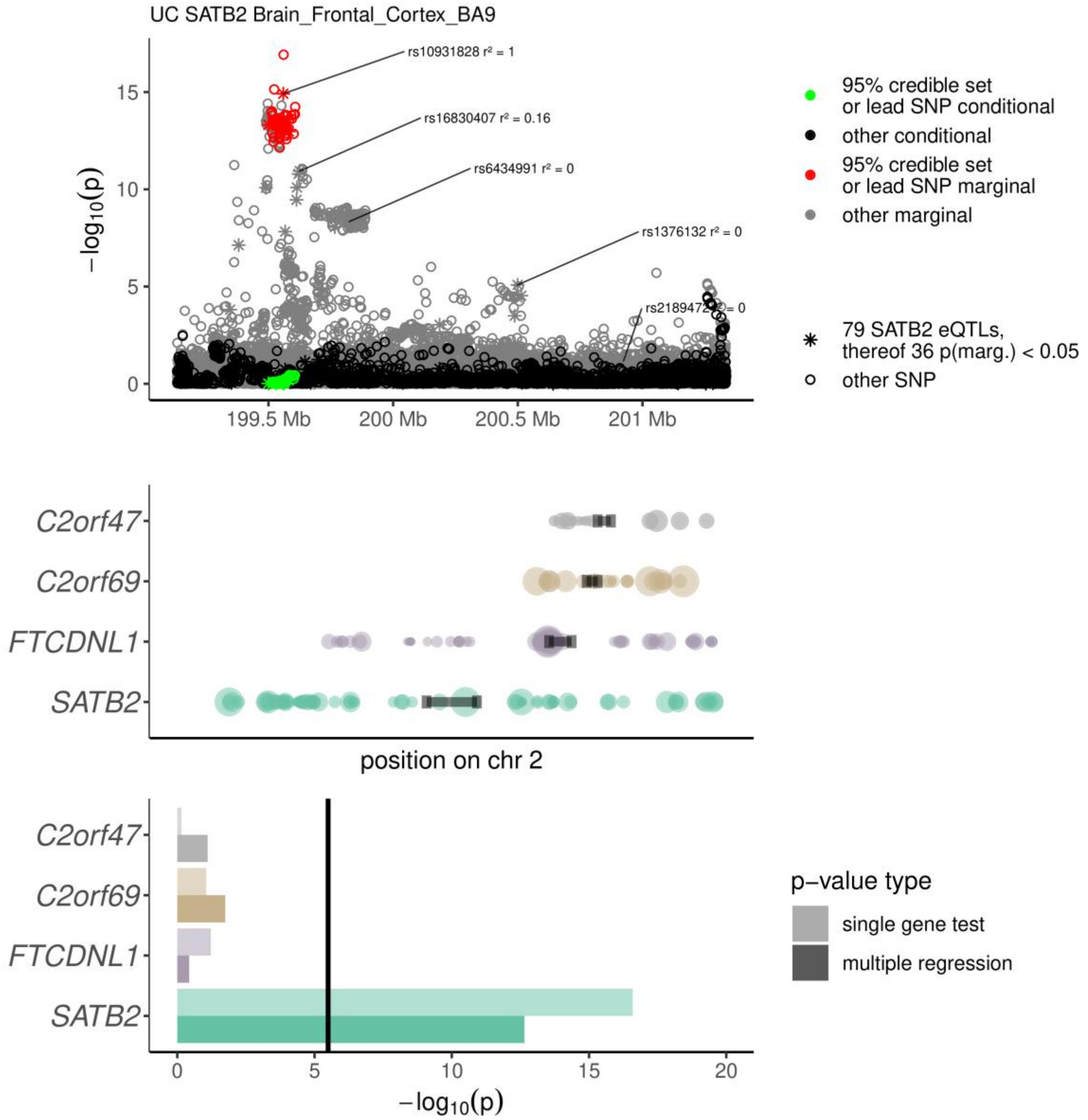

**Supplementary Figure 37. Unabridged summary plot in the style of Figure 3 for UC, *SATB2*, Colon Sigmoid**. This plot shows raw GWAS statistics, GWAS conditioned by eQTL SNPs of the given gene, the location and weight of the eQTLs used by the TWAS analysis, the location of the genes in the locus and their respective unconditioned and conditioned p-values. For a detailed explanation of the plot see **Supplementary Note 2**.

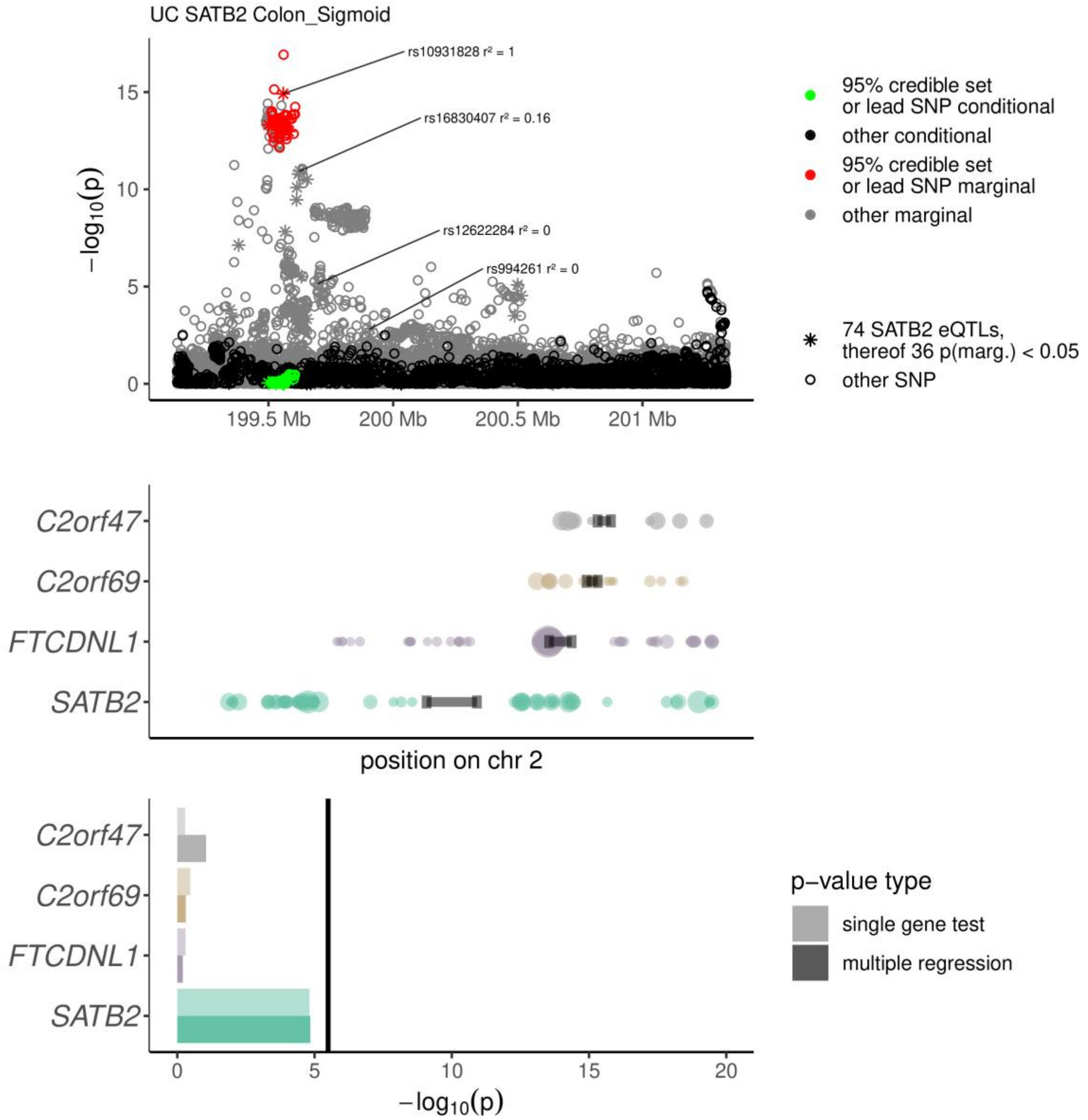

**Supplementary Figure 38. *SF3B1* is one of the candidate genes for SCZ and CD from gene overlap analysis (GBJ$_{conditional}$).** We found that *SF3B1* is most significantly associated with SCZ in the nucleus accumbens basal ganglia (**a**) and with CD in the sigmoid colon (**d**), but only marginally *vice versa* (**b**, **c**). A shared signal for SCZ and CD is probable due to the colocalization. Despite the broad GWAS association signal, no other genes at this locus are GBJ significant in either disease, suggesting an implication of *SF3B1*. Although it might be coincidence, we note that the *SATB2* locus displayed in **Figure 3** is adjacent to this locus. The *SATB2* locus shown in **Figure 3** is adjacent to this locus, but is more than 1MB away from it.

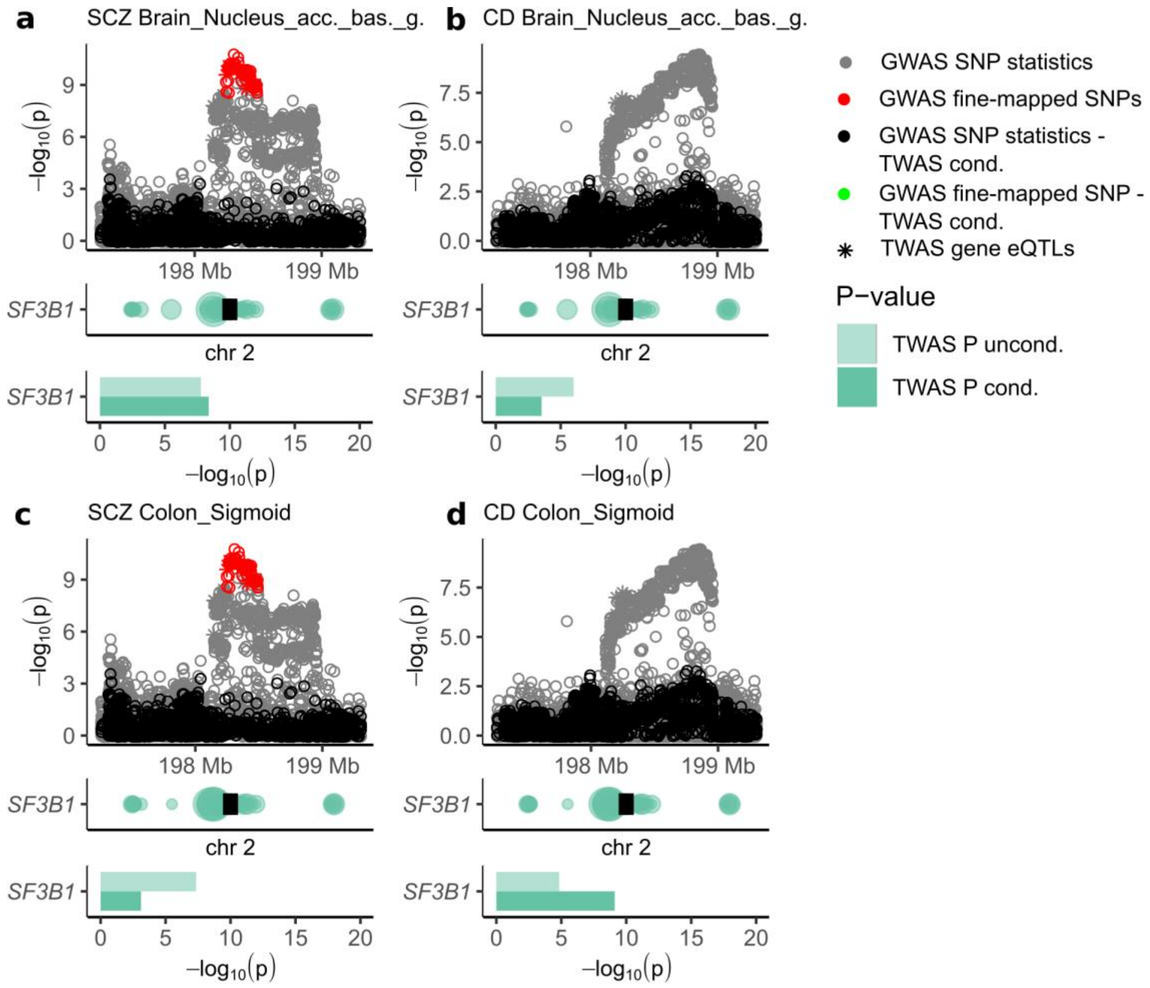

**Supplementary Figure 39. *INO80E* is a candidate gene for SCZ and UC from gene overlap analysis (GBJ$_{conditional}$).** *INO80E* is significant in SCZ in transverse colon (**a**) and in UC in brain hippocampus (**d**), but not *vice versa* (**b**, **c**). *INO80E*, which has already been reported as a shared TWAS gene of schizophrenia and cardiometabolic disorders[58], was detected for UC (hippocampus) and SCZ (colon transverse). Of note, in the same study, *MAPK3* was also reported for schizophrenia and cardiometabolic disorders as a susceptibility gene that is 100kb away from *INO80E*. A previous TWAS study for schizophrenia[59] identified *MAPK3* as a susceptibility gene for schizophrenia.

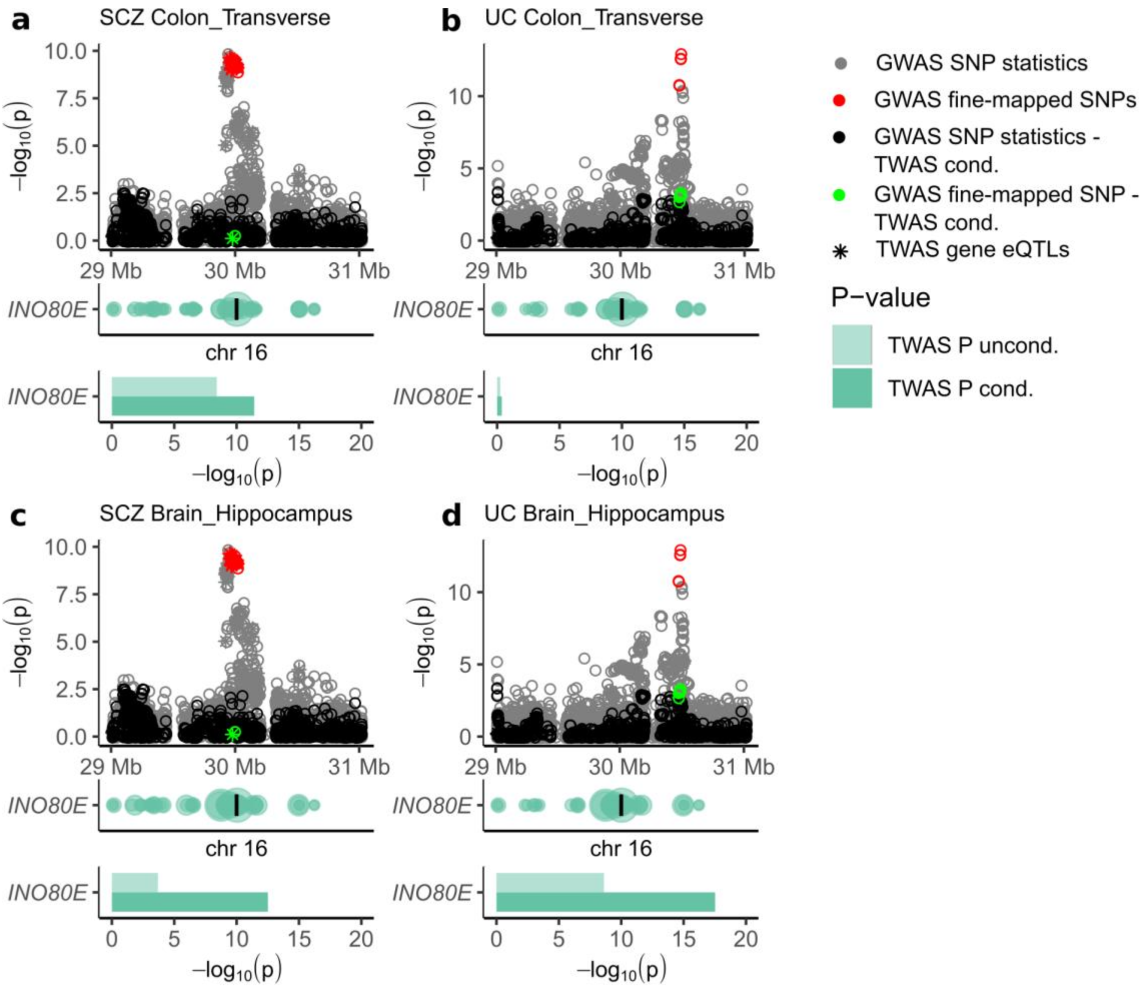

**Supplementary Figure 40. *SGSM3* is a candidate gene for SCZ and CD from gene overlap analysis ($GBJ_{conditional}$).** On chromosome 22, we found two GBJ hits, *SGSM3* and *ZC3H7B* (**Supplementary Figure 41**), which are shared between CD and SCZ. These two genes are less than 1 Mb apart, so they may not be statistically independent. In SCZ, *SGSM3* is strongest in the sigmoid colon (**a**), while in CD it is strongest in the transverse colon (**d**). Interestingly, *SGSM3* is not even suggestively associated in sigmoid colon in CD (**b**) and only marginally associated with SCZ in transverse colon (**c**). In SCZ, the conditional GWAS statistics are not fully explained by the eQTLs of the two genes. In CD, the region starting at 41Mb is well explained by the eQTLs; the locus lacked genome-wide significance in previous GWAS studies for CD. The incomplete explanation of the GWAS signal in both diseases suggests that the genetically regulated expression of *SGSM3* and *ZC3H7B* are not the only or causal features of this locus.

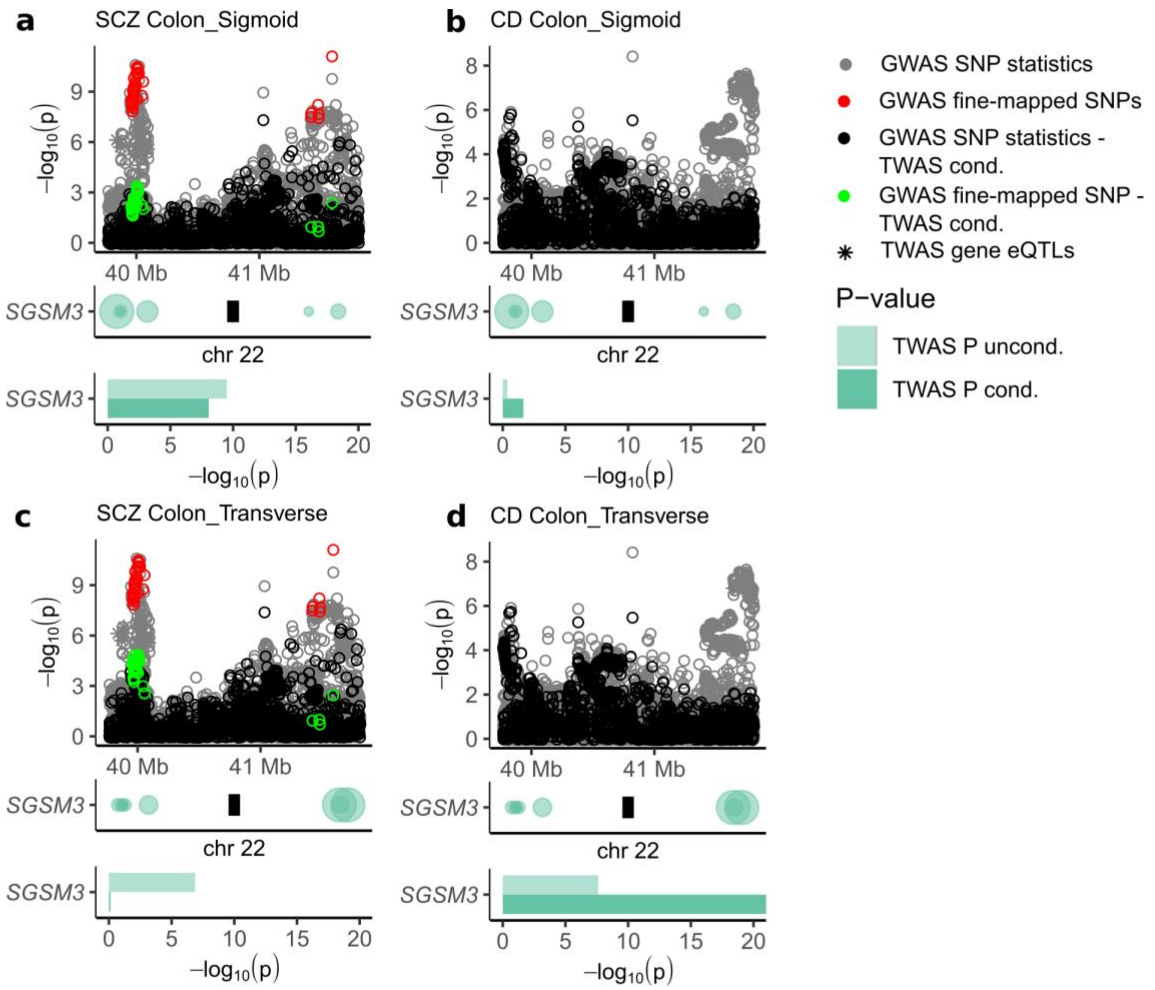

**Supplementary Figure 41.** ***ZC3H7B*** **is a candidate gene for SCZ and CD from gene overlap analysis (GBJ$_{\text{conditional}}$) and is significant in two gastrointestinal tissues in SCZ and CD.** In SCZ, *ZC3H7B* is strongest in esophagus muscularis (**a**), in CD it is strongest in transverse colon (**d**). *Vice versa*, both genes are marginally significant (**b**, **c**). In SCZ, the conditioned GWAS stats are not fully explained by the eQTLs of the two genes, there are many SNP-disease associations still in the range of 1e-6 for *P*-values. In CD, the region starting at 41Mb is well explained by the eQTLs; the locus lacked genome-wide significance in previous GWAS studies for CD. The incomplete explanation of the GWAS signal in SCZ suggests that the genetically regulated expression of *ZC3H7B* are not the only or causal features of this locus. Although *ZC3H7B* appears to be a good candidate based on the strong TWAS *P*-value and explanation of the GWAS signal in CD, it should be noted that in CD, six other genes are also GBJ-conditionally transcriptome-wide significant at this locus, so further research is needed to identify a single candidate gene.

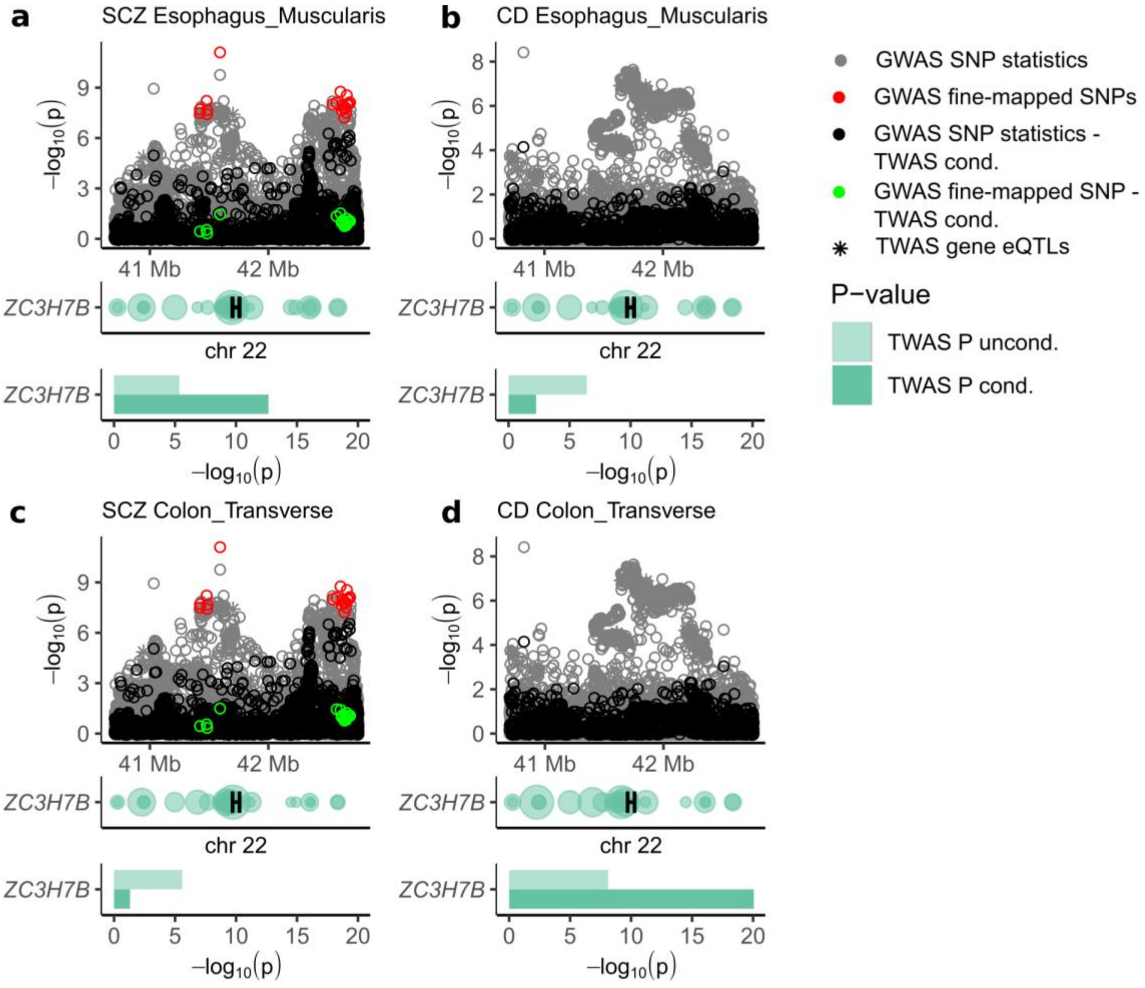

**Supplementary Figure 42. Analysis of bulk and scRNA-seq data for *NR5A2*, *SATB2*, and *PPP3CA* identified by primary gene overlap analysis (Single-tissue$_{conditional}$), and *INO80E*, *SGSM3* and *ZC3H7B* identified by an extended gene overlap analysis (GBJ$_{conditional}$).** Baseline expression data from six reference studies **(Methods)** were evaluated to check whether expression patterns support their suggested role in SCZ and CD/UC. Results from adult tissue (**a**), from developmental samples (**b**) and single cell studies (**c**) help to understand the roles of our candidate genes in the gut-brain-axis. For a detailed explanation of the results, see **Results** and **Supplementary Note 5**. *(exPFC=glutamatergic neurons from the PFC, exCA1/3=pyramidal neurons from the Hip CA region, GABA=GABAergic interneurons, exDG=granule neurons from the Hip dentate gyrus region, ASC=astrocytes, NSC=neuronal stem cells, MG=microglia, ODC=oligodendrocytes, OPC=oligodendrocyte precursor cells, NSC=neuronal stem cells, SMC=smooth muscle cells, END= endothelial cells).*

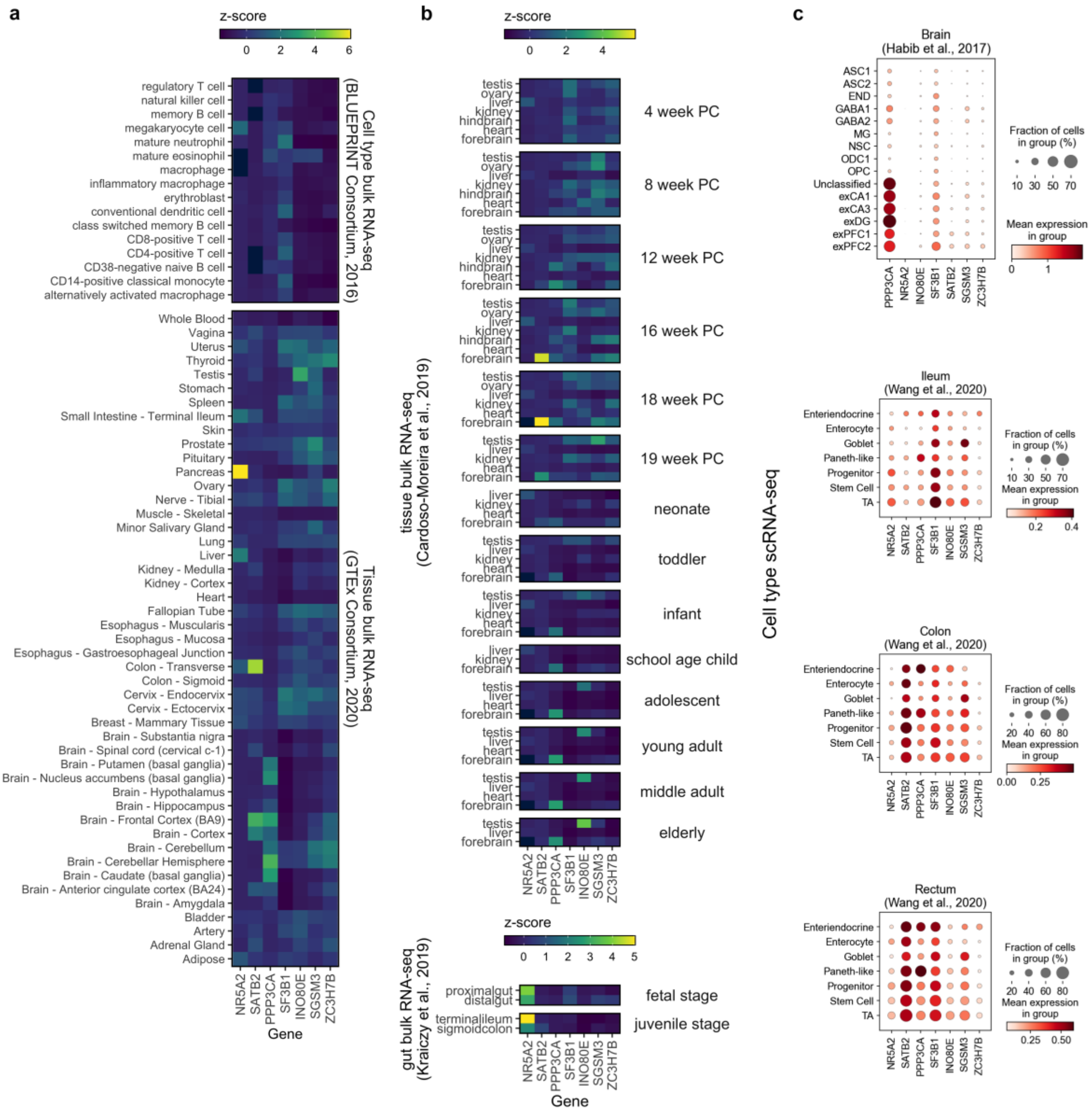
a
z-score
0 2 4 6
regulatory T cell
natural killer cell
memory B cell
megakaryocyte cell
mature neutrophil
mature eosinophil
macrophage
inflammatory macrophage
erythroblast
conventional dendritic cell
class switched memory B cell
CD8-positive T cell
CD4-positive T cell
CD38-negative naive B cell
CD14-positive classical monocyte
alternatively activated macrophage
Cell type bulk RNA-seq (BLUEPRINT Consortium, 2016)
Whole Blood
Vagina
Uterus
Thyroid
Testis
Stomach
Spleen
Small Intestine - Terminal Ileum
Skin
Prostate
Pituitary
Pancreas
Ovary
Nerve - Tibial
Muscle - Skeletal
Minor Salivary Gland
Lung
Liver
Kidney - Medulla
Kidney - Cortex
Heart
Fallopian Tube
Esophagus - Muscularis
Esophagus - Mucosa
Esophagus - Gastroesophageal Junction
Colon - Transverse
Colon - Sigmoid
Cervix - Endocervix
Cervix - Ectocervix
Breast - Mammary Tissue
Brain - Substantia nigra
Brain - Spinal cord (cervical c-1)
Brain - Putamen (basal ganglia)
Brain - Nucleus accumbens (basal ganglia)
Brain - Hypothalamus
Brain - Hippocampus
Brain - Frontal Cortex (BA9)
Brain - Cortex
Brain - Cerebellum
Brain - Cerebellar Hemisphere
Brain - Caudate (basal ganglia)
Brain - Anterior cingulate cortex (BA24)
Brain - Amygdala
Bladder
Artery
Adrenal Gland
Adipose
Tissue bulk RNA-seq (GTEx Consortium, 2020)
NR5A2 SATB2 PPP3CA SF3B1 INO80E SGSM3 ZC3H7B
Gene
b
z-score
0 2 4
testis ovary liver kidney hindbrain heart forebrain
4 week PC
8 week PC
12 week PC
16 week PC
18 week PC
19 week PC
neonate
toddler
infant
school age child
adolescent
young adult
middle adult
elderly
tissue bulk RNA-seq (Cardoso-Moreira et al., 2019)
NR5A2 SATB2 PPP3CA SF3B1 INO80E SGSM3 ZC3H7B
z-score
0 1 2 3 4 5
proximalgut distalgut
fetal stage
terminalileum sigmoidcolon
juvenile stage
gut bulk RNA-seq (Kraiczy et al., 2019)
NR5A2 SATB2 PPP3CA SF3B1 INO80E SGSM3 ZC3H7B
Gene
c
Cell type scRNA-seq
Brain (Habib et al., 2017)
ASC1
ASC2
END
GABA1
GABA2
MG
NSC
ODC1
OPC
Unclassified
exCA1
exCA3
exDG
exPFC1
exPFC2
PPP3CA NR5A2 INO80E SF3B1 SATB2 SGSM3 ZC3H7B
Fraction of cells in group (%)
10 30 50 70
Mean expression in group
0 1
Ileum (Wang et al., 2020)
Enteriendocrine
Enterocyte
Goblet
Paneth-like
Progenitor
Stem Cell
TA
NR5A2 SATB2 PPP3CA SF3B1 INO80E SGSM3 ZC3H7B
Fraction of cells in group (%)
10 30 50 70
Mean expression in group
0.2 0.4
Colon (Wang et al., 2020)
Enteriendocrine
Enterocyte
Goblet
Paneth-like
Progenitor
Stem Cell
TA
NR5A2 SATB2 PPP3CA SF3B1 INO80E SGSM3 ZC3H7B
Fraction of cells in group (%)
20 40 60 80
Mean expression in group
0.00 0.25
Rectum (Wang et al., 2020)
Enteriendocrine
Enterocyte
Goblet
Paneth-like
Progenitor
Stem Cell
TA
NR5A2 SATB2 PPP3CA SF3B1 INO80E SGSM3 ZC3H7B
Fraction of cells in group (%)
20 40 60 80
Mean expression in group
0.25 0.50

## Members of the International Inflammatory Bowel Disease Genetics Consortium (IIBDGC)

Shifteh Abedian[1,2], Clara Abraham[3], Jean-Paul Achkar[4,5], Tariq Ahmad[6], Rudi Alberts[7], Behrooz Alizadeh[2], Leila Amininejad[8,9], Ashwin N Ananthakrishnan[20,11], Vibeke Andersen[12,13], Carl A Anderson[14], Jane M Andrews[15], Vito Annese[16,17], Guy Aumais[18,19], Robert N Baldassano[20], Peter A Bampton[21], Murray Barclay[22], Jeffrey C Barrett[14], Johannes Bethge[23], Claire Bewshea[6], Joshua C Bis[24], Alain Bitton[25], Thelma BK[26], Gabrielle Boucher[27], Oliver Brain[28], Stephan Brand[29], Steven R Brant[30,31], Jae Hee Cheon[32], Judy H Cho[33], Isabelle Cleynen[34], Ariella Cohain[35], Rachel Cooney[36], Anthony Croft[37], Mauro D'Amato[38,39], Renata D'Inca[40], Mark J Daly[41,42], Silvio Danese[43], Naser Ebrahim Daryani[1], Lisa Wu Datta[30], Frauke Degenhardt[44], Goda Denapiene[45], Lee A Denson[46], Kathy L Devaney[10], Olivier Dewit[47], Hazel E Drummond[48], Marla Dubinsky[49], Richard H Duerr[50,51], Cathryn Edwards[52], David Ellinghaus[44], Pierre Ellul[53], Motohiro Esaki[54], Jonah Essers[55,56], Lynnette R Ferguson[57], Eleonora A Festen[7], Philip Fleshner[58], Tim Florin[59], Denis Franchimont[8,9], Andre Franke[44], Yuta Fuyuno[54,60], Richard Gearry[22,61], Michel Georges[62,63], Christian Gieger[64], Jürgen Glas[25], Philippe Goyette[65], Todd Green[42,55], Anne M Griffiths[66], Stephen L Guthery[67], Hakon Hakonarson[68,69], Jonas Halfvarson[70], Katherine Hanigan[71], Talin Haritunians[58], Ailsa Hart[72], Chris Hawkey[73], Nicholas K Hayward[74], Matija Hedl[3], Paul Henderson[75], Georgina L Hold[76], Myhunghee Hong[77], Xinli Hu[78], Hailiang Huang[41,79], Jean-Pierre Hugot[80], Ken Y Hui[68], Marcin Imielinski[58], Omid Jazayeri[81], Laimas Jonaitis[82], Luke Jostins[83], Garima Juyal[84], Ramesh Chandra Juyal[85], Rahul Kalla[48], Tom H Karlsen[86,87], Nicholas A Kennedy[88], Mohammed Azam Khan[89], Won Ho Kim[90], Takanari Kitazono[54], Gediminas Kiudelis[82], Krupa Krishnaprasad[91], Michiaki Kubo[92], Subra Kugathasan[93], Limas Kupcinskas[94], Christopher A Lamb[95], Katrina M de Lange[14], Anna Latiano[16], Debby Laukens[96], Ian C Lawrance[97], James C Lee[98], Charlie W Lees[48], Marcis Leja[99], Nina Lewis[73], Johan Van Limbergen[66], Paolo Lionetti[100], Jimmy Z Liu[14], Anton Lord[101], Edouard Louis[102], Yang Luo[14], Stuart MacGregor[103], Gillian Mahy[104], Masoud Mohammad Malekzadeh[105,107], Reza Malekzadeh[106], John Mansfield[108], Suzie Marriott[6], Dunecan Massey[109], Christopher G Mathew[110], Toshiyuki Matsui[111], Dermot PB McGovern[58], Andrea van der Meulen[112], Vandana Midha[113], Raquel Milgrom[114], Samaneh Mirzaei[105,107], Mitja Mitrovic[115,81], Grant W Montgomery[116], Craig Mowat[117], Christoph Müller[118], William G Newman[89], Aylwin Ng[10,120], Siew C Ng[119], Sok Meng Evelyn Ng[3], Susanna Nikolaus[23], Kaida Ning[3], Markus Nöthen[121], Ioannis Oikonomou[3], David T Okou[122], Timothy R Orchard[123], Orazio Palmieri[16], Miles Parkes[109], Anne Phillips[117], Cyriel Y Ponsioen[124], Urõs Potocnik[125,126], Hossein Poustchi[105,107], Natalie J Prescott[110], Deborah D Proctor[3], Graham Radford-Smith[127,128,129], Jean- Francois Rahier[130], Walter Reinisch[131], Florian Rieder[4], John D Rioux[27], Rebecca Roberts[22], Gerhard Rogler[132], Richard K Russell[133], Jeremy D Sanderson[134], Miquel Sans[135], Jack Satsangi[48], Eric E Schadt[35], Michael Scharl[132], John Schembri[53], Stefan Schreiber[23,44], L Philip Schumm[136], Regan Scott[50], Mark Seielstad[137,138], Tejas Shah[14], Yashoda Sharma[3], Mark S Silverberg[114], Alison Simmons[28], Lisa A Simms[37], Abhey Singh[6], Jurgita Skieceviciene[82], Suzanne van Sommeren[7], Kyuyoung Song[77], Ajit Sood[113], Sarah L Spain[110], A. Hillary Steinhart[114], Joanne M Stempak[114], Laura Stronati[139], Joseph JY Sung[119], Atsushi Takahashi[140], Stephan R Targan[58], Kirstin M Taylor[134], Emilie Theatre[62,63], Leif Torkvist[141], Esther A Torres[142], Mark Tremelling[143], Holm H Uhlig[144], Junji Umeno[54], Homayon Vahedi[145], Eric Vasiliauskas[58], Anje ter Velde[124], Nicholas T Ventham[48], Severine Vermeire[146,147], Hein W Verspaget[112], Martine De Vos[96], Thomas Walters[148,66], Kai Wang[68], Ming-Hsi Wang[149,4], Rinse K Weersma[150], Zhi Wei[151], David Whiteman[74], Cisca Wijmenga[81], David C Wilson[133,75], Juliane Winkelmann[152,153], Sunny H Wong[119], Ramnik J

Xavier[10,42], Keiko Yamazaki[154,60], Suk-Kyun Yang[155], Byong Duk Ye[155], Sebastian Zeissig[156], Bin Zhang[35], Clarence K Zhang[157], Hu Zhang[158,159], Wei Zhang[3], Hongyu Zhao[157], Zhen Z Zhao[74], Belgium IBD Genetics Consortium, Italian Group for IBD Genetic Consortium, NIDDK Inflammatory Bowel Disease Genetics Consortium, Quebec IBD Genetics Consortium, United Kingdom IBDGC, Wellcome Trust Case Control Consortium
1 Digestive Disease Research Center, Digestive Disease Research Institute, Tehran University of Medical Sciences, Tehran, Iran.
2 Department of Epidemiology, University Medical Center Groningen, Groningen, The Netherlands.
3 Section of Digestive Diseases, Department of Internal Medicine, Yale School of Medicine, NewHaven, Connecticut, USA.
4 Department of Gastroenterology and Hepatology, Digestive Disease Institute, Cleveland Clinic, Cleveland, Ohio, USA.
5 Department of Pathobiology, Lerner Research Institute, Cleveland Clinic, Cleveland, Ohio, USA.
6 Peninsula College of Medicine and Dentistry, Exeter, UK.
7 Department of Gastroenterology and Hepatology, University Medical Center Groningen, Groningen, The Netherlands.
8 Department of Gastroenterology, Erasmus Hospital, Brussels, Belgium.
9 Department of Gastroenterology, Free University of Brussels, Brussels, Belgium.
10 Gastroenterology Unit, Massachusetts General Hospital, Harvard Medical School, Boston, Massachusetts, USA.
11 Division of Medical Sciences, Harvard Medical School, Boston, Massachusetts, USA.
12 Focused Research Unit for Molecular Diagnostic and Clinical Research, IRS-Center Soenderjylland, University Hospital of Southern Denmark, Denmark.
13 Institute of Molecular Medicine University of Southern Denmark, Denmark.
14 Wellcome Trust Sanger Institute, Hinxton, UK.
15 Inflammatory Bowel Disease Service, Department of Gastroenterology and Hepatology, Royal Adelaide Hospital, Adelaide, Australia.
16 Unit of Gastroenterology, Istituto di Ricovero e Cura a Carattere Scientifico-Casa Sollievo della Sofferenza (IRCCSCSS) Hospital, San Giovanni Rotondo, Italy.
17 Strutture Organizzative Dipartimentali (SOD) Gastroenterologia 2, Azienda Ospedaliero Universitaria (AOU) Careggi, Florence, Italy.
18 Department of Gastroenterology, Hôpital Maisonneuve-Rosemont, Montréal, Québec, Canada.
19 Faculté de Médecine, Université de Montréal, Montréal, Québec, Canada.
20 Center for Applied Genomics, The Childrens Hospital of Philadelphia, Philadelphia, Pennsylvania, USA.
21 Department of Gastroenterology and Hepatology, Flinders Medical Centre and School of Medicine, Flinders University, Adelaide, Australia.
22 Department of Medicine, University of Otago, Christchurch, New Zealand.
23 Department for General Internal Medicine, Christian-Albrechts-University, Kiel, Germany.
24 Cardiovascular Health Research Unit, University of Washington, Seattle, Washington, USA.
25 Division of Gastroenterology, Royal Victoria Hospital, Montréal, Québec, Canada.
26 Department of Genetics, University of Delhi South Campus, New Delhi, India.
27 Université de Montréal and the Montréal Heart Institute, Research Center, Montréal Heart Institute, Montréal, Québec, Canada.
28 Translational Gastroenterology Unit, John Radcliffe Hospital, Oxford, UK.

29 Department of Medicine II, Ludwig-Maximilians-University Hospital Munich-Grosshadern, Munich, Germany.
30 Harvey M. and Lyn P. Meyerhoff Inflammatory Bowel Disease Center, Department of Medicine, John Hopkins University School of Medicine, Baltimore, USA.
31 Department of Medicine, Rutgers Robert Wood Johnson School of Medicine and Department of Genetics, Rutgers University Brunswick and Piscataway, New Jersey, USA.
32 Department of Internal Medicine and Institute of Gastroenterology, Yonsei University College of Medicine, Seoul, Korea.
33 Department of Genetics, Yale School of Medicine, New Haven, Connecticut, USA.
34 Department of Human Genetics, KU Leuven, Leuven, Belgium.
35 Department of Genetics and Genomic Sciences, Mount Sinai School of Medicine, New York, New York, USA.
36 Department of Gastroenterology, Sandwell and West Birmingham Hospitals NHS Trust, Birmingham, UK.
37 Inflammatory Bowel Diseases,Department of Immunology, QIMR Berghofer Medical Research Institute, Brisbane, Australia.
38 School of Biological Sciences, Monash University, Clayton VIC, Australia.
39 Department of Medicine Solna, Karolinska Institutet, Stockholm, Sweden.
40 Division of Gastroenterology, University Hospital Padua, Padua, Italy.
41 Analytic and Translational Genetics Unit, Massachusetts General Hospital, Harvard Medical School, Boston, Massachusetts, USA.
42 Broad Institute of MIT and Harvard, Cambridge, 24 Massachusetts, USA.
43 IBD Center, Department of Gastroenterology, Istituto Clinico Humanitas, Milan, Italy.
44 Institute of Clinical Molecular Biology, Christian-Albrechts-University, Kiel, Germany.
45 Department of Gastroenterology and Hepatology, Radboud University Nijmegen Medical Centre, Nijmegen, The Netherlands.
46 Pediatric Gastroenterology, Cincinnati Childrens Hospital Medical Center, Cincinnati, Ohio, USA.
47 Department of Gastroenterology, Université Catholique de Louvain (UCL) Cliniques Universitaires Saint-Luc, Brussels, Belgium.
48 Gastrointestinal Unit, Centre for Genomic and Experimental Medicine, Institute of Genetics and Molecular Medicine, University of Edinburgh, Edinburgh, UK.
49 Department of Pediatrics, Cedars Sinai Medical Center, Los Angeles, California, USA.
50 Division of Gastroenterology, Hepatology and Nutrition, Department of Medicine, University of Pittsburgh School of Medicine, Pittsburgh, Pennsylvania, USA.
51 Department of Human Genetics, University of Pittsburgh Graduate School of Public Health, Pittsburgh, Pennsylvania, USA.
52 Department of Gastroenterology, Torbay Hospital, Torbay, Devon, UK.
53 Department of Gastroenterology, Mater Dei Hospital, Msida, Malta.
54 Department of Medicine and Clinical Science, Graduate School of Medical Sciences, Kyushu University, Fukuoka, Japan.
55 Center for Human Genetic Research, Massachusetts General Hospital, Harvard Medical School, Boston, Massachusetts, USA.
56 Pediatrics, Harvard Medical School, Boston, Massachusetts, USA.
57 Faculty of Medical & Health Sciences, School of Medical Sciences, The University of Auckland, Auckland, New Zealand.

58 F.Widjaja Foundation Inflammatory Bowel and Immunobiology Research Institute, Cedars-Sinai Medical Center, Los Angeles, California, USA.
59 Department of Gastroenterology, Mater Health Services, Brisbane, Australia.
60 Laboratory for Genotyping Development, Center for Integrative Medical Sciences, Riken, Yokohama, Japan.
61 Department of Gastroenterology, Christchurch Hospital, Christchurch, New Zealand.
62 Unit of Animal Genomics, Groupe Interdisciplinaire de Génoprotéomique Appliquée (GIGA-R) Research Center, University of Liege, Liege, Belgium.
63 Faculty of Veterinary Medicine, University of Liege, Liege, Belgium.
64 Institute of Genetic Epidemiology, Helmholtz Zentrum München - German Research Center for Environmental Health, Neuherberg, Germany.
65 Research Center, Montreal Heart Institute, Montréal, Québec, Canada.
66 Division of Pediatric Gastroenterology, Hepatology and Nutrition, Hospital for Sick Children, Toronto, Ontario, Canada.
67 Department of Pediatrics, University of Utah School of Medicine, Salt Lake City, Utah, USA.
68 Center for Applied Genomics, The Children's Hospital of Philadelphia, Philadelphia, Pennsylvania, USA.
69 Department of Pediatrics, The Perelman School of Medicine, University of Pennsylvania, Philadelphia, PA, 19104.
70 Department of Gastroenterology, Faculty of Medicine and Health, Örebro University, SE 702 81, Örebro, Sweden.
71 Inflammatory Bowel Diseases, Genetics and Computational Biology, Queensland Institute of Medical Research, Brisbane, Australia.
72 IBD Unit, St Marks Hospital, Harrow, Middlesex, UK.
73 Nottingham Digestive Diseases Centre, Queens Medical Centre, Nottingham, UK.
74 Molecular Epidemiology, Genetics and Computational Biology, Queensland Institute of Medical Research, Brisbane, Australia.
75 Child Life and Health, University of Edinburgh, Edinburgh, Scotland, UK.
76 Gastrointestinal Research Group, Division of Applied Medicine, University of Aberdeen, Aberdeen, UK.
77 Department of Biochemistry and Molecular Biology, University of Ulsan College of Medicine, Seoul, Korea.
78 Division of Rheumatology Immunology and Allergy, Brigham and Womens Hospital, Boston, Massachusetts, USA.
79 Broad Institute of MIT and Harvard, Cambridge, Massachusetts, USA.
80 Centre de recherche sur l’inflammation, UMR1149 INSERM et Université de Paris, Paris, France.
81 Department of Genetics, University Medical Center Groningen, Groningen, The Netherlands.
82 Academy of Medicine, Lithuanian University of Health Sciences, Kaunas, Lithuania.
83 Wellcome Trust Centre for Human Genetics, University of Oxford, Headington, UK.
84 School of Biotechnology, Jawaharlal Nehru University, New Delhi, India.
85 National Institute of Immunology, Aruna Asaf Ali Road, New Delhi, India.
86 Norwegian PSC Research Center, Department of Transplantation Medicine, Division of Surgery, Inflammatory Diseases and Transplantation, Oslo University Hospital Rikshospitalet, Oslo, Norway.
87 Research Institute for Internal Medicine, Division of Surgery, Inflammatory Diseases and Transplantation, Oslo University Hospital Rikshospitalet and University of Oslo, Oslo, Norway.

88 IBD Pharmacogenetics Group, University of Exeter, Exeter, UK.
89 Manchester Centre for Genomic Medicine, University of Manchester and Central Manchester University Hospitals NHS Foundation Trust, Manchester, UK.
90 Yonsei University College of Medicine, Seoul, Korea.
91 Department of Immunology, QIMR Berghofer Medical Research Institute, Brisbane,Australia.
92 RIKEN Center for Integrative Medical Sciences, Yokohama, Japan.
93 Department of Pediatrics, Emory University School of Medicine, Atlanta, Georgia, USA.
94 Department of Gastroenterology, Kaunas University of Medicine, Kaunas, Lithuania.
95 Institute of Cellular Medicine, Newcastle University, Newcastle-upon-Tyne, UK.
96 Department of Hepatology and Gastroenterology, Ghent University Hospital, Ghent, Belgium.
97 School of Medicine and Pharmacology, University of Western Australia, Fremantle, Australia.
98 Department of Medicine, University of Cambridge, UK.
99 Faculty of Medicine, University of Latvia, Riga, Latvia.
100 Dipartimento di Neuroscienze, Psicologia, Area del Farmaco e Salute del Bambino (NEUROFARBA), Università di Firenze Strutture Organizzative Dipartimentali (SOD) Gastroenterologia e Nutrizione Ospedale pediatrico Meyer, Firenze, Italy.
101 Department of Immunology, QIMR Berghofer Medical Research Institute, Brisbane, Australia.
102 University of Queensland School of Public Health, Brisbane, Australia.
103 Statistical Genetics, Department of Genetics and Computational Biology, QIMR Berghofer Medical Research Institute, Brisbane, Australia.
104 Department of Gastroenterology, The Townsville Hospital, Townsville, Australia.
105 Digestive Disease Research Institute, Shariati Hospital, Tehran, Iran.
106 Digestive Disease Research Center, Digestive Disease Research Institute, Tehran University of Medical Science, Tehran, Iran.
107 Digestive Disease Research Institute, Tehran University of Medical Sciences, Tehran, Iran.
108 Department of Gastroenterology & Hepatology, Royal Victoria Infirmary, Newcastle-upon-Tyne, UK.
109 Inflammatory Bowel Disease Research Group, Addenbrookes Hospital, Cambridge, UK.
110 Department of Medical and Molecular Genetics, Kings College London School of Medicine, Guys Hospital, London, UK.
111 Department of Gastroenterology, Fukuoka University Chikushi Hospital, Fukuoka, Japan.
112 Department of Gastroenterology, Leiden University Medical Center, Leiden, The Netherlands.
113 Dayanand Medical College and Hospital, Ludhiana, India.
114 Inflammatory Bowel Disease Centre, Mount Sinai Hospital, Toronto, Ontario, Canada.
115 Center for Human Molecular Genetics and Pharmacogenomics, Faculty of Medicine, University of Maribor, Maribor, Slovenia.
116 Molecular Epidemiology, University of Queensland Genome Innovation Hub, University of Queensland Institute for Molecular Bioscience, Brisbane, Australia.
117 Department of Medicine, Ninewells Hospital and Medical School, Dundee, UK.
118 Institut für Pathologie, Universität Bern.

119 Department of Medicine and Therapeutics, Institute of Digestive Disease, Chinese University of Hong Kong, Hong Kong.
120 Center for Computational and Integrative Biology, Massachusetts General Hospital, Harvard Medical School, Boston, Massachusetts, USA.
121 Department of Genomics Life & Brain Center, University Hospital Bonn, Bonn, Germany.
122 Division of Gastroenterology, Hepatology and Nutrition, Department of Pediatrics, Emory University School of Medicine, Atlanta, USA.
123 St Marys Hospital, London, UK.
124 Department of Gastroenterology, Academic Medical Center, Amsterdam, The Netherlands.
125 Icahn School of Medicine, Mount Sinai New York, New York, USA.
126 Faculty for Chemistry and Chemical Engineering, University of Maribor, Maribor, Slovenia.
127 Inflammatory Bowel Diseases, Department of Immunology, QIMR Berghofer Medical Research Institute, Brisbane, Australia.
128 Department of Gastroenterology, Royal Brisbane and Womens Hospital, Brisbane, Australia.
129 University of Queensland Faculty of Medicine Brisbane Australia.
130 Department of Gastroenterology, Université Catholique de Louvain (UCL) Centre Hospitalier Universitaire (CHU) Mont- Godinne, Mont-Godinne, Belgium.
131 Department Internal Medicine III, Division Gastroenterology & Hepatology, Medical University Vienna, Vienna, Austria.
132 UniversitätsSpital Zürich, Klinik für Gastroenterologie und Hepatologie, Zürich.
133 Paediatric Gastroenterology and Nutrition, Royal Hospital for Sick Children, Edinburgh, UK.
134 Department of Gastroenterology, St Thomas Hospital, London, UK.
135 Department of Digestive Diseases, Hospital Quiron Teknon, Barcelona, Spain.
136 Department of Public Health Sciences, University of Chicago, Chicago, Illinois, USA.
137 Human Genetics, Genome Institute of Singapore, Singapore.
138 Institute for Human Genetics, University of California, San Francisco, San Francisco, California, USA.
139 Department of Biology of Radiations and Human Health, Agenzia nazionale per le nuove tecnologie lenergia e lo sviluppo economico sostenibile (ENEA), Rome, Italy.
140 Laboratory for Statistical and Translational Genetics, Center for Integrative Medical Sciences, Riken, Yokohama, Japan.
141 Department of Clinical Science Intervention and Technology, Karolinska Institutet, Stockholm, Sweden.
142 Department of Medicine, University of Puerto Rico Center for IBD, University of Puerto Rico School of Medicine, Rio Piedras, Puerto Rico.
143 Gastroenterology & General Medicine, Norfolk and Norwich University Hospital, Norwich, UK.
144 Translational Gastroenterology Unit, Nuffield.
145 Digestive Disease Research Center, Digestive Disease Research Institute, Tehran University of Medical Sciences, Tehran Iran.
146 Department of Chronic Diseases, Metabolism & ageing, Translational Research in GastroIntestinal Disorders (TARGID), Katholieke Universiteit (KU) Leuven, Leuven, Belgium.
147 Division of Gastroenterology, University Hospital Gasthuisberg, Leuven, Belgium.
148 Faculty of Medicine, University of Toronto, Toronto, Ontario, Canada.

149 Meyerhoff Inflammatory Bowel Disease Center, Department of medicine, Johns Hopkins University School of Medicine, Baltimore, Maryland, USA.
150 Department of Gastroenterology and Hepatology, University of Groningen and University Medical Center Groningen, Groningen, The Netherlands.
151 Department of Computer Science, New Jersey Institute of Technology, Newark, New Jersey, USA.
152 Institute of Human Genetics, Technische Universität München, Munich, Germany.
153 Department of Neurology, Technische Universität München, Munich, Germany.
154 Department of Artificial Intelligence Medicine, Graduate School of Medicine, Chiba University, Chiba, Japan.
155 Department of Gastroenterology, Asan Medical Center, University of Ulsan College of Medicine, Seoul, Korea.
156 Department of Medicine I, University Medical Center Dresden, Technische Universität (TU) Dresden, Dresden, Germany.
157 Department of Biostatistics, School of Public Health, Yale University, NewHaven, Connecticut, USA.
158 Department of Gastroenterology, West China Hospital, Chengdu, Sichuan, China.
159 State Key Laboratory of Biotherapy, Sichuan University West China University of Medical Sciences (WCUMS), Chengdu, Sichuan, China.

## Members of the International PSC Study Group (IPSCSG)

Hugh Harley[1], Dep Huynh[1], Peter Fickert[2], Michael Trauner[3], Emina Halilbasic[3], Johan Fevery[4], Werner Van Steenbergen[5], Isabelle Cleynen[6], Severine Vermeire[7], Schalk Van der Merwe[8], Henriette Ytting[9], Andrew Mason[10], Bertus Eksteen[10], Martti Farkkila[11], Chantal Housset[12], Olivier Chazouillères[12], Raoul Poupon[12], Christophe Corpechot[12], Tobias Weismüller[13], Verena Keitel[14], Dieter Häussinger[14], Christoph Schramm[15], Ansgar Lohse[15], Michael E Manns[16], Tim Lankisch[16], Daniel Gotthardt[17], Peter Sauer[17], Peter Schirmacher[18], Beate K. Straub[18], Vincent Zimmer[19], Andre Franke[20], Eva Ellinghaus[20], David Ellinghaus[20], Stefan Schreiber[20,21,22], Ruben Plentz[23], Hugh E. Mulcahy[24], Einar Björnsson[25], Oren Shibolet[26], Marco Marzioni[27], Pietro Invernizzi[28], Ana Lleo[28], Carlo Selmi[29], Luca Fabris[30], Annarosa Floreani[30], Atsushi Tanaka[31], Hiromasa Ohira[32], Yoshiyuki Ueno[33], Tom H Karlsen[34], Erik Schrumpf[34], Kirsten M Boberg[34], Lars Aabakken[34], Espen Melum[34], Johannes Hov[34], Trine Folseraas[34], Mette Vesterhaug[35], Piotr Milkiewicz[36], Ewa Wunsch[36], Andrzej Habior[37], Albert Pares[38], Alexander Knuth[39], Beat Müllhaupt[40], Andreas Geier[40], Joachim C. Mertens[41], Annika Bergquist[42], Sven Almer[43], Lina Lindstrøm[43], Niklas Björkström[42], Fredrik Rorsman[43], Maria Benito de Valle Villalba[44], Hanns-Ulrich Marschall[44], Peter L. Jansen[45], Ulrich Beuers[45], Cyriel Y. Ponsioen[45], Andreas E. Kremer[45], Serge Zweers[45], Rinse K Weersma[46], Frank G. Schaap[47], Henk R. van Buuren[48], David H. Adams[49], Gideon M Hirschfield[49], Evangelia Liaskou[49], Arthur Kaser[50], Graeme J. Alexander[51], George F. Mells[52], Richard N Sandford[52], Carl A Anderson[53], Jimmy Z Liu[53], Stephen Pereira[54], George Webster[55], Andrew Burroughs[56], Shahid A Khan[57], Simon D. Taylor-Robinson[58], David Jones[59], Alastair Burt[60], Simon M Rushbrook[61], Roger W Chapman[62], Kate Williamson[62], Emma L. Culver[63], Said Al Mammari[62], Keith Lindor[64,65], Christopher L Bowlus[66], David Shapiro[67], Mario Strazzabosco[68], Greg Everson[69], Steve Helmke[69], Cynthia Levy[70], Konstantinos N Lazaridis[71], Brian D Juran7[71], Gregory Gores[72], Jayant Talwalkar[65], Nataliya Razumilava[72], David Goldberg[73], Dennis Black[74], Saul J. Karpen[75].

[1] University of Adelaide, Head of Clinical Hepatology, Co-Director of Viral Hepatitis Centre, Royal Adelaide Hospital, Australia.
[2] Division of Gastroenterology and Hepatology, Dept. of Medicine, Medical University of Graz, Graz, Austria.
[3] Department of Internal Medicine III, Divison of Gastroenterology and Hepatology Medical University of Vienna Waehringer, Vienna, Austria.
[4] Laboratory of Hepatology, University Hospital Gasthuisberg, Leuven, Belgium.
[5] Department of Liver, Biliary and Pancreatic Diseases, University Hospital Gasthuisberg, Catholic University of Leuven, Leuven, Belgium.
[6] Department of Pathophysiology, KU Leuven, Herestraat 49, box 701, 3000.
[7] Department of Gastroenterology, University Hospital Gasthuisberg, Herestraat 49, 3000 Leuven, Belgium
[8] Laboratory of Hepatology, University Hospital Gasthuisberg, Leuven, Belgium.
[9] Hepatologisk Klinik A-2121, Rigshospitalet, Københavns Universitet, Blegdamsvej 9, 2100 København Ø, Denmark.
[10] Snyder Institute for Chronic Diseases, Health Research and Innovation Centre (HRIC), 4AC66 – 3280 Hospital Drive NW, Calgary, Alberta, T2N 4N1, Canada.
[11] Division of Gastroenterology, Department of Medicine, Helsinki University Central Hospital, P.O. Box 340, 00029 HYKS, Finland.
[12] Service d'Hépatologie, Centre de référence des Maladies Inflammatoires des Voies Biliaires, Hôpital Saint-Antoine and UMR_S938, Faculté de Médecine Saint-Antoine, Université Pierre et Marie Curie (UPMC), Paris, France.
[13] Department of Internal Medicine 1, University Hospital of Bonn, Sigmund-Freud-Str. 25, 53127 Bonn, Germany.
[14] Department of Internal Medicine, Gastroenterology, Hepatology and Infectiology, Heinrich Heine Universität, Moorenstrasse 5, D-40225 Düsseldorf, Germany.
[15] 1st Department of Medicine, University Medical Center Hamburg-Eppendorf, Hamburg, Germany.

[16] Department of Gastroenterology Hepatology and Endocrinology, Hannover Medical School, Hannover, Germany.
[17] Department of Internal Medicine IV, University Hospital of Heidelberg, Im Neuenheimer Feld 410, 69120 Heidelberg, Germany.
[18] Department of General Pathology, Institute of Pathology, Heidelberg, Germany.
[19] Klinik für Innere Medizin II, Universitätsklinikum des Saarlandes, Kirrberger Straße 100, D-66421 Homburg, Germany.
[20] Institute of Clinical Molecular Biology, Christian-Albrechts-University of Kiel, Kiel, Germany.
[21] Department for General Internal Medicine, Christian-Albrechts-University of Kiel, Kiel, Germany.
[22] PopGen Biobank, University Hospital Schleswig-Holstein, Christian-Albrechts-University of Kiel, Kiel, Germany.
[23] Medizinische Universitätsklinik, Abteilung Innere Medizin 1, Tübingen, Germany.
[24] Centre for Colorectal Diseases, St Vincent's University Hospital, Dublin, Ireland.
[25] Department of Internal Medicine, Division of Gastroenterology and Hepatology, Landspitali University Hospital, Reykjavik, Iceland.
[26] Department of Gastroenterology, Tel-Aviv Medical Center and Tel-Aviv University, 6 Weizman St. Tel-Aviv 64239, Israel.
[27] Department of Gastroenterology, Università Politecnica delle Marche, Ospedali Riuniti University Hospital, Ancona, Italy.
[28] Center for Autoimmune Liver Diseases, Department of Medicine, IRCCS Istituto Clinico Humanitas, Milan, Italy.
[29] Department of Translational Medicine, University of Milan, IRCCS Istituto Clinico Humanitas, Milan, Italy.
[30] Department of Surgical and Gastroenterological Sciences, University of Padua, Padua, Italy.
[31] Department of Medicine, Teikyo University School of Medicine, Tokyo, Japan.
[32] Department of Gastroenterology and Rheumatology, Fukushima Medical University School of Medicine, Fukushima, Japan.
[33] Division of Gastroenterology, Graduate School of Medicine, Tohoku University, Sendai, Japan.
[34] Norwegian PSC Research Center, Division of Cancer, Surgery and Transplantation, Oslo University Hospital Rikshospitalet, P.O.box 4950 Nydalen, NO-0424 Oslo.
[35] Section for Gastroenterology, Dept. of Medicine, Haukeland University Hospital, N-5021 Bergen, Norway.
[36] Liver Unit, Pomeranian Medical University, SPSK2, Powstancow Wlkp 72, 70-111 Szczecin, Poland.
[37] Department of Gastroenterology and Hepatology, Medical Center for Postgraduate Education at the Maria Skłodowska-Curie Memorial Cancer Center, Institute of Oncology, Warsaw, Poland.
[38] Liver Unit, Hospital Clínic i Provincial, Villarroel 170, 08036-Barcelona, Spain.
[39] Clinic and Policlinic for Oncologie, University Hospital Zürich, Zürich, Switzerland.
[40] Department of Hepatology and Gastroenterology, Swiss Hepato-Pancreatico-Biliary (HPB) and Transplant Center, University Hospital Zurich, Zurich, Switzerland.
[41] Division of Gastroenterology and Hepatology, University Hospital Zürich, Zürich, Switzerland.
[42] Gastrocentrum Medicin, Karolinska Univ, Stockholm, Sweden.
[43] Dept of Gastroenterology and Hepatology, Uppsala Univeristy Hospital, Uppsala, Sweden.
[44] Sahlgrenska University Hospital, Per Dubbsgatan 15, 41345 Gothenburg, Sweden.
[45] Department of Gastroenterology & Hepatology, Tytgat Institute for Liver and Intestinal Research, Academic Medical Center, University of Amsterdam, The Netherlands.
[46] Department of Gastroenterology and Hepatology, Erasmus University Medical Centre, Rotterdam, The Netherlands.
[47] Health, Medicine and Life Sciences, NUTRIM School for Nutrition Toxicology and Metabolism, P.O.BOX 616, 6200 Maastricht.
[48] Health Department of Gastroenterology and Hepatology, Erasmus University Medical Centre, Room Ha-203, PO Box 2040, 3000 CA Rotterdam.
[49] Centre for Liver Research, MRC Centre for Immune Regulation, Institute for Biomedical Research, University of Birmingham, Birmingham, B15 2TT, UK.

[50] Section of Gastroenterology and Hepatology Medical Department, Addenbrooke's Hospital, Cambridge, UK.
[51] Department of Medicine, University of Cambridge School of Clinical Medicine, Addenbrooke's NHS Trust, Hills Road, Cambridge CB2 2QQ, UK.
[52] Academic Department of Medical Genetics, University of Cambridge, Addenbrooke's Hospital, Cambridge, UK.
[53] Department of Human Genetics, Wellcome Trust Sanger Institute, Wellcome Trust Genome Campus, Hinxton, CB10 1SA, England, UK.
[54] The UCL Institute of Hepatology, Royal Free Hospital, London, UK.
[55] Department of Gastroenterology, University College Hospital, London, UK.
[56] Sheila Sherlock Liver Centre and Department of Surgery, Royal Free Hospital, London, UK.
[57] Department of Gastroenterology, Hammersmith Hospital Campus, London, UK.
[58] Department of Medicine, St Mary's Hospital Campus, Imperial College London, UK.
[59] Institute of Cellular Medicine (Hepatology), The Medical School, Newcastle University, Newcastle upon Tyne, UK.
[60] Clinical Deanery, Faculty of Medical Sciences, Newcastle University, Newcastle, UK.
[61] Norfolk and Norwich University Hospital, Colney Lane, Norwich NR4 7UY, UK.
[62] Gastroenterology Department, John Radcliffe Hospital, Oxford University Hospitals, Oxford, UK.
[63] Translational Gastroenterology Unit, John Radcliffe Hospital, Oxford Radcliffe NHS Trust, Oxford OX3.
[64] Arizona State University, 500 N. 3rd Street, Phoenix, AZ 85004-0698.
[65] Division of Gastroenterology and Hepatology, Mayo Clinic, W9A, 200 First Street, SW, Rochester, MN 55905, USA
[66] Department of Internal Medicine, University of California Davis Medical Center, 4150 V Street, PSSB 3100, Sacramento, CA 95817, USA
[67] 4350 La Jolla Village Drive Suite 960, San Diego, CA 92122, USA.
[68] Department of Internal Medicine Section of Digestive Diseases Yale University, New Haven, CT, USA.
[69] Section of Hepatology, Hepatology Clinic, University of Colorado, 1635 Aurora Ct., Aurora, CO 80045.
[70] Center for Liver Diseases, Division of Hepatology, University of Miami Miller School of Medicine, FL.
[71] Center for Basic Research in Digestive Diseases, Division of Gastroenterology and Hepatology, Mayo Clinic College of Medicine, 200 First Street SW, Rochester MN 55905, USA
[72] College of Medicine, Mayo Clinic, Rochester, MN, USA.
[73] Division of Gastroenterology, Dept. of Medicine, Hospital of the University of Pennsylvania and Perelman Center for Advanced Medicine, USA.
[74] Memphis Medical Center, Memphis, Tennessee, USA.
[75] Department of Pediatrics, Baylor College of Medicine, Houston, Texas, USA.

# References


1. Yang, J., *et al.* Conditional and joint multiple-SNP analysis of GWAS summary statistics identifies additional variants influencing complex traits. *Nat Genet* **44**, 369-375, S361-363 (2012).
2. Liu, J.Z., *et al.* Association analyses identify 38 susceptibility loci for inflammatory bowel disease and highlight shared genetic risk across populations. *Nat Genet* **47**, 979-986 (2015).
3. Huang, H., *et al.* Fine-mapping inflammatory bowel disease loci to single-variant resolution. *Nature* **547**, 173-178 (2017).
4. Pardinas, A.F., *et al.* Common schizophrenia alleles are enriched in mutation-intolerant genes and in regions under strong background selection. *Nat Genet* **50**, 381-389 (2018).
5. Liu, Y., *et al.* LRH1 as a promising prognostic biomarker and predictor of metastasis in patients with non-small cell lung cancer. *Thorac Cancer* **9**, 1725-1732 (2018).
6. Xiao, L., *et al.* Nuclear Receptor LRH-1 Functions to Promote Castration-Resistant Growth of Prostate Cancer via Its Promotion of Intratumoral Androgen Biosynthesis. *Cancer Res* **78**, 2205-2218 (2018).
7. Bayrer, J.R., Mukkamala, S., Sablin, E.P., Webb, P. & Fletterick, R.J. Silencing LRH-1 in colon cancer cell lines impairs proliferation and alters gene expression programs. *Proceedings of the National Academy of Sciences of the United States of America* **112**, 2467-2472 (2015).
8. Ueno, M., *et al.* Genome-wide association study-identified SNPs (rs3790844, rs3790843) in the NR5A2 gene and risk of pancreatic cancer in Japanese. *Scientific reports* **5**, 17018 (2015).
9. Bianco, S., Brunelle, M., Jangal, M., Magnani, L. & Gevry, N. LRH-1 governs vital transcriptional programs in endocrine-sensitive and -resistant breast cancer cells. *Cancer Res* **74**, 2015-2025 (2014).
10. Seitz, C., *et al.* The orphan nuclear receptor LRH-1/NR5a2 critically regulates T cell functions. *Sci Adv* **5**, eaav9732 (2019).
11. Ahmed, A., Schwaderer, J., Hantusch, A., Kolho, K.L. & Brunner, T. Intestinal glucocorticoid synthesis enzymes in pediatric inflammatory bowel disease patients. *Genes Immun* **20**, 566-576 (2019).
12. Bouguen, G., *et al.* Intestinal steroidogenesis controls PPARgamma expression in the colon and is impaired during ulcerative colitis. *Gut* **64**, 901-910 (2015).
13. Coste, A., *et al.* LRH-1-mediated glucocorticoid synthesis in enterocytes protects against inflammatory bowel disease. *Proceedings of the National Academy of Sciences of the United States of America* **104**, 13098-13103 (2007).
14. Huang, S.C., Lee, C.T. & Chung, B.C. Tumor necrosis factor suppresses NR5A2 activity and intestinal glucocorticoid synthesis to sustain chronic colitis. *Sci Signal* **7**, ra20 (2014).
15. Bayrer, J.R., *et al.* LRH-1 mitigates intestinal inflammatory disease by maintaining epithelial homeostasis and cell survival. *Nature communications* **9**, 4055 (2018).
16. Stergiopoulos, A. & Politis, P.K. Nuclear receptor NR5A2 controls neural stem cell fate decisions during development. *Nature communications* **7**, 12230 (2016).
17. Alvarez-Bolado, G. Development of neuroendocrine neurons in the mammalian hypothalamus. *Cell Tissue Res* **375**, 23-39 (2019).
18. Narayan, S., *et al.* Molecular profiles of schizophrenia in the CNS at different stages of illness. *Brain Res* **1239**, 235-248 (2008).
19. Fagerberg, L., *et al.* Analysis of the human tissue-specific expression by genome-wide integration of transcriptomics and antibody-based proteomics. *Mol Cell Proteomics* **13**, 397-406 (2014).
20. Zarate, Y.A. & Fish, J.L. SATB2-associated syndrome: Mechanisms, phenotype, and practical recommendations. *Am J Med Genet A* **173**, 327-337 (2017).
21. Li, M., *et al.* Integrative functional genomic analysis of human brain development and neuropsychiatric risks. *Science* **362**(2018).
22. FitzPatrick, D.R., *et al.* Identification of SATB2 as the cleft palate gene on 2q32-q33. *Hum Mol Genet* **12**, 2491-2501 (2003).
23. Wang, S., *et al.* Down-regulated expression of SATB2 is associated with metastasis and poor prognosis in colorectal cancer. *J Pathol* **219**, 114-122 (2009).
24. Magnusson, K., *et al.* SATB2 in combination with cytokeratin 20 identifies over 95% of all colorectal carcinomas. *Am J Surg Pathol* **35**, 937-948 (2011).
25. Iwaya, M., *et al.* Colitis-associated colorectal adenocarcinomas are frequently associated with non-intestinal mucin profiles and loss of SATB2 expression. *Mod Pathol* **32**, 884-892 (2019).
26. Gui, X., *et al.* Histological and molecular diversity and heterogeneity of precancerous lesions associated with inflammatory bowel diseases. *J Clin Pathol* **73**, 391-402 (2020).
27. Gui, X., *et al.* Newly recognized non-adenomatous lesions associated with enteric carcinomas in inflammatory bowel disease - Report of six rare and unique cases. *Ann Diagn Pathol* **44**, 151455 (2020).
28. Whitton, L., *et al.* Genes regulated by SATB2 during neurodevelopment contribute to schizophrenia and educational attainment. *PLoS genetics* **14**, e1007515 (2018).
29. Zhou, J., *et al.* Common variants in SATB2 are associated with schizophrenia in Uygur Chinese population. *Psychiatr Genet* **29**, 120-126 (2019).
30. Whitton, L., *et al.* Cognitive analysis of schizophrenia risk genes that function as epigenetic regulators of gene expression. *Am J Med Genet B Neuropsychiatr Genet* **171**, 1170-1179 (2016).
31. Ohi, K., *et al.* Specific gene expression patterns of 108 schizophrenia-associated loci in cortex. *Schizophr Res* **174**, 35-38 (2016).

32. Wang, M.G., Yi, H., Guerini, D., Klee, C.B. & McBride, O.W. Calcineurin A alpha (PPP3CA), calcineurin A beta (PPP3CB) and calcineurin B (PPP3R1) are located on human chromosomes 4, 10q21-->q22 and 2p16-->p15 respectively. *Cytogenet Cell Genet* **72**, 236-241 (1996).
33. Clipstone, N.A. & Crabtree, G.R. Calcineurin is a key signaling enzyme in T lymphocyte activation and the target of the immunosuppressive drugs cyclosporin A and FK506. *Ann N Y Acad Sci* **696**, 20-30 (1993).
34. Jain, J., *et al.* The T-cell transcription factor NFATp is a substrate for calcineurin and interacts with Fos and Jun. *Nature* **365**, 352-355 (1993).
35. Woodrow, M., Clipstone, N.A. & Cantrell, D. p21ras and calcineurin synergize to regulate the nuclear factor of activated T cells. *J Exp Med* **178**, 1517-1522 (1993).
36. Yoo, S.A., *et al.* Calcineurin is expressed and plays a critical role in inflammatory arthritis. *J Immunol* **177**, 2681-2690 (2006).
37. Mok, C.C. Calcineurin inhibitors in systemic lupus erythematosus. *Best Pract Res Clin Rheumatol* **31**, 429-438 (2017).
38. Matsuoka, K., *et al.* Tacrolimus for the Treatment of Ulcerative Colitis. *Intest Res* **13**, 219-226 (2015).
39. Gerber, D.J., *et al.* Evidence for association of schizophrenia with genetic variation in the 8p21.3 gene, PPP3CC, encoding the calcineurin gamma subunit. *Proceedings of the National Academy of Sciences of the United States of America* **100**, 8993-8998 (2003).
40. Miyakawa, T., *et al.* Conditional calcineurin knockout mice exhibit multiple abnormal behaviors related to schizophrenia. *Proceedings of the National Academy of Sciences of the United States of America* **100**, 8987-8992 (2003).
41. Takao, K. & Miyakawa, T. Investigating gene-to-behavior pathways in psychiatric disorders: the use of a comprehensive behavioral test battery on genetically engineered mice. *Ann N Y Acad Sci* **1086**, 144-159 (2006).
42. Wada, A., *et al.* Prominent increased calcineurin immunoreactivity in the superior temporal gyrus in schizophrenia: A postmortem study. *Psychiatry Res* **247**, 79-83 (2017).
43. Barksdale, K.A., Lahti, A.C. & Roberts, R.C. Synaptic proteins in the postmortem anterior cingulate cortex in schizophrenia: relationship to treatment and treatment response. *Neuropsychopharmacology* **39**, 2095-2103 (2014).
44. Kozlovsky, N., Scarr, E., Dean, B. & Agam, G. Postmortem brain calcineurin protein levels in schizophrenia patients are not different from controls. *Schizophr Res* **83**, 173-177 (2006).
45. Wada, A., *et al.* Increased ratio of calcineurin immunoreactive neurons in the caudate nucleus of patients with schizophrenia. *Prog Neuropsychopharmacol Biol Psychiatry* **37**, 8-14 (2012).
46. Kunii, Y., *et al.* Elevated postmortem striatal t-DARPP expression in schizophrenia and associations with DRD2/ANKK1 polymorphism. *Prog Neuropsychopharmacol Biol Psychiatry* **53**, 123-128 (2014).
47. Wang, Y., *et al.* Single-cell transcriptome analysis reveals differential nutrient absorption functions in human intestine. *J Exp Med* **217**(2020).
48. Martin, J.C., *et al.* Single-Cell Analysis of Crohn's Disease Lesions Identifies a Pathogenic Cellular Module Associated with Resistance to Anti-TNF Therapy. *Cell* **178**, 1493-1508 e1420 (2019).
49. Worthington, J.J., Reimann, F. & Gribble, F.M. Enteroendocrine cells-sensory sentinels of the intestinal environment and orchestrators of mucosal immunity. *Mucosal Immunol* **11**, 3-20 (2018).
50. Wang, L., *et al.* SF3B1 and other novel cancer genes in chronic lymphocytic leukemia. *N Engl J Med* **365**, 2497-2506 (2011).
51. Maguire, S.L., *et al.* SF3B1 mutations constitute a novel therapeutic target in breast cancer. *J Pathol* **235**, 571-580 (2015).
52. Jimenez-Vacas, J.M., *et al.* Spliceosome component SF3B1 as novel prognostic biomarker and therapeutic target for prostate cancer. *Transl Res* **212**, 89-103 (2019).
53. Yang, H.M., *et al.* Identification of recurrent mutational events in anorectal melanoma. *Mod Pathol* **30**, 286-296 (2017).
54. Ingason, A., *et al.* Expression analysis in a rat psychosis model identifies novel candidate genes validated in a large case-control sample of schizophrenia. *Transl Psychiatry* **5**, e656 (2015).
55. De Arras, L. & Alper, S. Limiting of the innate immune response by SF3A-dependent control of MyD88 alternative mRNA splicing. *PLoS genetics* **9**, e1003855 (2013).
56. Cox, E., *et al.* Global Analysis of SUMO-Binding Proteins Identifies SUMOylation as a Key Regulator of the INO80 Chromatin Remodeling Complex. *Mol Cell Proteomics* **16**, 812-823 (2017).
57. Poli, J., Gasser, S.M. & Papamichos-Chronakis, M. The INO80 remodeller in transcription, replication and repair. *Philos Trans R Soc Lond B Biol Sci* **372**(2017).
58. Liu, H., *et al.* Integrated Analysis of Summary Statistics to Identify Pleiotropic Genes and Pathways for the Comorbidity of Schizophrenia and Cardiometabolic Disease. *Front Psychiatry* **11**, 256 (2020).
59. Gusev, A., *et al.* Transcriptome-wide association study of schizophrenia and chromatin activity yields mechanistic disease insights. *Nat Genet* **50**, 538-548 (2018).
60. Quaglio, A.E., Castilho, A.C. & Di Stasi, L.C. Experimental evidence of MAP kinase gene expression on the response of intestinal anti-inflammatory drugs. *Life Sci* **136**, 60-66 (2015).
61. Li, X.L., *et al.* Bioinformatic analysis of potential candidates for therapy of inflammatory bowel disease. *Eur Rev Med Pharmacol Sci* **19**, 4275-4284 (2015).
62. Tan, T., Zhang, K. & Chen, W. Genetic variants of ESR1 and SGSM3 are associated with the susceptibility of breast cancer in the Chinese population. *Breast Cancer* **24**, 369-374 (2017).
63. Hashemi, M., *et al.* Evaluation of 4-bp insertion/deletion polymorphism within the 3'UTR of SGSM3 in bladder cancer using mismatch PCR-RFLP method: A preliminary report. *J Cell Biochem* **119**, 6566-6574 (2018).

64. Marques, D., *et al.* Association of insertion-deletions polymorphisms with colorectal cancer risk and clinical features. *World J Gastroenterol* **23**, 6854-6867 (2017).
65. Lee, C.Y., *et al.* Interaction of small G protein signaling modulator 3 with connexin 43 contributes to myocardial infarction in rat hearts. *Biochem Biophys Res Commun* **491**, 429-435 (2017).
66. Joo, H.C., *et al.* Protective effects of kenpaullone on cardiomyocytes following H2O2-induced oxidative stress are attributed to inhibition of connexin 43 degradation by SGSM3. *Biochem Biophys Res Commun* **499**, 368-373 (2018).
67. Vitour, D., Lindenbaum, P., Vende, P., Becker, M.M. & Poncet, D. RoXaN, a novel cellular protein containing TPR, LD, and zinc finger motifs, forms a ternary complex with eukaryotic initiation factor 4G and rotavirus NSP3. *J Virol* **78**, 3851-3862 (2004).
68. Antonescu, C.R., *et al.* Novel ZC3H7B-BCOR, MEAF6-PHF1, and EPC1-PHF1 fusions in ossifying fibromyxoid tumors--molecular characterization shows genetic overlap with endometrial stromal sarcoma. *Genes Chromosomes Cancer* **53**, 183-193 (2014).
69. Astolfi, A., *et al.* BCOR involvement in cancer. *Epigenomics* **11**, 835-855 (2019).
70. Barbeira, A.N., *et al.* Exploring the phenotypic consequences of tissue specific gene expression variation inferred from GWAS summary statistics. *Nature communications* **9**, 1825 (2018).
71. Gusev, A., *et al.* Integrative approaches for large-scale transcriptome-wide association studies. *Nat Genet* **48**, 245-252 (2016).
