# Supplementary material for: Cross-tissue transcriptome-wide association studies identify susceptibility genes shared between schizophrenia and inflammatory bowel disease
Source: Commun Biol. 2022 Jan 20;5:80. doi: 10.1038/s42003-022-03031-6 (PMC8776955; doi:10.1038/s42003-022-03031-6)
Supplement: Supplementary file 3 — Description of Additional Supplementary Files [file 42003_2022_3031_MOESM3_ESM.pdf]

Description of Additional Supplementary Files

File name: Supplementary Data 1
Description: GWAS case/control panels (summary statistics) used for TWAS analysis

File name: Supplementary Data 2
Description: Number of samples per tissue and successfully tested genes from UTMOST cross-tissue expression imputation models

File name: Supplementary Data 3
Description: Marginal (i.e. unconditioned) and conditioned UTMOST TWAS association results

File name: Supplementary Data 4
Description: Moderate correlation was observed between sample size and number of significant genes

File name: Supplementary Data 5
Description: Genomic inflation factor values for transcriptome-wide association analysis of the generalized Berk-Jones test across 23 tissues of the gut-brain-axis

File name: Supplementary Data 6
Description: Number of gene discoveries from cross-tissue multiple-gene-conditioned transcriptome-wide association analysis across 23 tissues of the gut-brain-axis.

File name: Supplementary Data 7
Description: Position of known GWAS lead SNPs and 0.1 cM locus boundaries of the seven traits studied.

File name: Supplementary Data 8
Description: Comorbidity analysis in the Danish National Patient Registry (DNPR) and ICD10 diagnose codes from 7,191,385 people of the entire Danish population in the period from 1996 to 2018

File name: Supplementary Data 9
Description: Genetic trait correlation and trait heritability calculated with LDSC for SCZ, BD, ADHD, MDD, UC, CD and PSC

File name: Supplementary Data 10
Description: Pair-wise trait correlations at the level of predicted gene expression (and LDSC regression) for all pairs of diseases and 23 tissues

File name: Supplementary Data 11
Description: Results from bi-directional GSMR (Generalised Summary-data-based Mendelian Randomisation) analysis

File name: Supplementary Data 12
Description: Gene-disease associations shared between psychiatric and inflammatory diseases identified by gene overlap analysis

File name: Supplementary Data 13
Description: Four susceptibility genes with GBJ-test transcriptome-wide significance are shared by

psychiatric and immune diseases of the gut-brain-axis (GBA), but each in different tissues for psychiatric and immune phenotypes

File name: Supplementary Data 14
Description: The three single-tissue conditional TWAS susceptibility genes NR5A2, SATB2 and PPP3CA are shared between SCZ and CD/UC

File name: Supplementary Data 15
Description: EnrichR identifies "Calcineurin-dependent NFAT signaling role in lymphocytes" in BioPlanet 2019 which is intertwined with Wnt-sinaling as probable common term

File name: Supplementary Data 16
Description: Data displayed in Figure 3

File name: Supplementary Data 17
Description: Data displayed in Figure 4

File name: Supplementary Data 18
Description: Data displayed in Figure 5
