# Supplementary material for: Cross-tissue transcriptome-wide association studies identify susceptibility genes shared between schizophrenia and inflammatory bowel disease
Source: Commun Biol. 2022 Jan 20;5:80. doi: 10.1038/s42003-022-03031-6 (PMC8776955; doi:10.1038/s42003-022-03031-6)
Supplement: Supplementary file 10 — Reporting Summary [file 42003_2022_3031_MOESM10_ESM.pdf]

nature research

Corresponding author(s): David Ellinghaus

Last updated by author(s): Nov 17, 2021

# Reporting Summary

Nature Research wishes to improve the reproducibility of the work that we publish. This form provides structure for consistency and transparency in reporting. For further information on Nature Research policies, see our Editorial Policies and the Editorial Policy Checklist.

## Statistics

For all statistical analyses, confirm that the following items are present in the figure legend, table legend, main text, or Methods section.

| n/a | Confirmed | |
|---|---|---|
| ☐ | ☒ | The exact sample size ($n$) for each experimental group/condition, given as a discrete number and unit of measurement |
| ☐ | ☒ | A statement on whether measurements were taken from distinct samples or whether the same sample was measured repeatedly |
| ☐ | ☒ | The statistical test(s) used AND whether they are one- or two-sided<br>*Only common tests should be described solely by name; describe more complex techniques in the Methods section.* |
| ☐ | ☒ | A description of all covariates tested |
| ☐ | ☒ | A description of any assumptions or corrections, such as tests of normality and adjustment for multiple comparisons |
| ☐ | ☒ | A full description of the statistical parameters including central tendency (e.g. means) or other basic estimates (e.g. regression coefficient) AND variation (e.g. standard deviation) or associated estimates of uncertainty (e.g. confidence intervals) |
| ☐ | ☒ | For null hypothesis testing, the test statistic (e.g. $F$, $t$, $r$) with confidence intervals, effect sizes, degrees of freedom and $P$ value noted<br>*Give P values as exact values whenever suitable.* |
| ☒ | ☐ | For Bayesian analysis, information on the choice of priors and Markov chain Monte Carlo settings |
| ☒ | ☐ | For hierarchical and complex designs, identification of the appropriate level for tests and full reporting of outcomes |
| ☐ | ☒ | Estimates of effect sizes (e.g. Cohen's $d$, Pearson's $r$), indicating how they were calculated |

*Our web collection on statistics for biologists contains articles on many of the points above.*

## Software and code

Policy information about availability of computer code

| | |
|---|---|
| Data collection | PGC results, https://www.med.unc.edu/pgc/download-results/<br>ftp://ftp.sanger.ac.uk/pub/consortia/ibdgenetics/iibdgc-trans-ancestry-filtered-summary-stats.tgz<br>BLUEPRINT, ftp://ftp.ebi.ac.uk/pub/databases/blueprint/<br>GTEx data, https://www.gtexportal.org/home/datasets<br>E-MTAB-6814, https://www.ebi.ac.uk/gxa/experiments/E-MTAB-6814/Downloads<br>E-MTAB-5015, https://www.ebi.ac.uk/gxa/experiments/E-MTAB-5015/Downloads<br>E-MTAB-3827, https://www.ebi.ac.uk/gxa/experiments/E-MTAB-3827/Downloads<br>https://www.covid19cellatlas.org |
| Data analysis | Bio Planet 2019 from Enrichr website https://maayanlab.cloud/Enrichr/#stats<br>ScanPy package, https://scanpy.readthedocs.io/en/stable/api/scanpy.pl.dotplot.html<br>https://github.com/Joker-Jerome/UTMOST<br>http://predictdb.org/<br>http://gusevlab.org/projects/fusion/<br>https://github.com/bulik/ldsc |

For manuscripts utilizing custom algorithms or software that are central to the research but not yet described in published literature, software must be made available to editors and reviewers. We strongly encourage code deposition in a community repository (e.g. GitHub). See the Nature Research guidelines for submitting code & software for further information.

## Data

Policy information about availability of data

All manuscripts must include a data availability statement. This statement should provide the following information, where applicable:

- Accession codes, unique identifiers, or web links for publicly available datasets
- A list of figures that have associated raw data
- A description of any restrictions on data availability

*Provide your data availability statement here.*

# Field-specific reporting

Please select the one below that is the best fit for your research. If you are not sure, read the appropriate sections before making your selection.

☒ Life sciences ☐ Behavioural & social sciences ☐ Ecological, evolutionary & environmental sciences

For a reference copy of the document with all sections, see nature.com/documents/nr-reporting-summary-flat.pdf

# Life sciences study design

All studies must disclose on these points even when the disclosure is negative.

| | |
|---|---|
| Sample size | Sample size and sample overlap was determined from previously described, QCed and imputed GWAS data sets. The GWAS data were re-analysed |
| Data exclusions | No data were excluded. |
| Replication | TWAS results for the CD, UC, and PSC phenotypes were replicated using additional datasets (see Suppl Data 1). Results for the SCZ, MDD, BIP, and ADHD phenotypes could not be verified a second time because here we collected the largest publicly available GWAS datasets for the primary TWAS analysis. |
| Randomization | GWAS/TWAS study subjects were divided into cases and controls as described in the original GWAS studies (see Suppl Data 1). |
| Blinding | Blinding was not relevant to our study because of a retrospective case-control study design. |

# Reporting for specific materials, systems and methods

We require information from authors about some types of materials, experimental systems and methods used in many studies. Here, indicate whether each material, system or method listed is relevant to your study. If you are not sure if a list item applies to your research, read the appropriate section before selecting a response.

## Materials & experimental systems

| n/a | Involved in the study | |
|---|---|---|
| ☒ | ☐ | Antibodies |
| ☒ | ☐ | Eukaryotic cell lines |
| ☒ | ☐ | Palaeontology and archaeology |
| ☒ | ☐ | Animals and other organisms |
| ☐ | ☒ | Human research participants |
| ☒ | ☐ | Clinical data |
| ☒ | ☐ | Dual use research of concern |

## Methods

| n/a | Involved in the study | |
|---|---|---|
| ☒ | ☐ | ChIP-seq |
| ☒ | ☐ | Flow cytometry |
| ☒ | ☐ | MRI-based neuroimaging |

## Human research participants

Policy information about studies involving human research participants

| | |
|---|---|
| Population characteristics | GWAS data from subjects were adjusted for age, sex and/or principal components from principal component analysis (PCA) as described in the original GWAS publications (see Suppl Data 1). |
| Recruitment | Participants were recruited as desribed in the the original GWAS publications. |
| Ethics oversight | Study protocol was approved by participant centres and ethic committees as described in the original GWAS publications. The GWAS data were re-analysed. |

Note that full information on the approval of the study protocol must also be provided in the manuscript.
